# Efficient Light‐Driven Multi‐Electron Accumulation on Viologens by Molecular Control Over Photochemical Reaction Pathways

**DOI:** 10.1002/chem.71467

**Published:** 2026-07-30

**Authors:** Fatemeh Sousani, Georgina E. Shillito, Alexander K. Mengele, Martin D. Hager, Ulrich S. Schubert, Stefanie Gräfe, Stephan Kupfer, Sven Rau

**Affiliations:** ^1^ Institute of Inorganic Chemistry Ulm University Ulm Germany; ^2^ Institute of Physical Chemistry and Abbe Center of Photonics Friedrich Schiller University Jena Jena Germany; ^3^ Laboratory of Organic and Macromolecular Chemistry (IOMC) Friedrich Schiller University Jena Jena Germany; ^4^ Jena Center for Soft Matter (JCSM) Friedrich Schiller University Jena Jena Germany; ^5^ Center For Energy and Environmental Chemistry Jena (CEEC Jena) Friedrich Schiller University Jena Jena Germany; ^6^ Helmholtz Institute for Polymers in Energy Applications Jena (HIPOLE Jena) Jena Germany; ^7^ Helmholtz Zentrum für Materialien Und Energie Berlin (HZB) Berlin Germany; ^8^ Fraunhofer Institute for Applied Optics and Precision Engineering Jena Germany

**Keywords:** *Ab initio* molecular dynamics, computational chemistry, photocatalysis, UV/vis spectroscopy

## Abstract

Achieving multiple photo‐induced charge accumulations on the same acceptor is one option to propel photochemical reactions that demand highly reducing or oxidizing conditions. By switching the initial light‐driven process from an oxidative to a reductive quenching pathway via mutual redox potential control over Ru(II) polypyridyl chromophores and sacrificial electron donors (SEDs), acceleration of complete single electron reduction of methyl viologen (MV^2+^) and polymer‐bound viologens by a factor of up to 2,200 as well as a rapid subsequent second reduction of MV^•+^ to MV^0^ was achieved in homogeneous solution. *Ab initio* molecular dynamics (AIMD) allowed the conformer space to be mapped and to shed light on the thermodynamics of the photochemical intermediates involved in the formation of the two‐electron reduced methyl viologen (MV^0^). These results provide a toolbox of structurally simple compounds for the modular construction of efficient artificial photosynthesis schemes.

## Graphical abstract

Minimum structural changes, maximum output: By either utilizing a more oxidizing Ru(II) polypyridyl chromophore or by using a more reducing SED, the photocatalytic reduction of methyl viologen (MV^2+^) proceeds *via* a reductive quenching pathway. This not only significantly accelerates the overall photoreduction process but also allows accumulation of twofold reduced MV^0^ in homogeneous bulk solution.

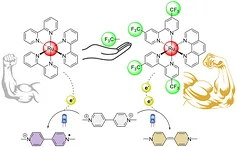

## 1 Introduction

Artificial photosynthesis requires the photo‐induced accumulation of multiple charges of suitable redox potential to drive multi‐electron reactions such as water splitting [1]. While nature and most artificial systems utilize proton‐coupled electron transfers (PCET) or other structural rearrangements of the acceptor [2] to keep the required potentials for multiple successive electron transfer (eT) steps similar [3, 4], PCET‐free multi‐electron collection to in contrast build up highly oxidizing or reducing systems for applications demanding more reactive intermediates remains challenging. With the molecular structure‐inherent unchangeable redox potentials of a given photosensitizer (PS) under nonvarying surrounding conditions, the rate for each subsequent forward eT process decreases in such systems due to reduced driving forces. Also, there is kinetic competition of an excited state being either unproductively quenched by the already charged acceptor versus the desired eT with the sacrificial agent (SA) [2, 5, 6]. Due to large driving forces for unproductive eT between PS* and already charged acceptors [2, 5], the productive eT storing another redox equivalent on the already charged acceptor *via* preceding charge uptake from the SA is kinetically unfavored [7].

Colorless methyl viologen (MV^2+^) has been used for decades as a model compound to study eT processes [7, 8] as well as an electron relay in photocatalytic systems for, for example, H_2_ evolution [9, 10, 11, 12]. Typically, only the one‐electron reduced, strongly colored radical cation (MV^•+^) is formed as, for example, shown in a recent report on polymer‐bound viologens [13]. Although in this case the mono‐reduced viologen state could be stored for many hours and acidification enabled an on‐demand H_2_ evolution, slow photochemical charging kinetics of the polymer utilizing a rather reducing Ru(II) polypyridyl complex represented a significant limitation for future applications.

In contrast to the well investigated formation of MV^•+^, the two‐electron reduced and yellow colored [14] species (MV^0^) has received comparably little attention despite being ca. 400 mV more reducing compared to MV^•+^ [7, 15] and thus allowing it to initiate a broader scope of reductive transformations [16]. So far, derivatives of MV^0^ have been used, for example, in redox‐flow batteries [17, 18, 19, 20] and were produced in some photocatalytic reactions *via* a light‐independent microenvironment‐driven disproportionation of monoreduced viologens at organic/aqueous interfaces [16, 21, 22, 23, 24]. The low number of reports on directly photogenerated MV^0^ in the absence of such interfaces is associated with the thermodynamics and kinetics discussed above, thus precluding photochemical accumulation of MV^0^ for often employed [Ru(bpy)_3_]^2+^‐based PSs (bpy = 2,2´‐bipyridine) [7], if no favorable supramolecular effects are exploited [25, 26], the ratio of organic solvent in a DMF/water mixture was large [27], or if stronger reducing PSs such as proflavine [28], TiO_2_ [29], or COFs [30] were used instead. In addition, formation of twofold reduced viologens in absence of an irreversibly degrading sacrificial electron donor (SED) has been observed by transient absorption spectroscopy [31, 32, 33].

In view of published work [34, 35, 36, 37, 38] showing efficient mono‐reduction of MV^2+^ to MV^•+^ by utilizing a strongly oxidizing Ru(II) polypyridyl complex engaging in reductive quenching with the different SEDs employed, and also following thermodynamic reasoning (see Schemes S1–S4; Tables S1 and S2), as well as kinetic [7] and cage escape considerations [39], we hypothesized that enabling reductive quenching between PS* and the SED leading at the same time to the generation of a photoreduced dye with sufficiently negative *E*_red_ should allow broadly applicable MV^0^ accumulation in homogeneous solution [32, 33]. To this end and as shown in Scheme 1, two structurally simple Ru(II) polypyridyl complexes differing in their redox potentials were combined with two different SEDs to initiate the reductive quenching pathway of the overall photocatalytic process. Formation of MV^0^ was followed by ground‐state UV/vis absorption and EPR spectroscopy and modelled by *ab initio* molecular dynamics (AIMD).

**SCHEME 1:**
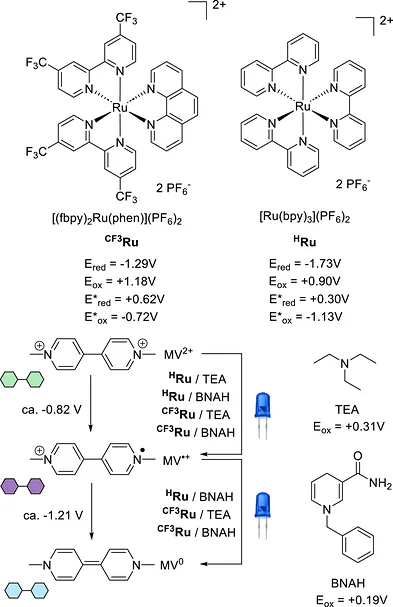
Structures of **^CF3^Ru** and **^H^Ru** (top), utilized SEDs (bottom right) and observed photoreactivity (bottom left). Potentials are given versus Fc^+^/Fc and were taken from literature [7, 42, 44, 45].

## 2 Results and Discussion

Following these considerations, it was first investigated, whether in the presence of triethylamine (TEA) as the SED the switch from oxidative quenching‐dominated MV^2+^ reduction to a reductive quenching pathway *via* increasing E*_red_ of prototype [Ru(bpy)_3_]^2+^ (**^H^Ru**) by incorporation of structurally simple electron‐withdrawing groups (EWGs) is possible [40]. For this purpose, that is to make the PS more oxidizing but keeping the overall molecular structure as simple as possible to enable broad applicability in the future, [(fbpy)_2_Ru(phen)](PF_6_)_2_ was selected (**^CF3^Ru**, fbpy = 4,4′‐di‐trifluoromethyl‐2,2′‐bipyridine [41], phen = 1,10‐phenanthroline, Scheme 1; for characterization *via* ^1^H, ^13^C, ^19^F, and mass spectrometry see Figures S1–S6) [42]. Luminescence quenching experiments with TEA (*E*_ox_ = 0.31 V) [43, 44] and MV^2+^ (*E*_red_ = ‐0.82 V) [7] proved an overall switch from oxidative quenching to reductive quenching when **^H^Ru** was replaced by **^CF3^Ru** (Figure S8), consistent with reported excited state potentials for these complexes (Scheme 1, all potentials vs. Fc^+^/Fc) [7, 41, 42, 45].

Next, visible light‐driven MV^2+^ reduction in MeCN:H_2_O = 1:1 (v:v, acetonitrile:water) in the presence of phosphate‐buffered TEA was performed using an LED array (*λ*_max_ = 463 nm; for experimental details, see the Supporting Information). Steady‐state UV/vis absorption spectra were recorded in appropriate time intervals (Figure 1a) resulting in plots of the characteristic absorbance of MV^•+^ at 605 nm versus irradiation time. In accordance with literature reports [34, 35, 36, 37, 38, 46], electron poorer **^CF3^Ru**—not undergoing oxidative quenching with MV^2+^—accelerated MV^2+^ reduction compared to **^H^Ru** by a factor of ca. 200 (23 s vs. 75 min, see Figure S9) for the complete conversion of MV^2+^ to MV^•+^. Based on the given times needed, the quantum yields (QYs) for these processes were ca. 12% for **^CF3^Ru** and ca. 0.06% for **^H^Ru**. At low MV^2+^ concentrations (100 µM), it is literature‐known for **^H^Ru** to give low QYs for the formation of MV^•+^ under comparable conditions using tertiary amines as SEDs [47]. Additionally, based on characteristic optical changes of the decreasing 605 nm band as well as the band at ca. 400 nm approximately retaining its intensity [25, 27, 48, 49, 50], only **^CF3^Ru** allowed subsequent accumulation of MV^0^. To additionally verify MV^0^ formation beyond these characteristic optical changes, MV^•+^ consumption due to parasitic H_2_ evolution was excluded; head‐space gas chromatography (GC) of this solution after 2 h of irradiation detected no H_2_. Moreover, EPR spectroscopy also supported the formation of MV^0^ (vide infra). As previously reported [28], MV^0^ formation was also confirmed by later addition of fresh MV^2+^ leading to the re‐appearance of the characteristic MV^•+^ signatures *via* comproportionation of MV^2+^ and MV^0^ (Figure S10). In addition, the color of an irradiated solution changed rapidly from yellow to blue and finally to colorless when intentionally brought into contact with air allowing stepwise re‐oxidation of MV^0^ to MV^•+^ and MV^2+^ by O_2_ (see Figure S11 for slow reoxidation). Finally, the absence of any absorbance at *λ* > 800 nm in Figure 1a excludes MV^•+^ dimerization throughout the photocatalytic experiment [13, 51, 52] which is also verified by extending the recorded optical spectra until 1100 nm (Figure S12).

**FIGURE 1:**
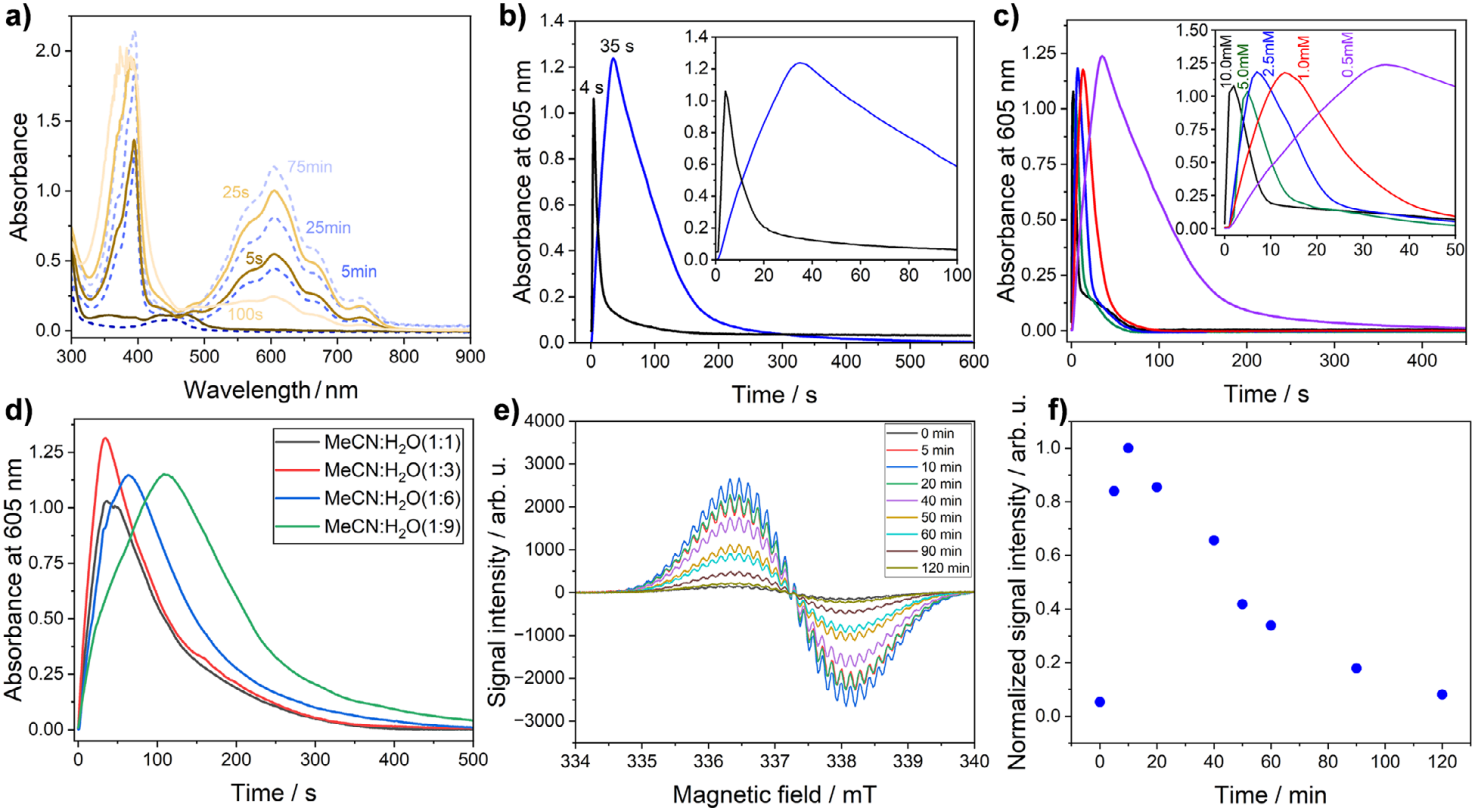
Steady‐state UV/vis absorption spectra of **^CF3^Ru**‐ (yellow solid lines) and **^H^Ru**‐driven (blue dashed lines) MV^2+^ photoreduction in MeCN:H_2_O = 1:1 (v:v) containing 0.12 M TEA and 0.10 M NaH_2_PO_4_; b) ‐ d) MV^2+^ photoreduction kinetics by plotting the characteristic MV^•+^ absorbance at 605 nm versus time; b) for **^CF3^Ru** (black) and **^H^Ru** (blue) in MeCN:H_2_O = 9:1 (v:v) containing 0.5 mM BNAH; the inset highlights the first 100 s; c) for **^H^Ru** in MeCN:H_2_O = 9:1 (v:v) containing different amounts of BNAH; the inset highlights the first 50 s; d) for **^H^Ru** in different MeCN:H_2_O‐mixtures containing 10 mM BNAH. In all cases 100 µM MV^2+^ and 5 µM of **^CF3^Ru** or **^H^Ru** were used. Panel e) shows cw‐EPR spectra during photoreduction of MV^2+^ (200 µM) with **^H^Ru** (5 µM) in pure MeCN and in presence of 2.5 mM BNAH inside the cavity of the EPR spectrometer; panel f) shows the kinetic trace of the EPR signal evolution.

As identical reaction conditions were applied for both complexes and the use of **^H^Ru** only resulted in the accumulation of MV^•+^, a disproportionation‐induced MV^0^ formation in bulk solution for **^CF3^Ru** could be excluded as well as previously described in literature [27]. In the same way, a dark reaction in which MV^0^ is only formed by highly‐reducing TEA^•^ can be excluded too [53]. If the highly reactive [54] TEA^•^, generated by reductive quenching of excited **^CF3^Ru** and subsequent deprotonation, would be the only reductant and not also photoreduced **^CF3^Ru**, the stoichiometric ratio of **^CF3^Ru** to MV^2+^ (1:20 for 100 µM MV^2+^) would preclude > 5% conversion of MV^•+^ to MV^0^. This is due to the fact that in such a scenario reduced **^CF3^Ru** would accumulate in solution, precluding further photooxidation of TEA and thus limiting the overall turnover to one per PS. Consequently, **^CF3^Ru** allowed MV^0^ accumulation *via* two successive eTs by upregulating the reductive quenching pathway [25, 26, 32, 33] at the expense of the overall less efficient oxidative quenching pathway [55, 56]. However, we cannot rule out that TEA^•^ is not contributing to the reduction of MV^2+^ or MV^•+^ at all.

In contrast, literature reports on even stronger oxidizing [Ru(bpz)_3_]^2+^ (bpz = 2,2´‐bipyrazine) complexes to also enable rapid MV^2+^ to MV^•+^ reduction with high QYs but showed no further reactivity toward MV^0^ with different SEDs [34, 35, 36, 37, 38]. The same is true for some derivatives of this complex [46]. We ascribe this to [Ru(bpz)_3_]^2+^ decisively lacking ca. 100 mV of reducing power compared to **^CF3^Ru** [42, 57], while **^CF3^Ru** is still reducing enough (see *E*_red_ in Scheme 1) to enable the follow‐up reduction to access MV^0^ which requires ca. −1.2 V vs. Fc^+^/Fc. A similar balancing of the PS redox potentials has recently been shown to beneficially impact the visible‐light‐induced phosphorylation of tertiary aliphatic amines [58]. The absence of MV^0^ formation using [Ru(bpz)_3_]^2+^ in presence of different SEDs [34, 35, 36, 37, 38] further rules out SED degradation products acting as dominant reductants for MV^•+^.

Based on the amount of utilized **^CF3^Ru**, under these conditions MV^•+^ was generated with a rather constant turnover frequency (TOF) of ca 0.9 s^−1^, followed by MV^0^ production at a ca. fourfold slower rate. This rapid formation of MV^•+^ followed by a slower production of MV^0^, likely caused by competing productive and unproductive eTs with PS* and PS^+^ [28] as detailed above, has previously been reported for self‐assembling Ru(II) polypyridyl complexes as well [25].

In order to identify whether MV^0^ production with **^CF3^Ru** and TEA was a consequence of the photochemical mechanism, that is reductive quenching to access the more reactive photoreduced PS, rather than a specific property of **^CF3^Ru**, 1‐benzyl‐1,4‐dihydronicotinamide (BNAH) was used as easier to oxidize SED (*E*_ox_ = 0.19 V) [43, 44, 59]. In accordance with E*_red_ for **^H^Ru** and **^CF3^Ru** (Scheme 1), luminescence quenching results (Figure S13) proved that BNAH allows reductive quenching for both, **^CF3^Ru** (K_SV_ = 5000 M^−1^) as well as **^H^Ru** (K_SV_ = 87 M^−1^). In line with Schemes S1–S4 as well as Tables S1 and S2 that highlight the need for reductive quenching for MV^0^ formation using typical Ru(II) polypyridyl complexes with E*_ox_ insufficient for oxidative quenching by MV^•+^ [7], BNAH enabled MV^0^ accumulation for **^CF3^Ru** at a ca. 10‐fold faster rate compared to **^H^Ru** (Figure 1b). Although **^H^Ru** does possess an *E*_red_ value being ca. 0.4 V more negative than that of **^CF3^Ru** (see Scheme 1), the observed reaction rate differences are likely ascribed to the different efficiencies of the PSs engaging in reductive quenching with BNAH, as both photoreduced PSs hold enough reduction potential to reduce MV^2+^ a second time. Consequently, when utilizing BNAH as SED, this also indicates that in this case reductive quenching with the SED represents the overall rate‐determining step rather than the subsequent eT onto MV^•+^ for which **^H^Ru** would possess the larger driving force.

As anticipated from a reductive quenching‐dominated photochemical process and as depicted in Figure 1c, rates of formation of MV^•+^ and MV^0^ by **^H^Ru** revealed a BNAH concentration dependence. The signal intensity at 605 nm reached its maximum in MeCN:H_2_O = 9:1 (v:v) after 2.0 s in the presence of 10 mM BNAH which is ca. 2,200 times faster than when phosphate‐buffered TEA was used as SED instead. 2.0 s for complete MV^2+^ to MV^•+^ reduction is equal to a turnover number (TON) of 20 in 2 s and, thus, a TOF of 10 s^−1^. The required time is increased to 5.0 s, 7.0 s, 13 s and 35 s if BNAH concentrations were 5 mM, 2.5 mM, 1.0 mM or 0.5 mM instead. These numbers furthermore correlate with QYs of MV^•+^ formation of ca. 140%, 56%, 40%, 21%, and 8%, respectively. Similarly high QYs exceeding 75% or even unity have been reported in literature using either strongly oxidizing [Ru(bpz)_3_]^2+^‐like PSs [34, 35, 36, 37, 38, 46] and semiconductor particles [47, 60] in the presence of various SEDs as well as **^H^Ru** utilizing the negatively charged SED ethylenediaminetetraacetic acid (EDTA) efficiently suppressing back‐eT after oxidative quenching by MV^2+^ [28]. Numbers exceeding 100% require dark reactions between SED degradation products and MV^2+^ to account for the fact that a purely PS‐driven process can only reach a QY of unity. Nevertheless, in none of these references MV^0^ formation was reported, while recent pump‐pump‐probe experiments with **^H^Ru** in presence of ascorbate undergoing reductive quenching with **^H^Ru** transiently generated MV^0^ [32, 33].

Due to superior reductive quenching of **^CF3^Ru** with BNAH, only with dilute SED (0.5 mM) accurate rate determination (TOF = 5 s^−1^ (QY ca. 70%) vs. 0.6 s^−1^ (QY ca. 8%) in the case of **^H^Ru**) for MV^•+^ formation of was possible (Figure 1b). Exclusion experiments verified that the reaction was light‐dependent (Figure S14) and the background photoreactivity of BNAH with MV^2+^ occurred on a significantly slower time scale compared to the **^CF3^Ru**‐ and **^H^Ru**‐photocatalyzed processes (Figure S15). In accordance with literature [59], using BNAH together with **^CF3^Ru** or **^H^Ru** in a 1:1 or a 0.5:1 ratio vs. MV^2+^ clearly highlighted that BNAH served as two‐electron reductant under these conditions (Figures S16), explaining the high QY exceeding unity discussed above.

Moreover, as shown in Figure 1, panels e) and f), the use of BNAH as SED and **^H^Ru** as PS allowed to work in pure MeCN enabling the use of EPR spectroscopy to further support the formation of MV^0^ at the end of the photocatalytic process. Similar to the corresponding optical spectroscopy data in pure MeCN (Figure S17) the EPR signal associated with MV^•+^ is rapidly increasing before decreasing at a slower rate.

Considering the redox potentials given in Scheme 1, using TEA 1.13 eV (42%) of photonic energy of 2.68 eV (*λ* = 463 nm) in the first step and 1.52 eV (57%) in the second step, that is 2.65 eV (49%) in total, were stored. For BNAH these numbers are 1.01 eV (38%) and 1.40 eV (52%) or 2.41 eV (45%) in total. Furthermore, as shown in Figure 1d, a solvent‐dependence for MV^2+^ photoreduction by **^H^Ru** was observed using BNAH as SED. The lower the amount of H_2_O in the utilized MeCN/H_2_O‐mixtures the faster the rates of MV^•+^ and MV^0^ formation. The same effect was also observed with **^CF3^Ru** using BNAH or TEA as SEDs (Figures S18 and S19) being in accordance with previous results on DMF/H_2_O‐mixtures also demonstrating an improved performance in solvent mixtures with a high organic share [27].

In contrast to the SED concentration dependence and in accordance with a reductive quenching‐dominated photocatalytic process, the rate of MV^•+^ accumulation proved to be virtually independent of MV^2+^ concentration. When varying c(MV^2+^) from 50 µM to 100 µM and 200 µM using **^CF3^Ru** in combination with phosphate‐buffered TEA, the time required to reach full onefold reduction of MV^2+^ could be modelled with a reaction order of zero for MV^2+^ when considering MV^•+^ as co‐absorbing species for photons of *λ*_exc_ = 463 nm (see Supporting Information section 4). In contrast, the decreased rate and nonlinear behavior during subsequent MV^0^ formation indicates kinetic competition between MV^•+^/MV^0^ and TEA for reductive quenching of PS* or reduction of PS^+^ [28]. These results suggested reductive quenching of PS* by the SED to be the rate‐determining step rather than diffusional collisions between PS^−^ and the acceptor. Therefore, a recently reported viologen‐containing co‐polymer was used [61], containing solubilizing benzyl trimethylammonium groups in addition to viologen units. As anticipated, the rate of one‐ and twofold viologen reduction with **^CF3^Ru** using TEA as SED—despite the restricted movement of the polymer‐attached viologens—was not different to molecular MV^2+^ (23 s for mono‐reduction of MV^2+^ and 19 s for the polymer‐attached viologens, see Figures S20 and S21). Interestingly and although the high local concentration of viologens in the polymeric system lead to an additional MV^•+^ stabilization via dimerization [13, 51, 52, 62], further reduction to MV^0^ was not impeded.

In order to understand the photochemically rarely‐observed secondary reduction of MV^•+^ to MV^0^ (*E*_red_ = −1.21 V vs. Fc^+^/Fc), AIMD were utilized [7]. Based on the experimental results showing reductive quenching as prerequisite for accessing MV^0^ via formation of more reactive PS^−^, this process is described *via* the interaction between mono‐reduced ruthenium complexes (**^CF3^Ru^+^** or **^H^Ru^+^**, generated *via* reductive quenching with a SED) and MV^•+^. In summary, the driving force for the forward reaction, namely **^CF3/H^Ru^+^** + MV^•+^ → **^CF3/H^Ru^2+^** + MV^0^ (Figure 2), can be estimated *via* comparison between the energies of a dimer system, comprised of one Ru complex and one MV, in different multiplicities. In the optimized geometry of the Ru/MV dimer, with a net charge of +2, the closed‐shell (singlet) configuration corresponds to the product states (**^CF3/H^Ru^2+^** + MV^0^), as the doubly occupied HOMO is localized on the MV (Table S3). Atomic dipole moment corrected Hirshfeld (ADCH) analysis reveals that the majority of the net +2 charge of this product states is localized on the Ru complex, +1.78 and +1.59 for **^H^Ru** and **^CF3^Ru**, respectively (Table S4). Contrastingly, in the same geometry but with triplet multiplicity, the (open‐shell) system corresponds to the reactant states, (**^CF3/H^Ru^+^** + MV^•+^) with the spin density plots showing an unpaired electron on the MV as well as on the Ru complex (Table S4), that is both being singly reduced species. Accordingly, the ADCH charges correspond to approximately +1 for the Ru complex and MV each (Table S4). Consequently, the single point energy difference between the singlet (*E*_S_) and triplet (*E*_T_) configurations (∆*E*_ST_ = *E*_S_—*E*_T_) of the **^H^Ru**/MV and **^CF3^Ru**/MV dimers in a fixed geometry provides a means by which to estimate the thermodynamic favorability for the forward eT and MV^0^ formation. A detailed description of the computational methodology can be found in the Supporting Information.

**FIGURE 2:**
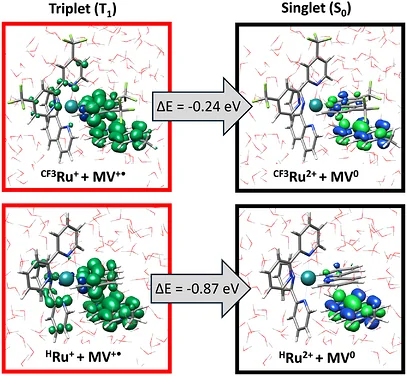
Illustration of the reactant (left) and product (right) states for the secondary reduction of MV^•+^ to MV^0^ by **^CF3^Ru** (top) and **^H^Ru** (bottom) represented by triplet (T_1_) and singlet (S_0_) multiplicities, respectively. The spin density of the triplet state (left) shows unpaired electrons on both, **^CF3^Ru** or **^H^Ru** and the MV moieties, thus representing the reactant state (**^CF3/H^Ru^+^** + MV^•+^). The HOMO of the singlet configuration (right) primarily resides on the MV moiety, thereby representing the product state (**^CF3/H^Ru^2+^** + MV^0^). The energy difference ∆*E*_ST_ = *E*_S0_ – *E*_T1_ corresponds to the energy of the product (singlet) state minus that of the reactant (product) state taken from the global minimum geometry in an explicit H_2_O environment.

In their corresponding globally optimized S_0_ geometries within an implicit H_2_O field, the MV is localized beneath a bpy ligand (Table S4) and ∆*E*_ST_ for the **^CF3^Ru**/MV and **^H^Ru**/MV dimers is −0.25  and −0.61 eV, respectively. This negative ∆*E*_ST_ shows that the singlet energy, that is that representing the product state is lower in energy than the triplet reactant state **^CF3/H^Ru^+^** + MV^•+^, therefore the formation of MV^0^ is thermodynamically favored. In an implicit MeCN solvent field, almost identical energies were obtained (Table S4). Furthermore, by inclusion of explicit solvent (H_2_O or MeCN) a similar behavior was observed, with favorable ∆*E*_ST_ of −0.24 and −0.87 eV for the **^CF3^Ru**/MV and **^H^Ru**/MV dimers, respectively (obtained from the global minima geometries in an explicit H_2_O environment, Table S5), which reflect experimentally obtained reduction potentials of **^CF3^Ru** (*E*_red_ = −1.29 V), **^H^Ru** (*E*_red_ = −1.73 V), and MV^•+^ (*E*_red_ = −1.21 V, all potentials vs. Fc^+^/Fc) [7, 42]. However, outside of the optimized S_0_ geometry, ∆*E*_ST_ varies significantly. Thus, the thermodynamics as well as the kinetics of the redox reaction are strongly depending on the local geometry of both encounter complexes.

To model different interactions between the **^CF3/H^Ru**/MV dimers in a range of encounter complex configurations to better reflect experimental conditions, AIMD simulations were performed. The initial geometry at *t* = 0 fs corresponds to the starting DFT optimized global minimum geometry, which reflects the combination of a nonreduced Ru complex and MV^0^. Coordinates obtained at 500 fs time intervals were used to present a variety of plausible interaction geometries between the Ru complex and MV. As with the optimized S_0_ geometry, single point DFT calculations were performed and *E*_S_ and *E*_T_ were compared. As shown in Figure 3a, for the **^CF3^Ru**/MV dimer in an implicit H_2_O solvent field *E*_T_ (reactant state) is lower than *E*_S_ (product state), in all sampled configurations other than the starting geometry, with an average ∆*E*_ST_ (for sampled geometries where *t *> 0 fs) of +0.41 eV, implying that the reduction process is not thermodynamically favorable highlighting the EWG‐based stabilization of the **^CF3^Ru**/MV triplet state. In contrast, but in line with the increased reduction potential of **^H^Ru**, for the **^H^Ru**/MV dimer (Figure 3b) the average ∆*E*_ST_ remains negative at −0.20 eV. Very similar behavior was observed for AIMD simulations performed in an implicit MeCN solvent field (Figure S23).

**FIGURE 3:**
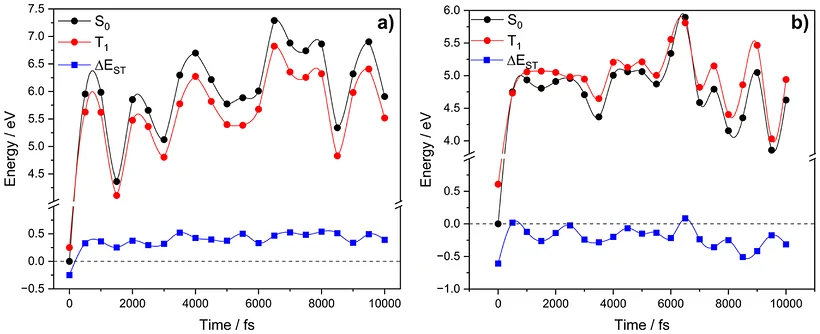
Singlet (black; **^CF3/H^Ru^2+^** + MV^0^) and triplet (red; **^CF3/H^Ru^+^** + MV^•+^) single point DFT energies obtained for the a) **^CF3^Ru**/MV and b) **^H^Ru**/MV dimers in an implicit H_2_O solvent field. The geometry snapshots are obtained at 500 fs intervals from the MD simulations, starting from the DFT optimized encounter complex at *t* = 0. The blue line corresponds to ∆*E*_ST_ = *E*_S_—*E*_T_. When ∆*E*_ST_ < 0 the forward reaction process for the reduction of MV^•+^ to MV^0^ is thermodynamically favored.

To explore the influence of the surrounding solvent in more detail, AIMD simulations were also performed with explicit H_2_O and MeCN (Figures S24 and S25). A similar trend was observed, but with a smaller average ∆*E*_ST_ of +0.19 eV obtained for the **^CF3^Ru**/MV dimer. The solvent environment may therefore play a role in reducing the offset between the state energies. Additionally, the geometry sampled in H_2_O at 2500 fs has ∆*E*_ST_ of −0.14 eV, demonstrating that such energetically favorable configurations are obtainable. In accordance with the experimentally obtained reduction potentials, in both the **^CF3^Ru**/MV and **^H^Ru**/MV dimers the lowest energy configuration obtained (global minima) favor the forward reaction. However outside of this specific encounter complex configuration, an energetic configuration which favors the forward reaction to reduce MV^•+^ to MV^0^ is less likely to be found for **^CF3^Ru**/MV.

The computational data, along with the observed concentration independence of the overall reaction rate with respect to MV^2+^, thus indicate that the kinetics of the initial photochemical step accessing PS^−^ by reductive quenching, rather than the subsequent chemical electron transfer step between PS^−^ and MV^•+^, dictate the rate of MV^0^ generation. As discussed above, otherwise **^H^Ru** should have outperformed **^CF3^Ru** using BNAH as SED. Additionally, it should be noted that even for the herein reported fastest process for **^CF3^Ru** with a TOF of ca. 10 s^−1^ (initial rate of MV^0^ formation in presence of 10 mM BNAH, see Figure S14), on average the system is given ca. 100 ms of time to collide in the correct geometry. This is more than enough time when considering *k*_q_ ≈ 10^9^ M^−1^ s^−1^ and c (MV^•+^) = 100 µM yielding a collision rate between both species of 10^5^ s^−1^. However, the small number of correct geometries in case of **^CF3^Ru**/MV for which forward eT to access MV^0^ is favorable, highlights, that due to the underlying thermodynamics **^CF3^Ru** might already represent a limiting case for an oxidizing PS capable of forming MV^0^. As discussed above, the only ca. 100 mV less reducing [Ru(bpz)_3_]^2+^ was already incapable of forming MV^0^ [34, 35, 36, 37, 38, 46]. The obvious need of overall well‐balanced redox potentials in the ground‐ as well as the excited states for a suitable PS to function likely explains the scarce literature on light‐driven MV^0^ formation even despite MV^2+^ being an acceptor and electron relay used in numerous works and applied for decades.

## 3 Conclusion

To summarize, in this study, we reported a thermodynamically rationalized approach to achieve efficient twofold MV^2+^ reduction. Switching the photochemical mechanism to reductive quenching and by that accessing the photoreduced PS, representing the most reducing of all intermediates, is the key to an efficient process. Similarly, also in various organic photoredoxcatalytic processes and artificial photosynthetic systems reductive quenching of PS* is necessary to unlock the desired reactivity [63, 64, 65]. The different combinations of SEDs and structurally simple photoredox active Ru(II) polypyridyl complexes exemplify its future application potential. In detail, using a reductive quenching regime full photocatalytic conversion from MV^2+^ to MV^•+^ proceeds in seconds compared to tens of minutes or hours for an oxidative quenching regime. The process can additionally be accelerated by increasing the amount of organic solvent in MeCN/H_2_O mixtures. Theoretical insights into the final eT step highlight the need for geometrically ideal arrangements for **^CF3^Ru**/MV and substantiate the assessment that delicate balancing of all relevant potentials is key to unlock efficient two‐electron reduction of MV^2+^ (Schemes S3 and S4; Tables S1 and S2). In view of the broad application potential of viologens as electron relays in artificial photosynthetic systems, the herein reported modular approach could significantly improve the photonic efficiencies of such processes. Additionally, application of the basic considerations underlying these findings could also improve photocatalytic reactions requiring accumulation of several uncompensated charges on one specific site such as in, for example, oligonuclear photoredox catalysts [66]. Moreover, the replacement of the herein utilized SEDs by reversible ones, that is electron mediators, could even pave the way to more sustainable systems [67].

## Conflicts of Interest

The authors declare no conflicts of interest.

## Supporting information



The authors have cited additional references within the Supporting Information [68, 69, 70, 71, 72, 73, 74, 75, 76, 77, 78, 79, 80, 81].
**Supporting File 1**: chem71467‐sup‐0001‐SuppMat.pdf.

## Data Availability

The data that supports the findings of this study are available in the Supporting Information of this article.
