# Supplementary material for: Efficient Light‐Driven Multi‐Electron Accumulation on Viologens by Molecular Control Over Photochemical Reaction Pathways
Source: Chemistry. 2026 Jul 30;32(40):e71467. doi: 10.1002/chem.71467 (PMC13716007; doi:10.1002/chem.71467)
Supplement: Supplementary file 1 [file CHEM-32-e71467-s001.pdf]

Supporting Information

Title: Efficient Light-Driven Multi-Electron Accumulation on Viologens by Molecular Control over Photochemical Reaction Pathways

Authors: Fatemeh Sousani,[a] Georgina E. Shillito,[b] Alexander K. Mengele,[a] Martin D. Hager,[c,d,e,f,g] Ulrich S. Schubert,[c,d,e,f,g] Stefanie Gräfe,[b,h] Stephan Kupfer,[b] Sven Rau[a,]*

Affiliations:

[a]Institute of Inorganic Chemistry I, Ulm University, Albert-Einstein-Allee 11, 89081 Ulm, Germany.

[b]Institute of Physical Chemistry and Abbe Center of Photonics, Friedrich-Schiller University, Helmholtzweg 4, 07743 Jena, Germany.

[c]Laboratory of Organic and Macromolecular Chemistry (IOMC), Friedrich Schiller University Jena, Humboldtstraße 10, 07743 Jena, Germany.

[d]Jena Center for Soft Matter (JCSM), Friedrich Schiller University Jena, Philosophenweg 7, 07743 Jena, Germany.

[e]Center for Energy and Environmental Chemistry Jena (CEEC Jena), Friedrich Schiller University Jena, Philosophenweg 7a, 07743 Jena, Germany.

[f]Helmholtz Institute for Polymers in Energy Applications Jena (HIPOLE Jena), Lessingstraße 12-14, 07743 Jena, Germany.

[g]Helmholtz Zentrum für Materialien und Energie Berlin (HZB), Hahn-Meitner-Platz 1, 14109 Berlin, Germany.

[h]Fraunhofer Institute for Applied Optics and Precision Engineering, Albert-Einstein-Str. 7, 07745 Jena, Germany.

Table of contents:

1. Experimental details 2

2. Quantum yield calculations 9

3. Supporting Figures (photocatalysis, mechanistic insights, structural characterization) 11

4. Kinetic model 22

5. Theory part 24
   5.1. Computational details 24
   5.2. Initial optimization 24
   5.3. Explicit solvation 24
   5.4. Molecular Dynamics simulations 24
   5.5. Method utilized to model the second reduction process 25

6. References 31

1. Experimental details

Unless otherwise noted all solvents and reagents were obtained from commercial suppliers and used without purification. 1-Benzyl-1,4-dihydronicotinamide (BNAH) was synthesized according to literature[1] and was stored as yellow solid under light exclusion, under argon and at -20°C in a freezer. [(fbpy)$_2$RuCl$_2$] was synthesized according to literature as well.[2] Methyl viologen, MV^{2+}, was used as chloride salt throughout all experiments. The viologen-containing polymer was synthesized according to literature as well.[3]

NMR spectra were recorded on a Bruker Avance Neo 400 MHz spectrometer at 298 K. ^{1}H NMR and ^{13}C NMR shifts are referenced according to the (in the case of ^{1}H NMR spectra) residual signal of non-fully deuterated solvent or (in the case of ^{13}C NMR spectra) deuterated solvent as internal standard. Coupling constants J are presented as absolute values in Hz. Chemical shift values (δ) are given in parts per million (ppm) using residual solvent signals, specifically to 1.94 ppm (quintet, ^{1}H NMR spectra) or 118.26 ppm (singlet, ^{13}C NMR spectra) for CD_3CN. For spectra evaluation MestReNova (version: 12.0.0-20080) was used. For the characterization of the NMR signals the following abbreviations are used: s = singlet, d = doublet, q = quartet, m = multiplet and dd = doublet of doublets. For better readability, phase correction (automatic) as well as baseline correction (Whittaker smoother) were applied.

High resolution mass spectrometry (HRMS) was performed using a Fourier Transform Ion Cyclotron Resonance (FT-ICR) mass spectrometer solariX (Bruker Daltonics) equipped with a 7.0 T superconducting magnet and interfaced to an Apollo II Dual ESI/MALDI source. For all measurements trans-2-[3-(4-tert-Butylphenyl)-2-methyl-2-propenylidene]malononitrile (DCTB) was used as matrix. Spectra were analyzed with the DataAnalysisViewer 4.2 from Bruker. Spectra simulation was performed with mMass Version 5.5.0.

In cases where no photocatalytic processes were optically tracked, ground-state **UV-vis absorption spectroscopy** was performed on a JASCO V-670 UV-vis-NIR Spectrophotometer using gas-tight quartz glass cuvettes (d = 10.0 mm, Starna Scientific). Steady-state **emission spectroscopy** was performed on a JASCO FP-8500 Fluorescence Spectrometer with gas-tight quartz glass cuvettes (d = 10.0 mm, Starna Scientific).

For **rapid UV-vis absorption spectroscopy** following photocatalytic processes, measurements were performed on an Avantes AvaSpec-ULS2048CL detector unit coupled with an AVA AvaLight-DH-S-BAL light source. Light source, cuvette holder and detector were connected with fiberoptic cables. Measurements were performed in gas-tight screw-capped quartz glass cuvettes (d = 10.0 mm, Starna Scientific) inside an argon-filed glovebox (GB). Spectra were measured in different time intervals, as indicated (typically 1 s intervals), depending on the rate of methyl viologen (MV^{2+}) reduction to $MV^{\bullet+}$ or MV^0. Irradiation was performed with a custom-made LED stick ($\lambda_{emi,max}$ = 463 nm (see Figure S7); P_{out} = 45 mW/cm^2) installed parallel to the cuvette at a distance of 1 cm, under constant air-cooling by two fans to ensure that the sample was not heated during measurement. The cuvette itself was placed

into a fixed cuvette holder (see Figure S26). For details on the determination of the photonic power output of the LED stick and quantum yield calculations, see chapter 2 of the Supporting Information.

cw-EPR-spectroscopy was performed on a MiniScope MS5000 Bench-Top EPR from Bruker (MagnetTech – Freiberg Instruments) equipped with a TCH04 (Module E1104) temperature controller using 50 µL glass capillaries as sample compartments. The capillaries were sealed inside a glovebox using a Capillary Tube Sealer by Cha-Seal and proofed to be air-tight over the time course of the experiment. The microwave power was swept in the beginning of an experimental series to get an optimal signal but then kept constant during all measurements.

For the photoreduction of MV^{2+}, 5 µM ^{H}**Ru**, 2.5 mM BNAH and 200 µM MV^{2+} were dissolved in pure MeCN in an argon-filled GB. The capillaries were then transported to the spectrometer in darkness. A 470 nm blue light source LED (1 W energy input, ± 25 nm at FWHM) custom-mounted onto the spectrometer was used to enable the photoreactions. This LED allowed to continuously irradiate into the cavity of the spectrometer. The sweep time was adjusted prior to measurement so a good signal was observed. Settings for the EPR measurements (was used if not otherwise noted): Microwave power: 10 mW, modulation amplitude: 0.2 mT, modulation frequency: 100 kHz.

The amount of generated hydrogen was determined by **gas chromatography (GC)** on a Shimadzu GC-2030 with a barrier ionization discharge (BID-2030) detector and helium as carrier gas (column: Restek SH-Rt-Msieve 5A, ID: 0.32 mm; film thickness: 30 [micro]m, length: 30 m, oven temp. 80 °C, [check current method to be sure]) using 100 µL of the gas phase. The GC was calibrated by injection of different volumes of a test gas mixture containing known percentages of hydrogen.

Typical procedure for preparation of photocatalytic MV^{2+} reduction experiments:

Photocatalysis experiments were carried out utilizing one of two ruthenium-based photosensitizers (PSs), named ^{H}**Ru**, i.e., $[Ru(bpy)_3](PF_6)_2$, or ^{CF3}**Ru**, i.e., $[(fbpy)_2Ru(phen)](PF_6)_2$. (bpy = 2,2´-bipyridine, fbpy = 4,4'-di-trifluoromethyl-2,2'-bipyridine, phen = 1,10-phenanthroline). Stock solutions of the two complexes were each made in dichloromethane (DCM) and acetonitrile (MeCN), respectively. A stock solution of methyl viologen dichloride ($MVCl_2$, herein throughout abbreviated as MV^{2+}) in MeOH was also prepared. Triethylamine (TEA) and BNAH of different concentrations were used as electron donors. For TEA-based samples, no pure TEA but an aqueous TEA-NaH_2PO_4 buffer (in a 1.2:1 concentration-based ratio) was prepared inside the GB and used to prevent MV^{2+} degradation under basic conditions. The experiments were conducted with different solvent (MeCN:H_2O) ratios, as indicated.

Samples were first prepared in 4.5 mL GC vials outside the GB by mixing appropriate volumes of stock solutions of Ru complex and MV^{2+}. Subsequently, the solvents were evaporated by heating the vials at 60 °C. The dried residues were next moved into an argon-filled GB. For BNAH-containing samples, BNAH was added as solid to the samples after they cooled to room temperature and before the transfer into the GB. Inside the GB, the appropriate volumes of MeCN and water were added (total volume of 3 mL). For TEA-containing samples, MeCN and water were added first, followed by an aqueous TEA-NaH_2PO_4 buffer solution of suitable concentration. After gentle mixing, samples were

transferred into screw-capped quartz glass cuvettes. For photocatalysis experiments, the sealed cuvettes were kept inside the GB and positioned in a fixed cuvette holder with a small fan for cooling and equipped with an custom-made LED array ($\lambda_{emi,max}$ = 463 nm; FWHM = 20 nm; P_{out} = 45 mW/cm^2) for irradiation, see Figure S26.

Typical procedure for luminescence quenching experiments:

All experiments were performed under argon atmosphere. Samples were prepared inside an argon-filled GB. Before any addition of the quencher solution, the volume of the sample solution was V = 3 mL.

Luminescence quenching experiments using MV^{2+} and TEA as quenchers were performed as follows: For each experiment, two solutions were prepared. One contained 3 mL of ^{H}**Ru** or ^{CF3}**Ru** (5 µM) in a MeCN/water mixture (1:1, v/v) inside a 1 cm x 1 cm cuvette. The second vial contained the same Ru complex (5 µM) along with the quencher (10 mM MV^{2+} or 10 mM TEA) in the same solvent system. Multiple aliquots of 50 µL from the ^{H}**Ru** or ^{CF3}**Ru** + quencher solution were added step-wise to the vial containing only the ^{H}**Ru** or ^{CF3}**Ru** complex. After each addition, the emission spectrum was recorded. Emission intensities (I) were normalized to the initial emission intensity (I_0), and the resulting I_0/I values were used to construct Stern–Volmer plots.

Quenching experiments with BNAH were performed as follows: Two solutions were prepared in MeCN/H_2O (1:1, v/v), one containing the 3 mL of ^{H}**Ru** or ^{CF3}**Ru** complex (5 µM) and another containing the same ^{H}**Ru** or ^{CF3}**Ru** complex concentration (5 µM) with BNAH (10 mM). Inside the GB, cuvettes were filled with the Ru-only solution, and defined volumes (0 µL, 158 µL, 333 µL, 529 µL, 750 µL, and 1000 µL) of the ^{H}**Ru** or ^{CF3}**Ru** + BNAH solution were added to obtain final BNAH concentrations of 0, 0.5 mM, 1.0 mM, 1.5 mM, 2 mM and 2.5 mM inside the cuvette, respectively. After each addition, the cuvettes were sealed and emission spectra were recorded. Emission intensities were normalized to the initial intensity (I_0), and Stern–Volmer plots were generated by plotting I_0/I against the BNAH concentration.

*Synthesis of [(fbpy)$_2$Ru(phen)](PF$_6$)$_2$ (^{CF3}**Ru**)*

60 mg (0.064 mmol, 1.0 eq.) of [(fbpy)$_2$RuCl$_2$] and 60 mg (0.33 mmol, 5.2 eq) of 1,10-phenanthroline were mixed in a solvent mixture of 60 mL ethanol and 30 mL distilled water. The mixture was refluxed at 120 °C for 24 h. A red-colored solution was obtained, from which ethanol was evaporated via rotary evaporation. Subsequently, 720 mg of NH_4PF_6 was dissolved in 30 mL of water and slowly added to the remaining solution to induce precipitation. The resulting precipitate was collected utilizing vacuum filtration with a glass frit. The solid was then washed several times with water and diethyl ether. The remaining powder was collected and dissolved in MeCN, and finally dried once more by rotary evaporation to isolate crude [(fbpy)$_2$Ru(phen)](PF$_6$)$_2$ (^{CF3}**Ru**) complex. For further purification, the crude product was subjected to Sephadex column chromatography using a CAM solvent system ($CHCl_3$:acetone:MeOH = 47:30:23, v:v:v) to remove residual impurities and yielding 45 mg (0.039 mmol, 62 %) the pure ^{CF3}**Ru** complex. The obtained NMR data are in accordance with those recently obtained for the Cl-salt of this complex.[4]

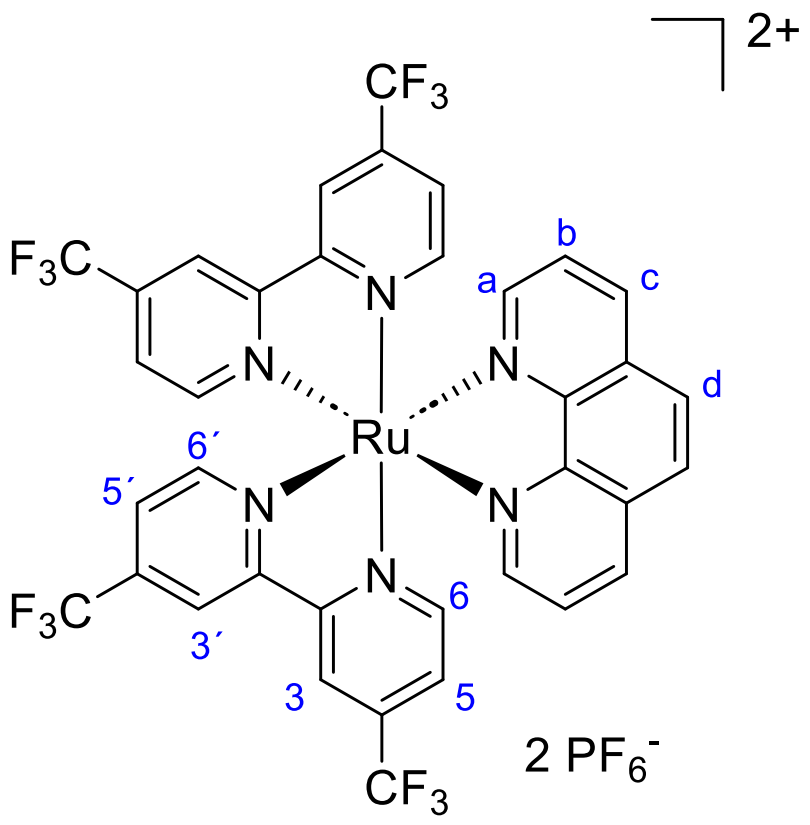

[(fbpy)$_2$Ru(phen)](PF$_6$)$_2$

^{CF3}**Ru**

^{1}H NMR (400 MHz, CD_3CN) δ 8.97 (s, 2H, H_3), 8.94 (s, 2H, $H_{3'}$), 8.69 (dd, J = 8.3, 1.3 Hz, 2H, H_a), 8.28 (s, 2H, H_d), 8.12 (d, J = 5.9 Hz, 2H, H_6), 8.06 (dd, J = 5.2, 1.2 Hz, 2H, H_c), 7.80 – 7.75 (m, 4H, $H_{6',b}$), 7.74 (dd, J = 6.0, 1.4 Hz, 2H, H_5), 7.51 (dd, J = 5.9, 1.4 Hz, 2H, $H_{5'}$).

^{13}C NMR (101 MHz, CD_3CN) δ 158.94, 158.63, 155.39, 154.79, 154.04, 147.93, 139.84 (q, $^2J_{CF}$ = 35.5 Hz), 139.66 (q, $^2J_{CF}$ = 35.4 Hz), 138.97, 132.33, 129.24, 127.35, 124.87 (m, 2 signals), 123.43 (q, $^1J_{CF}$ = 273.1 Hz), 123.33 (q, $^1J_{CF}$ = 273.0 Hz), 122.52 (m, 2 signals).

^{19}F NMR (376 MHz, CD_3CN) δ -65.21, -65.32, -72.95 (d, $^1J_{FP}$ = 706.3 Hz).

HRMS (MALDI-FT-ICR, [M] = $C_{36}H_{20}F_{24}N_6P_2Ru$): calcd. for $[M-2PF_6]^+$ 866.0611; found 866.0614; calcd. for $[M-PF_6]^+$ 1011.0253; found 1011.0265; calcd. for $[2M-PF_6]^+$ 2167.0159; found 2167.0169; calcd. for $[3M-PF_6]^+$ 3323.0144; found 3322.9970.

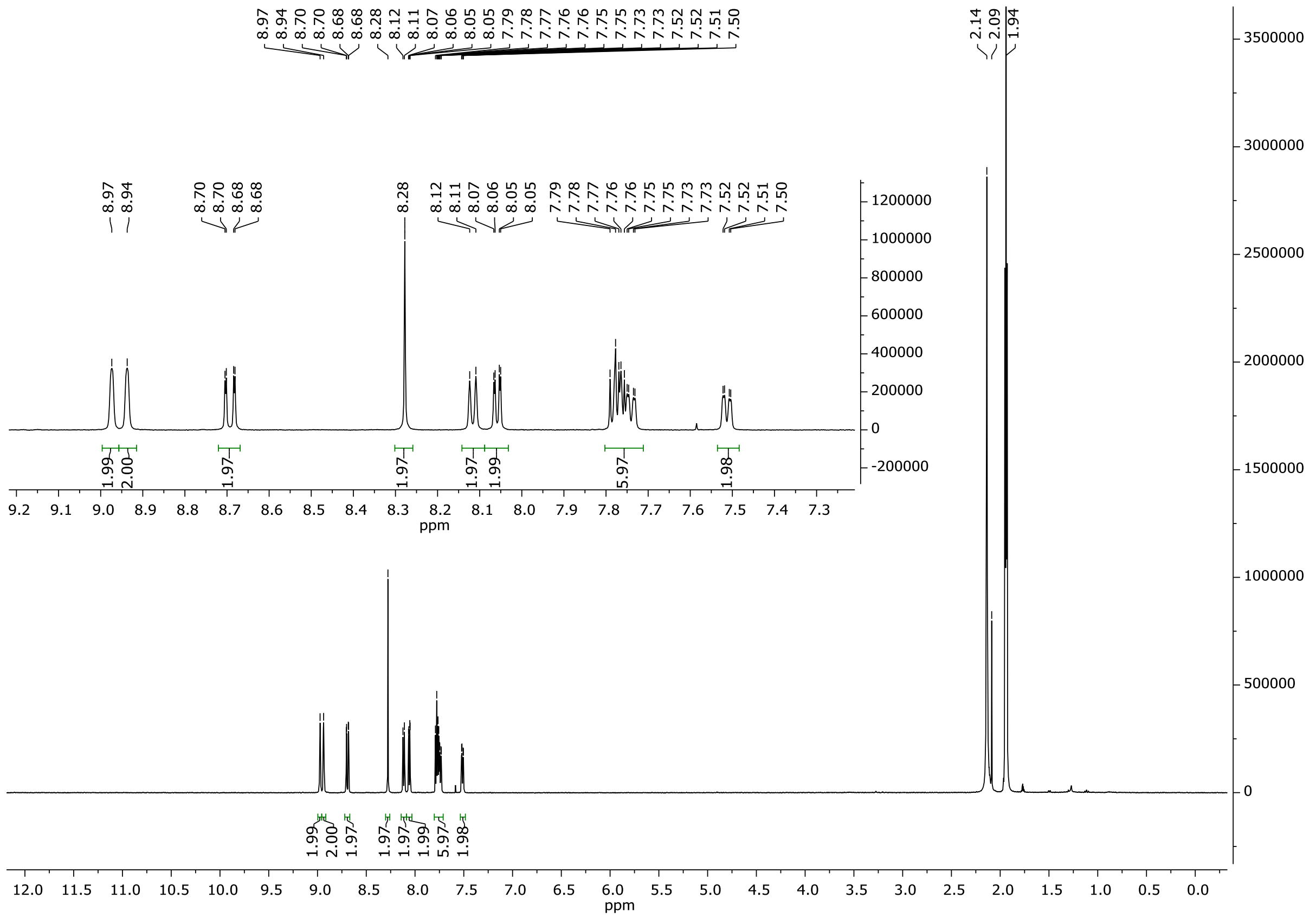

Figure S1. ^{1}H NMR spectrum of ^{CF3}**Ru** in CD_3CN at r.t.

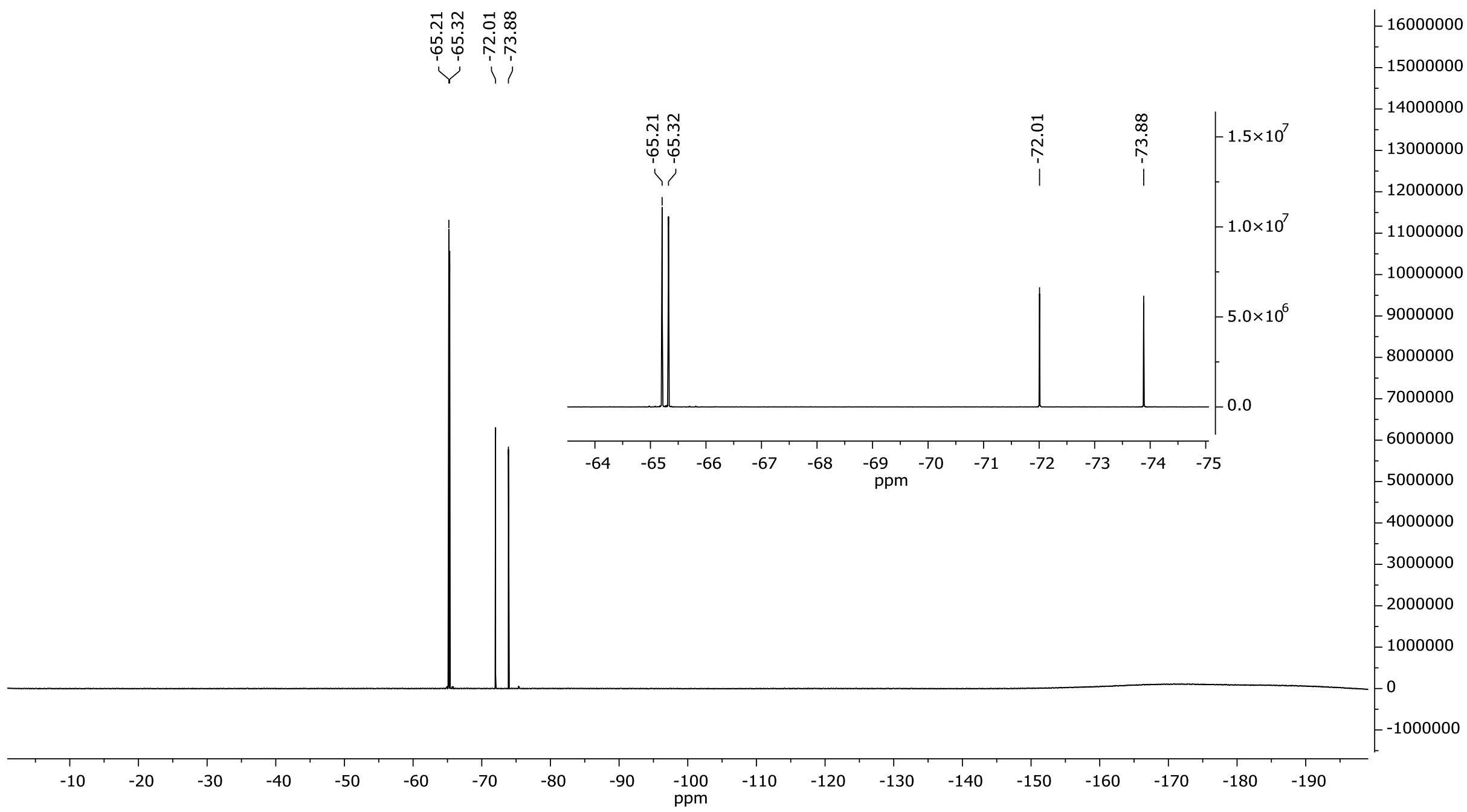

Figure S2. ^{19}F NMR spectrum of ^{CF3}**Ru** in CD_3CN at r.t.

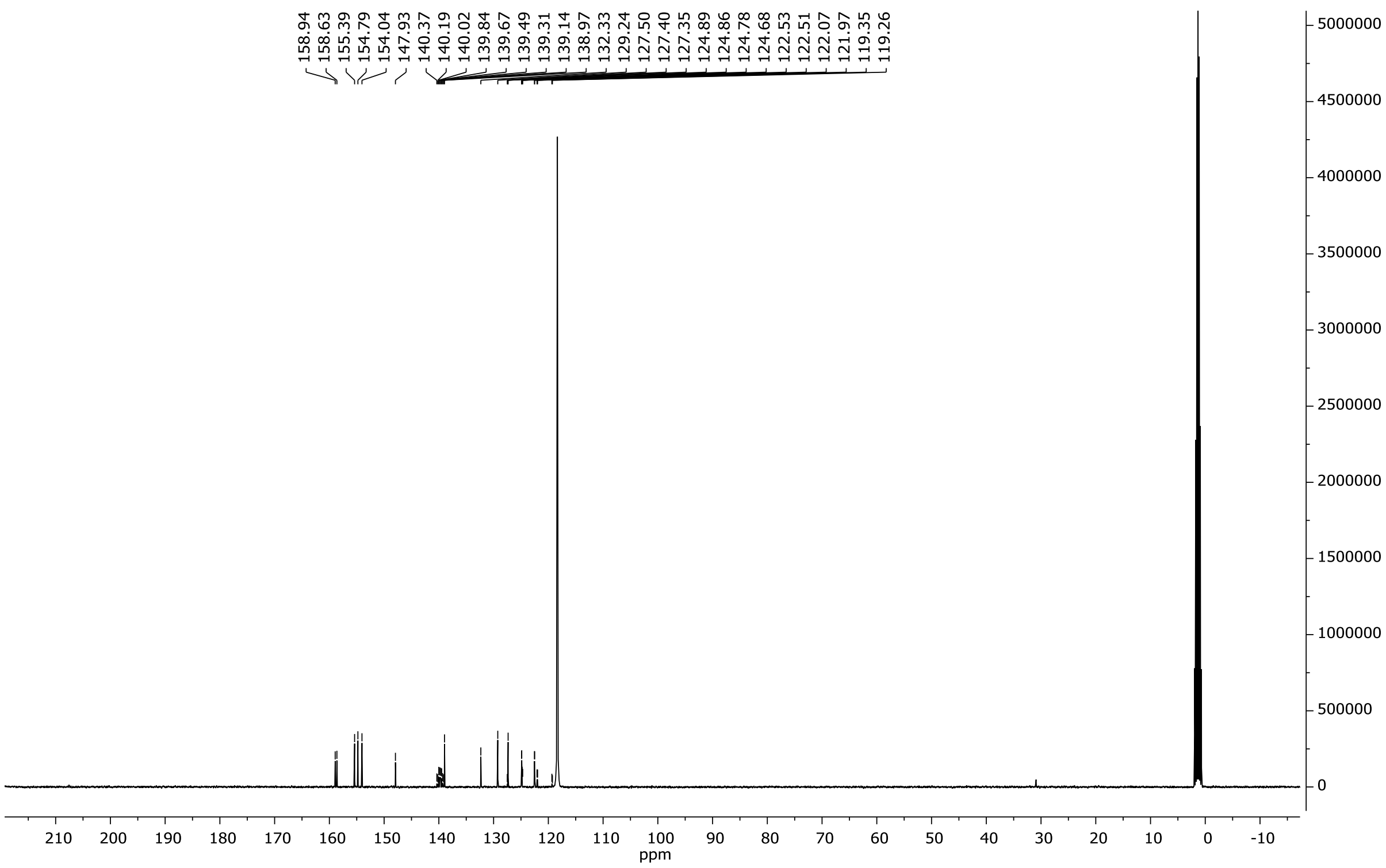

Figure S3. $^{13}C\{^{1}H\}$ NMR spectrum of **^{CF3}Ru** in CD_3CN at r.t. (full view).

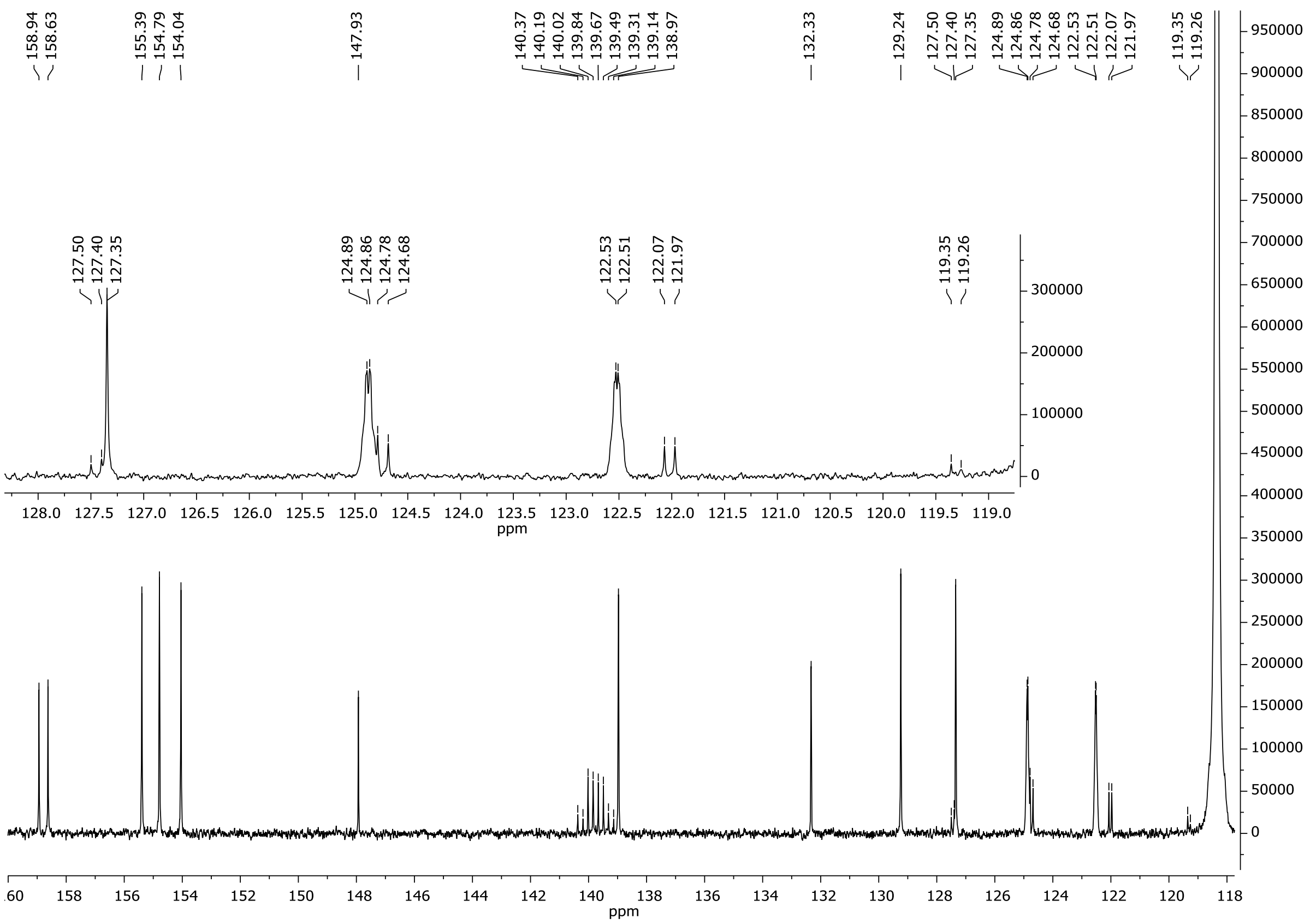

Figure S4. $^{13}C\{^{1}H\}$ NMR spectrum of **^{CF3}Ru** in CD_3CN at r.t. (aromatic region).

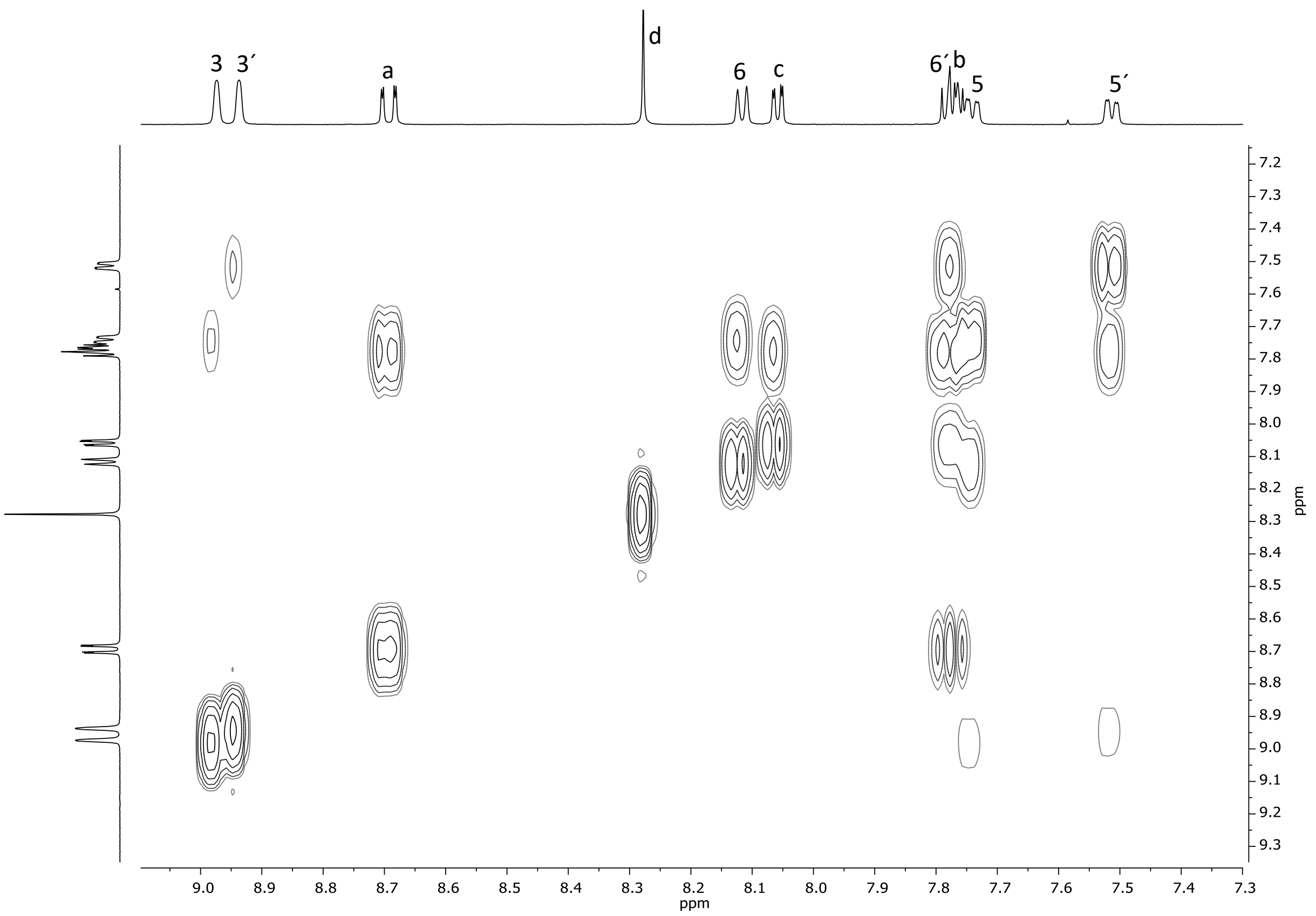

Figure S5. HH-COSY spectrum of ^{CF3}**Ru** in CD_3CN at r.t.

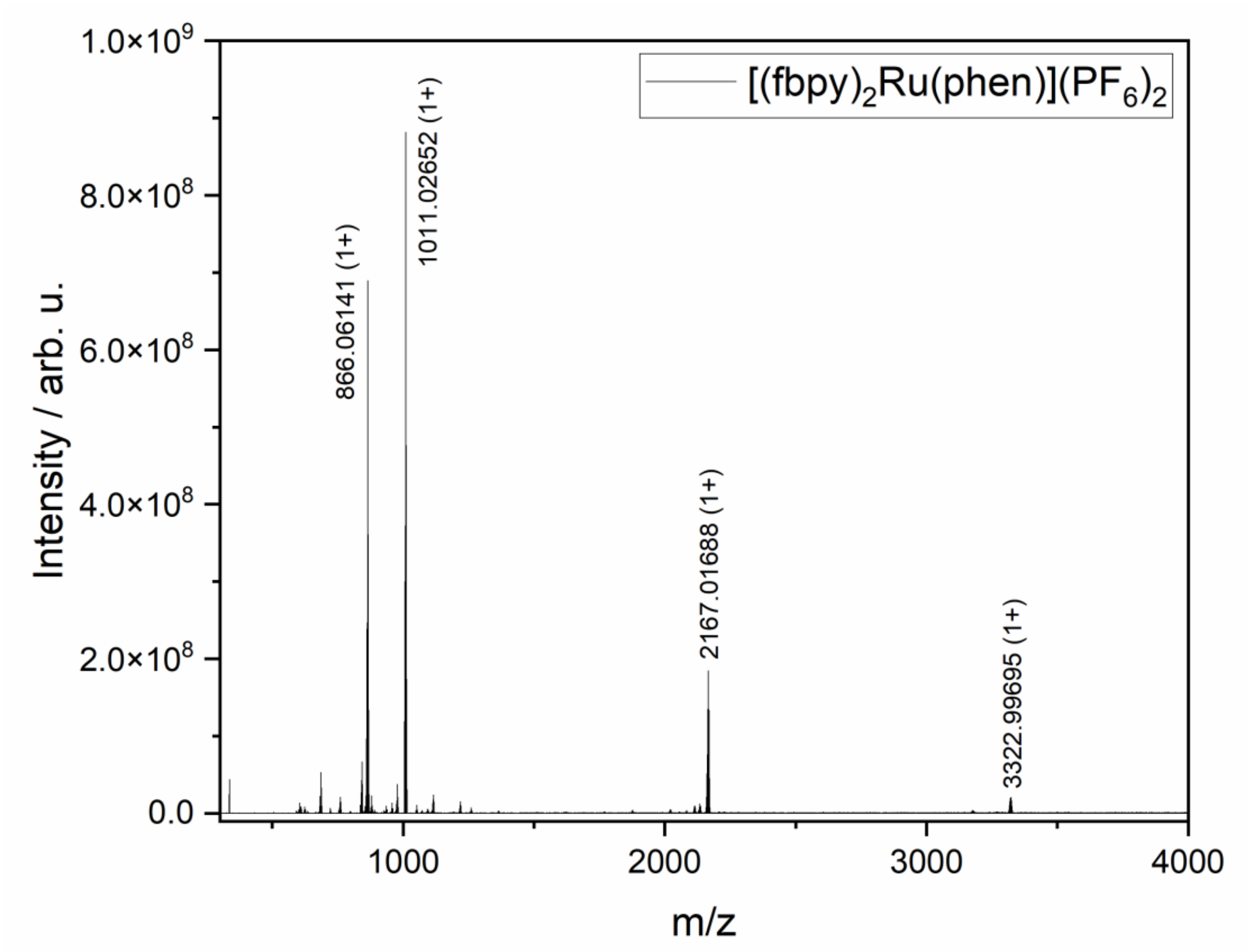

Figure S6. MALDI mass spectrum of ^{CF3}**Ru**.

2. Quantum yield calculations

The custom-made LED-stick is equipped with 27 individual blue light emitting diodes (λ_{max} = 463 nm, FWHM = 20 nm, see Figure S7) from NICHIA (type: NSPB500AS). The individual LEDs have a center-to-center distance of 0.7 cm and are aligned in two antiparallel rows. Utilizing a HIOKI 9742 optical sensor connected to a HIOKI 3664 optical power meter, an average photonic power output of P_{out} = 45 mW/cm^2 = 0.045 W/cm^2 at λ_{max} was determined.

The energy of one mol of photons (one einstein) E´ of λ_{max} = 463 nm can be calculated by eq. 1.

$$E' = N_A \cdot E_P = N_A \cdot \frac{hc}{\lambda} \qquad (eq. 1)$$

with N_A being Avogadro´s constant (6.022·10^{23}), h being Planck´s constant, and c being the speed of light. For $\lambda = \lambda_{max} = 463 \cdot 10^{-9}$ m, E´ = 258 kJ·mol^{-1}. Considering an equal numerical distribution of photons at longer and shorter wavelengths with respect to the emission maximum of the LED stick, i.e. a symmetric emission profile with respect to the emission maximum (see Figure S7), all subsequent calculations are performed under the assumption of monochromatic irradiation of $\lambda = \lambda_{max}$ = 463 nm.

The number of photons emitted by 1 cm^2 of the utilized LED-stick per 1 s is then given by eq. 2:

$$N_P(1\text{cm}^2/1\text{s}) = \frac{P_{out}}{E'} \qquad (eq. 2)$$

With P_{out} and E´ given above, N_P(1cm^2/1s) calculates to 1.7·10^{-7} mol·s^{-1} = 0.17 µmol·s^{-1} . As the LED-stick is aligned in a distance of 1 cm and parallel to the vertical axis of the 1 cm x 1 cm cuvette which is filled with 3 mL of photocatalytically active solution, an area of 3 cm^2 is irradiated by the LED-stick. Thus, the cuvette is receiving in total ca. 0.52 µmol·s^{-1} (N_P(3cm^2/1s)) with N_P(3cm^2/1s) = 3·N_P(1cm^2/1s) and these photons are all assumed to enter the cuvette.

In order to calculate the number of photons absorbed by the photocatalysts of the solution per second, the transmission T of light has to be calculated and then multiplied with N_P(3cm^2/1s). T can be calculated by eq. 3 with OD(463nm) being the optical density at 463 nm due to absorbance of the photocatalyst. With typical extinction coefficients of ca. 20˙000 L·mol^{-1}·cm^{-1} at the MLCT maximum for Ru polypyridyl complexes, the 5 µM solutions give an OD(463 nm) of ca. 0.1.

$$T = \frac{1}{10^{OD(463\text{nm})}} \qquad (eq. 3)$$

For such solutions, T = 0.8.

Thus, the number of absorbed photons per second by the 3 mL photocatalytic solution $N_{P,abs}$(3cm^2/1s) is calculated by eq. 4:

$$N_{P,abs}(3\text{cm}^2/1\text{s}) = (1 - T) \cdot N_P(3\text{cm}^2/1\text{s}) \qquad (eq. 4)$$

In the specific example $N_{P,abs}$(3cm^2/1s) = 1.1·10^{-7} mol·s^{-1} = 0.11 µmol·s^{-1}. The 5 µM solution with V = 3 mL contains n(Ru) = 0.015 µmol Ru complex. Thus, the average number N_{exc}(1s) of excited states formed by each Ru complex is calculated by eq. 5:

$$N_{exc}(1s) = \frac{N_{P,abs}(3\text{cm}^2/1\text{s})}{n(Ru)} \qquad (eq. 5)$$

In the specific example this gives N_{exc}(1s) = 7.2·s^{-1}. This means that in this specific example a turnover frequency (TOF) of 7.2 s^{-1}, i.e. a turnover number (TON) of 7.2 in 1 s, correlates to a quantum yield (QY)

of 100%. As the quantum yield is generally formulated by the number of products N_{prod} divided by the number of excited states N_{exc} according to eq. 6,

$$QY = \frac{N_{prod}}{N_{exc}} \qquad (eq. 6)$$

this can also be reformulated to eq. 7 when the TOF is given in the unit s^{-1}:

$$QY = \frac{TOF}{N_{exc}(1\text{s})} \qquad (eq. 7)$$

The QYs reported in the SI and the manuscript were reported using eq. 7 and eq. 8:

$$TOF = \frac{TON}{t_{irr}} \qquad (eq. 8)$$

with t_{irr} being the irradiation time (typically given in seconds in this study).

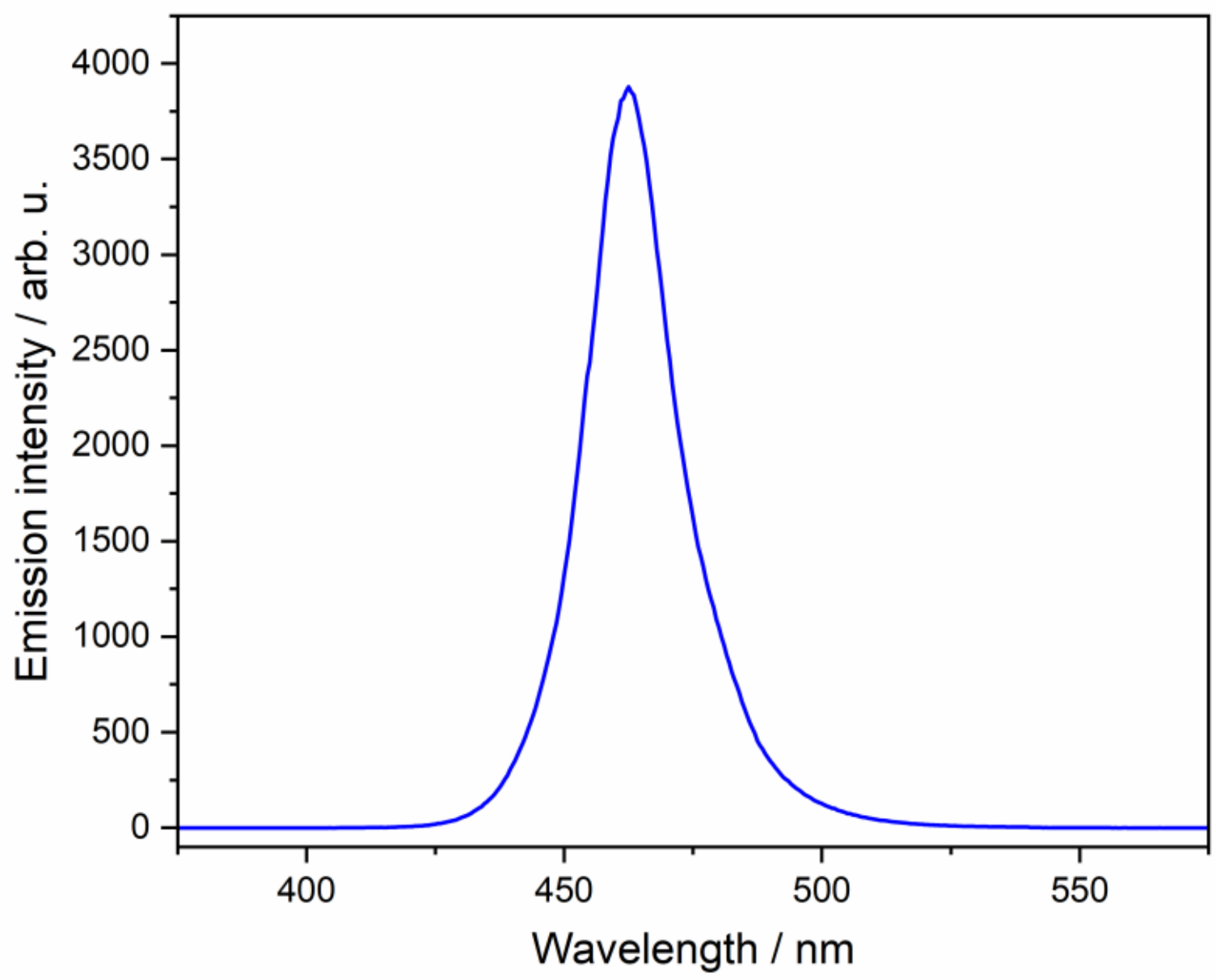

Figure S7. Emission profile of the utilized LED-stick with λ_{max} = 463 nm and FWHM = 20 nm.

3. Supporting Figures (photocatalysis, mechanistic insights, structural characterization)

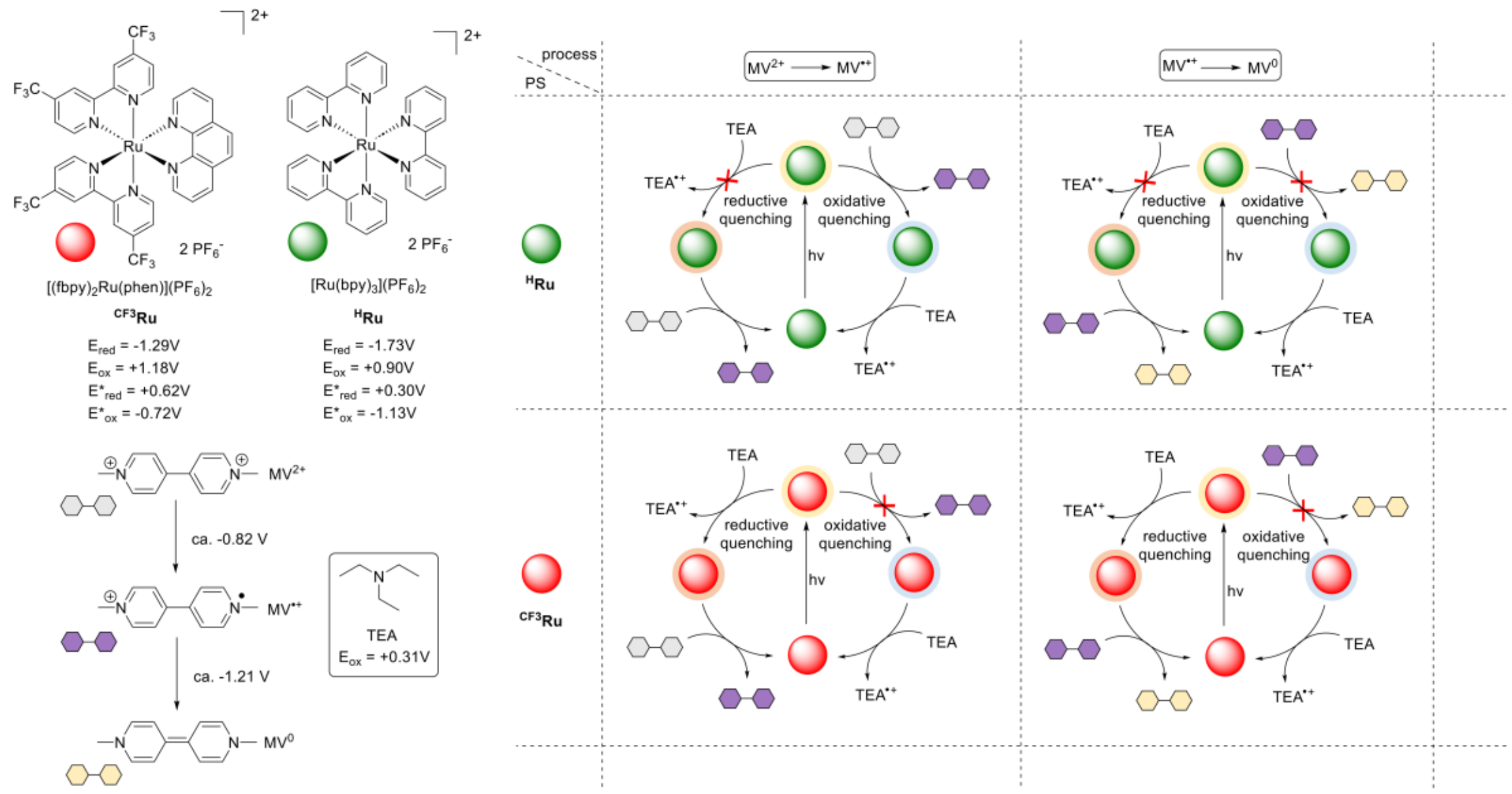

Scheme S1. Molecular structures of ^{CF3}**Ru** and ^{H}**Ru** (top left) and reduction processes of MV^{2+} (bottom left) along with the associated redox potentials (all given vs. Fc^{+}/Fc and taken from literature)[4–8] as well as mechanistic rationalization of the observed photoreactivity (right) using TEA as SED (right). Red crosses in the reaction schemes indicate thermodynamically unlikely / not occurring processes.

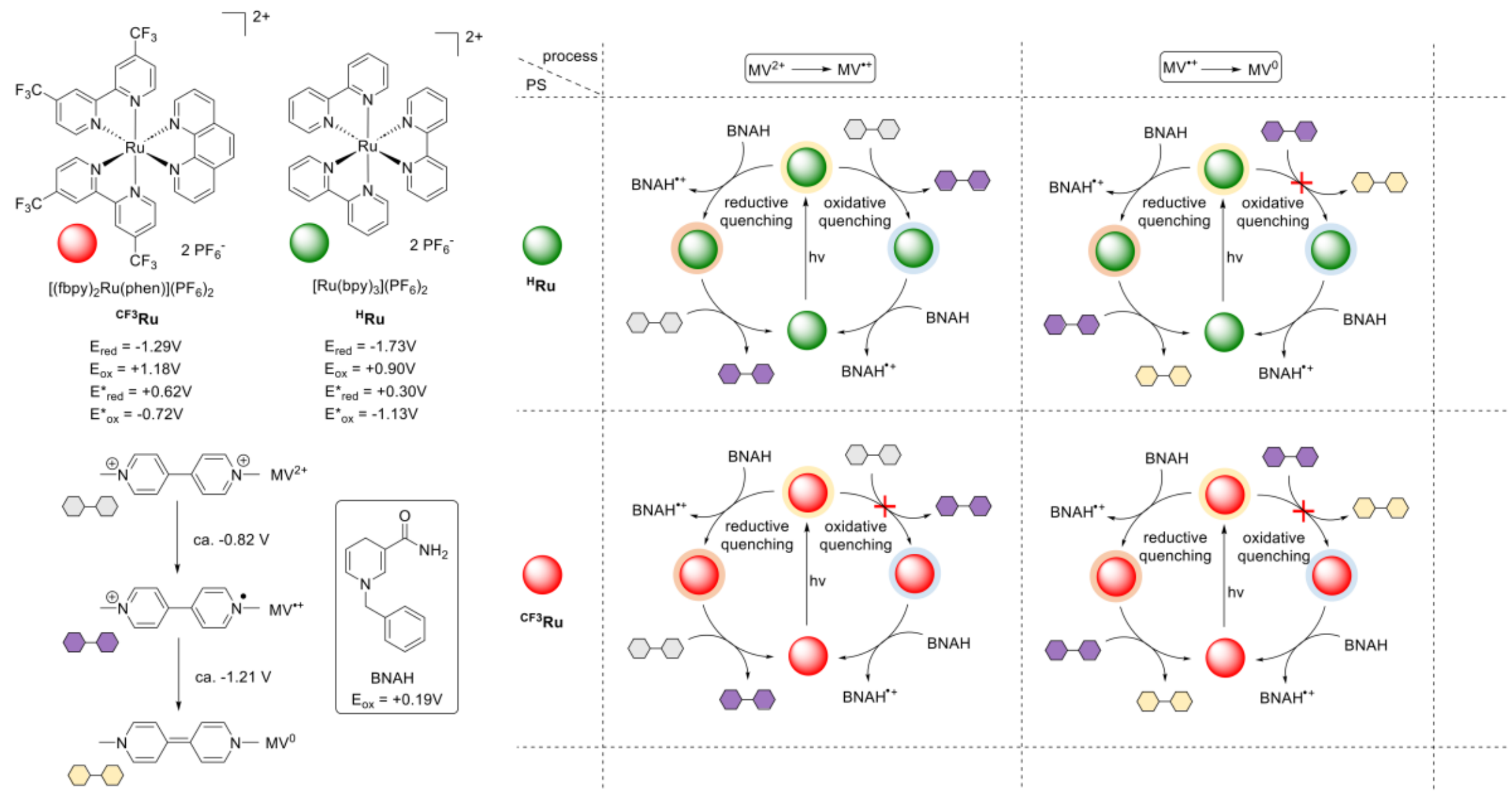

Scheme S2. Molecular structures of ^{CF3}**Ru** and ^{H}**Ru** (top left) and reduction processes of MV^{2+} (bottom left) along with the associated redox potentials (all given vs. Fc^{+}/Fc and taken from literature)[4–8] as well as mechanistic rationalization of the observed photoreactivity (right) using BNAH as SED (right). Red crosses in the reaction schemes indicate thermodynamically unlikely / not occurring processes.

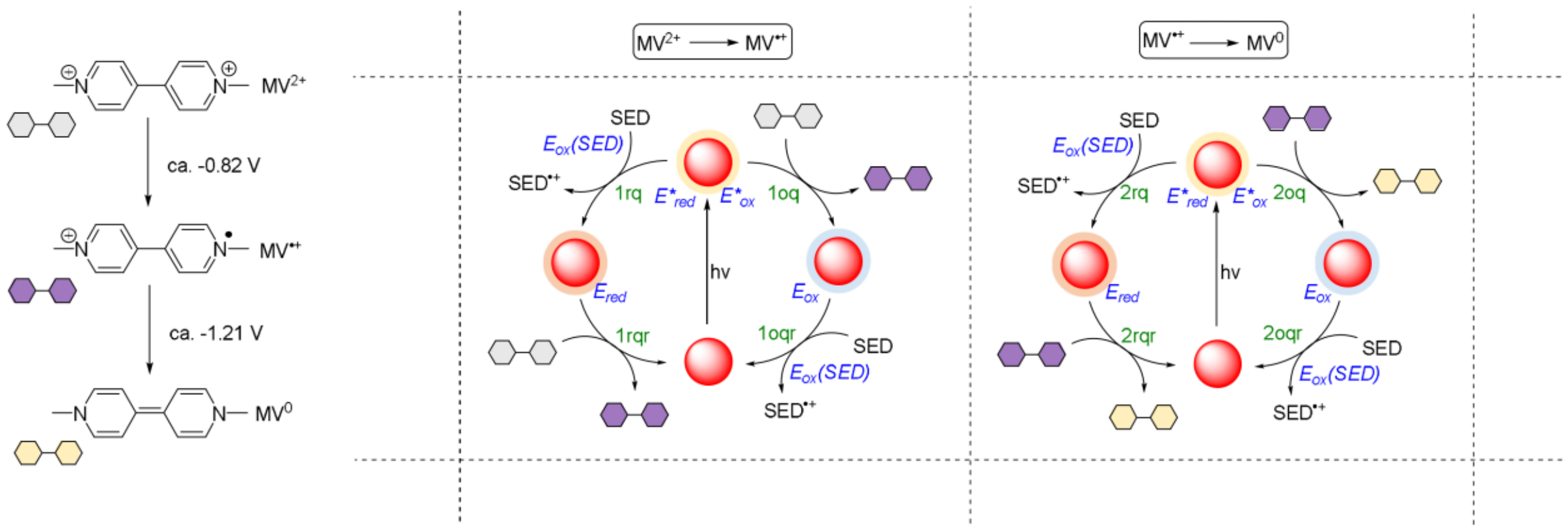

Scheme S3. Left: Reaction sequence for the stepwise twofold reduction of MV^{2+} along with its symbolic representation and the reported potentials vs. Fc^{+}/Fc.[6] Right: Generalized mechanism with potentials given in blue and abbreviations for the individual reaction steps in green to allow understanding of Table S1 (SED = sacrificial electron donor).

Table S1. Overview of possible redox potential combinations for 18 hypothetical PSs and their possibilities to be involved in the 8 elementary steps defined in Scheme S3 as well as the hypothetical outcome of the reaction, i.e., if MV^{2+} can be reduced and if so, whether only $MV^{\bullet+}$ or MV^{0} can be accessed and by which pathway this will occur. Only if two "y" entries can be found next to each other in either the rq-path or the oq-path columns, this indicates closing of the associated photocatalytic cycle and thus access to the respective reduced MV state by this pathway. The color code of the columns should help to identify the PS potentials associated with the individual elementary steps. Categorization for the herein utilized Ru complexes is as follows: ^{H}**Ru**/TEA (#11), ^{H}**Ru**/BNAH (#17), ^{CF3}**Ru**/TEA (#16), ^{CF3}**Ru**/BNAH (#17). The fact that the theoretically derived outcomes match those observed in the experiments grant these rational considerations predictive power. Abbreviations are as follows: n = no, y = yes, n.a. = not applicable, SED = sacrificial electron donor, PS = photosensitizer, oq-path = oxidative quenching pathway, rq-path = reductive quenching pathway. The colors highlight which elementary steps defined in Scheme S3 are related to which redox potential.

	reactions involving PS and SED		reactions involving PS and MV		$MV^{2+} \rightarrow MV^{\bullet+}$				$MV^{\bullet+} \rightarrow MV^{2+}$					
					rq-path		oq-path		rq-path		oq-path			
**#**	**E_{ox}(PS)**	**E^*_{red}(PS)**	**E_{red}(PS)**	**E^*_{ox}(PS)**	**1rq**	**1rqr**	**1oq**	**1oqr**	**2rq**	**2rqr**	**2oq**	**2oqr**	**$MV^{\bullet+}$ accessible?**	**MV^{0} accessible?**
1	< E_{ox}(SED)	< E_{ox}(SED)	> -0.8V	> -0.8V	n	n.a.	n	n.a.	n	n.a.	n	n.a.	n	n
2	< E_{ox}(SED)	< E_{ox}(SED)	-1.2V < -0.8V	> -0.8V	n	n.a.	n	n.a.	n	n.a.	n	n.a.	n	n
3	< E_{ox}(SED)	< E_{ox}(SED)	-1.2V < -0.8V	-1.2V < -0.8V	n	n.a.	y	n	n	n.a.	n	n.a.	n	n
4	< E_{ox}(SED)	< E_{ox}(SED)	< -1.2V	> -0.8V	n	n.a.	n	n.a.	n	n.a.	n	n.a.	n	n
5	< E_{ox}(SED)	< E_{ox}(SED)	< -1.2V	-1.2V < -0.8V	n	n.a.	y	n	n	n.a.	n	n.a.	n	n
6	< E_{ox}(SED)	< E_{ox}(SED)	< -1.2V	< -1.2V	n	n.a.	y	n	n	n.a.	y	n	n	n
7	> E_{ox}(SED)	< E_{ox}(SED)	> -0.8V	> -0.8V	n	n.a.	n	n.a.	n	n.a.	n	n.a.	n	n
8	> E_{ox}(SED)	< E_{ox}(SED)	-1.2V < -0.8V	> -0.8V	n	n.a.	n	n.a.	n	n.a.	n	n.a.	n	n
9	> E_{ox}(SED)	< E_{ox}(SED)	-1.2V < -0.8V	-1.2V < -0.8V	n	n.a.	y	y	n	n.a.	n	n.a.	y (oq-path)	n
10	> E_{ox}(SED)	< E_{ox}(SED)	< -1.2V	> -0.8V	n	n.a.	n	n.a.	n	n.a.	n	n.a.	n	n
11	> E_{ox}(SED)	< E_{ox}(SED)	< -1.2V	-1.2V < -0.8V	n	n.a.	y	y	n	n.a.	n	n.a.	y (oq-path)	n
12	> E_{ox}(SED)	< E_{ox}(SED)	< -1.2V	< -1.2V	n	n.a.	y	y	n	n.a.	y	y	y (oq-path)	y (oq-path)
13	> E_{ox}(SED)	> E_{ox}(SED)	> -0.8V	> -0.8V	y	n	n	n.a.	y	n	n	n.a.	n	n
14	> E_{ox}(SED)	> E_{ox}(SED)	-1.2V < -0.8V	> -0.8V	y	y	n	n.a.	y	n	n	n.a.	y (rq-path)	n
15	> E_{ox}(SED)	> E_{ox}(SED)	-1.2V < -0.8V	-1.2V < -0.8V	y	y	y	y	y	n	n	n.a.	y (oq-path and rq-path)	n
16	> E_{ox}(SED)	> E_{ox}(SED)	< -1.2V	> -0.8V	y	y	n	n.a.	y	y	n	n.a.	y (rq-path)	y (rq-path)
17	> E_{ox}(SED)	> E_{ox}(SED)	< -1.2V	-1.2V < -0.8V	y	y	y	y	y	y	n	n.a.	y (oq-path and rq-path)	y (rq-path)
18	> E_{ox}(SED)	> E_{ox}(SED)	< -1.2V	< -1.2V	y	y	y	y	y	y	y	y	y (oq-path and rq-path)	y (oq-path and rq-path)

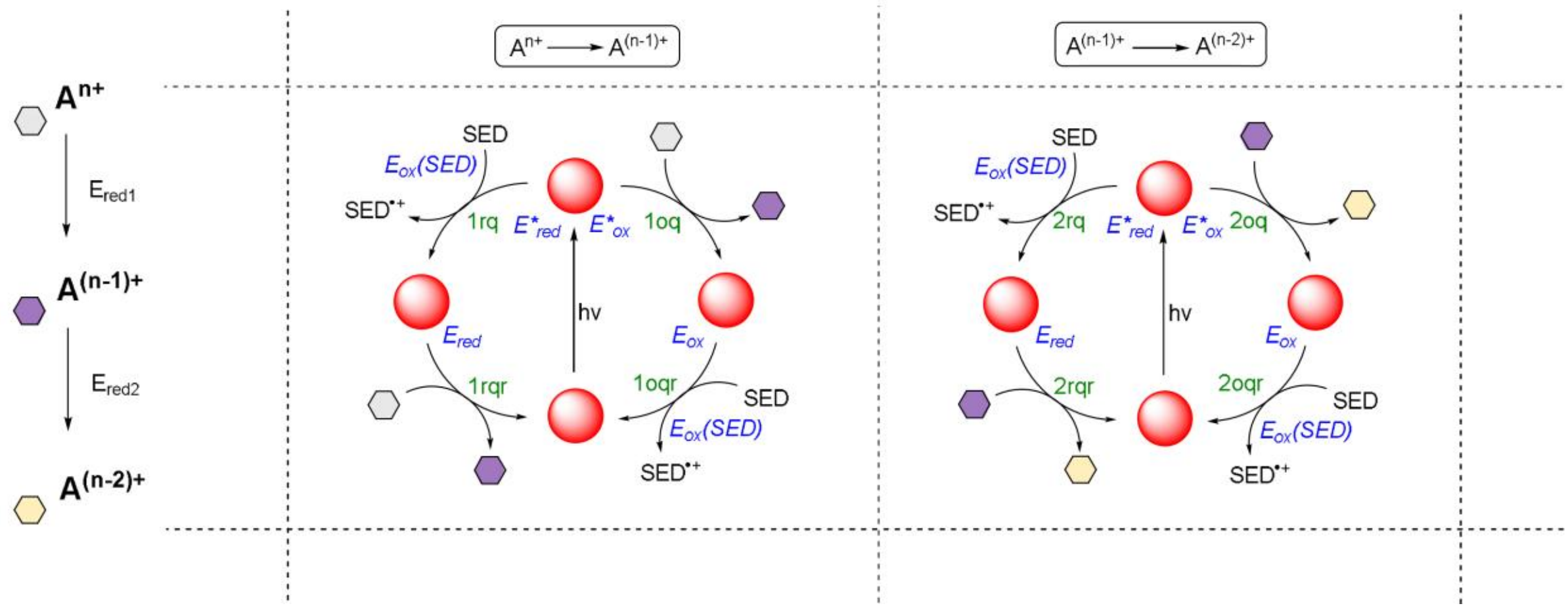

Scheme S4. Left: Reaction sequence for the stepwise twofold reduction of A^{n+} along with its symbolic representation. Right: Generalized mechanism with potentials given in blue and abbreviations for the individual reaction steps in green to allow understanding of Table S2 (SED = sacrificial electron donor).

Table S2. Overview of possible redox potential combinations for 18 hypothetical PSs and their possibilities to be involved in the 8 elementary steps defined in Scheme S4 as well as the hypothetical outcome of the reaction, i.e., if A^{n+} can be reduced and if so, whether only $A^{(n-1)+}$ or $A^{(n-2)+}$ can be accessed and by which pathway this will occur. Only if two "y" entries can be found next to each other in either the rq-path or the oq-path columns, this indicates closing of the associated photocatalytic cycle and thus access to the respective reduced state by this pathway. The color code of the columns should help to identify the PS potentials associated with the individual elementary steps. Abbreviations are as follows: n = no, y = yes, n.a. = not applicable, SED = sacrificial electron donor, PS = photosensitizer, oq-path = oxidative quenching pathway, rq-path = reductive quenching pathway. The colors highlight which elementary steps defined in Scheme S4 are related to which redox potential.

	reactions involving PS and SED		reactions involving PS and A		$A^{n+} + e^- \rightarrow A^{(n-1)+}$				$A^{(n-1)+} + e^- \rightarrow A^{(n-2)+}$					
					rq-path		oq-path		rq-path		oq-path			
#	$E_{ox}(PS)$	$E^*_{red}(PS)$	$E_{red}(PS)$	$E^*_{ox}(PS)$	1rq	1rqr	1oq	1oqr	2rq	2rqr	2oq	2oqr	onefold reduced acceptor $A^{(n-1)+}$ accessible?	twofold reduced acceptor $A^{(n-2)+}$ accessible?
1	$< E_{ox}(SED)$	$< E_{ox}(SED)$	$> E_{red1}(A)$	$> E_{red1}(A)$	n	n.a.	n	n.a.	n	n.a.	n	n.a.	n	n
2	$< E_{ox}(SED)$	$< E_{ox}(SED)$	$E_{red2}(A) < E_{red1}(A)$	$> E_{red1}(A)$	n	n.a.	n	n.a.	n	n.a.	n	n.a.	n	n
3	$< E_{ox}(SED)$	$< E_{ox}(SED)$	$E_{red2}(A) < E_{red1}(A)$	$E_{red2}(A) < E_{red1}(A)$	n	n.a.	y	n	n	n.a.	n	n.a.	n	n
4	$< E_{ox}(SED)$	$< E_{ox}(SED)$	$< E_{red2}(A)$	$> E_{red1}(A)$	n	n.a.	n	n.a.	n	n.a.	n	n.a.	n	n
5	$< E_{ox}(SED)$	$< E_{ox}(SED)$	$< E_{red2}(A)$	$E_{red2}(A) < E_{red1}(A)$	n	n.a.	y	n	n	n.a.	n	n.a.	n	n
6	$< E_{ox}(SED)$	$< E_{ox}(SED)$	$< E_{red2}(A)$	$< E_{red2}(A)$	n	n.a.	y	n	n	n.a.	y	n	n	n
7	$> E_{ox}(SED)$	$< E_{ox}(SED)$	$> E_{red1}(A)$	$> E_{red1}(A)$	n	n.a.	n	n.a.	n	n.a.	n	n.a.	n	n
8	$> E_{ox}(SED)$	$< E_{ox}(SED)$	$E_{red2}(A) < E_{red1}(A)$	$> E_{red1}(A)$	n	n.a.	n	n.a.	n	n.a.	n	n.a.	n	n
9	$> E_{ox}(SED)$	$< E_{ox}(SED)$	$E_{red2}(A) < E_{red1}(A)$	$E_{red2}(A) < E_{red1}(A)$	n	n.a.	y	y	n	n.a.	n	n.a.	y (oq-path)	n
10	$> E_{ox}(SED)$	$< E_{ox}(SED)$	$< E_{red2}(A)$	$> E_{red1}(A)$	n	n.a.	n	n.a.	n	n.a.	n	n.a.	n	n
11	$> E_{ox}(SED)$	$< E_{ox}(SED)$	$< E_{red2}(A)$	$E_{red2}(A) < E_{red1}(A)$	n	n.a.	y	y	n	n.a.	n	n.a.	y (oq-path)	n
12	$> E_{ox}(SED)$	$< E_{ox}(SED)$	$< E_{red2}(A)$	$< E_{red2}(A)$	n	n.a.	y	y	n	n.a.	y	y	y (oq-path)	y (oq-path)
13	$> E_{ox}(SED)$	$> E_{ox}(SED)$	$> E_{red1}(A)$	$> E_{red1}(A)$	y	n	n	n.a.	y	n	n	n.a.	n	n
14	$> E_{ox}(SED)$	$> E_{ox}(SED)$	$E_{red2}(A) < E_{red1}(A)$	$> E_{red1}(A)$	y	y	n	n.a.	y	n	n	n.a.	y (rq-path)	n
15	$> E_{ox}(SED)$	$> E_{ox}(SED)$	$E_{red2}(A) < E_{red1}(A)$	$E_{red2}(A) < E_{red1}(A)$	y	y	y	y	y	n	n	n.a.	y (oq-path and rq-path)	n
16	$> E_{ox}(SED)$	$> E_{ox}(SED)$	$< E_{red2}(A)$	$> E_{red1}(A)$	y	y	n	n.a.	y	y	n	n.a.	y (rq-path)	y (rq-path)
17	$> E_{ox}(SED)$	$> E_{ox}(SED)$	$< E_{red2}(A)$	$E_{red2}(A) < E_{red1}(A)$	y	y	y	y	y	y	n	n.a.	y (oq-path and rq-path)	y (rq-path)
18	$> E_{ox}(SED)$	$> E_{ox}(SED)$	$< E_{red2}(A)$	$< E_{red2}(A)$	y	y	y	y	y	y	y	y	y (oq-path and rq-path)	y (oq-path and rq-path)

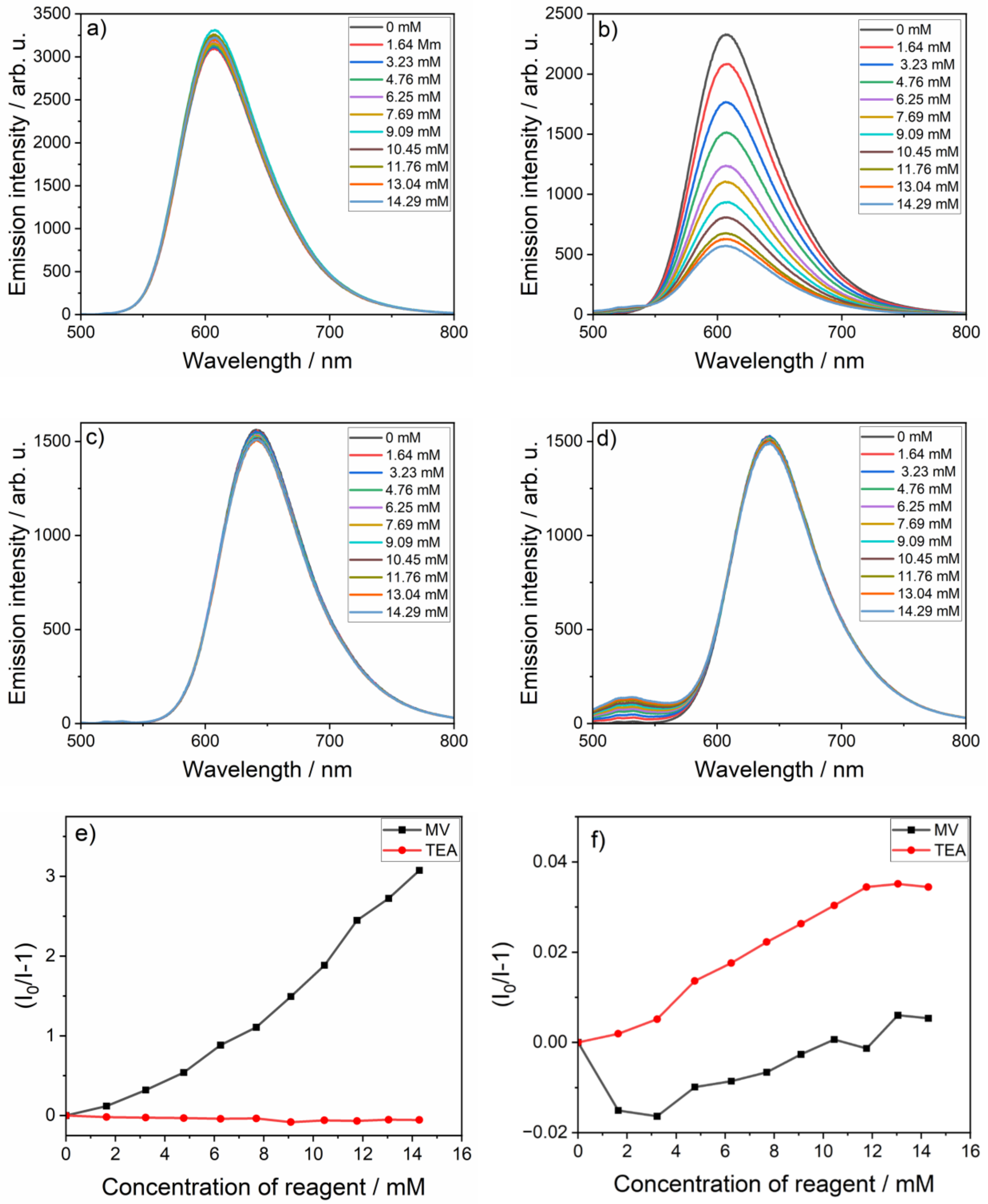

Figure S8 (a-d) Luminescence quenching data for 5 µM ^{H}**Ru** (a, b) and 5 µM ^{CF3}**Ru** (c, d) in MeCN/H_2O = 1:1 (v:v) upon addition of increasing concentrations of TEA (a, c) and MV^{2+} (b, d), respectively, under aerobic conditions. (e–f) Corresponding Stern–Volmer plots illustrating the quenching behavior for each quencher; panel e) shows the data for ^{H}**Ru** while panel f) does for ^{CF3}**Ru**. Interestingly, even the reductive quenching for ^{CF3}**Ru** by TEA is rather limited (panels c and f), a significant boost of photocatalytic activity was observed.

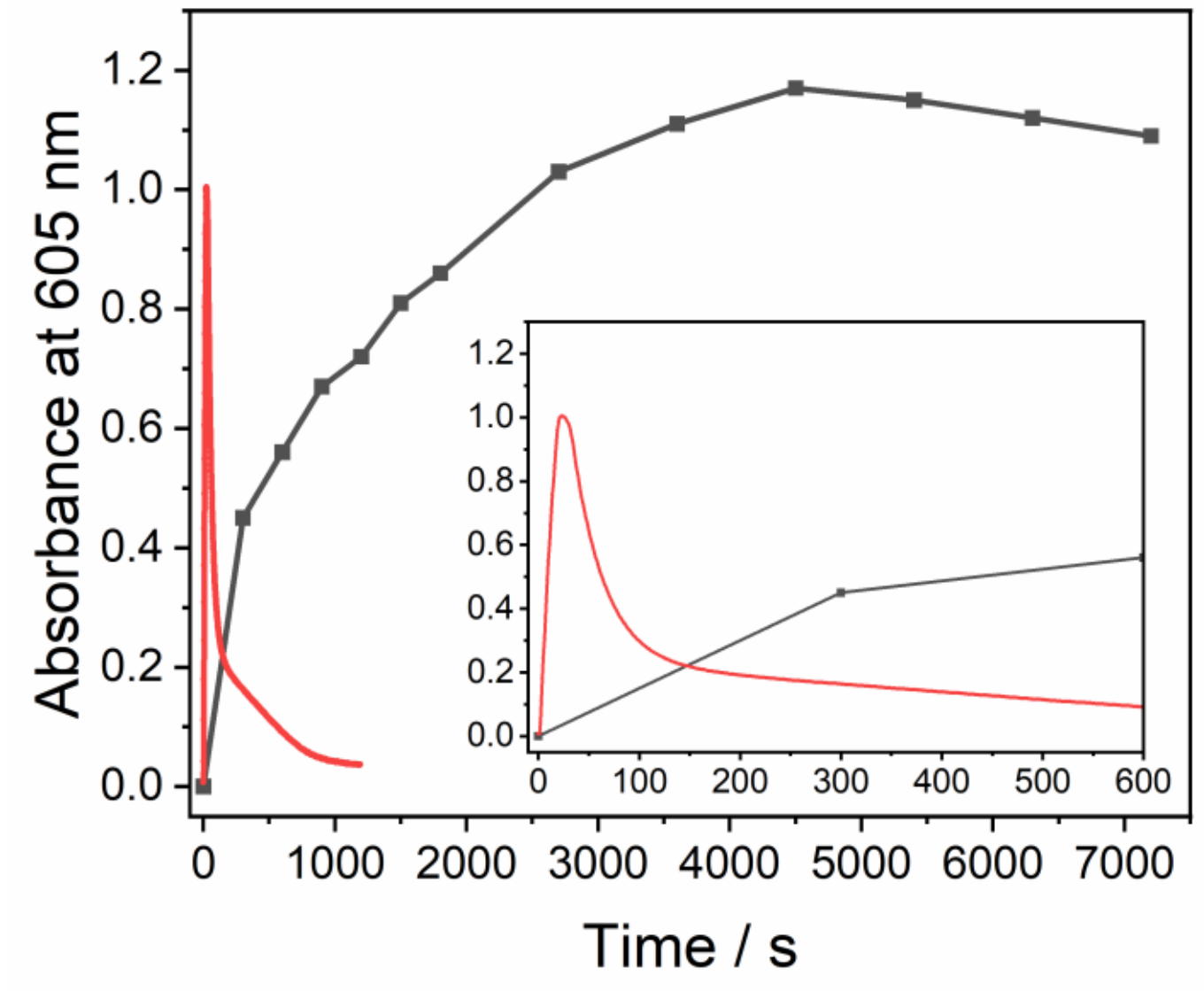

Figure S9. Absorbance changes at 605 nm during the MV^{2+} (100 µM) photoreduction using **^{H}Ru** (5 µM, grey data) and **^{CF3}Ru** (5 µM, red data) in MeCN:H_2O = 1:1 (v:v) in the presence of 0.12 M TEA and 0.1 M NaH_2PO_4 buffer. The inset highlights the obtained data within the first 600 s. The 23 s needed to reach full monoreduction of MV^{2+} with 5 µM **^{CF3}Ru** corresponds to a QY of ca. 12%, and the 4500 s for 5 µM **^{H}Ru** to a QY of ca. 0.06%. Similarly low QYs for ^{H}Ru at low MV^{2+} concentrations has previously been reported as well.[9]

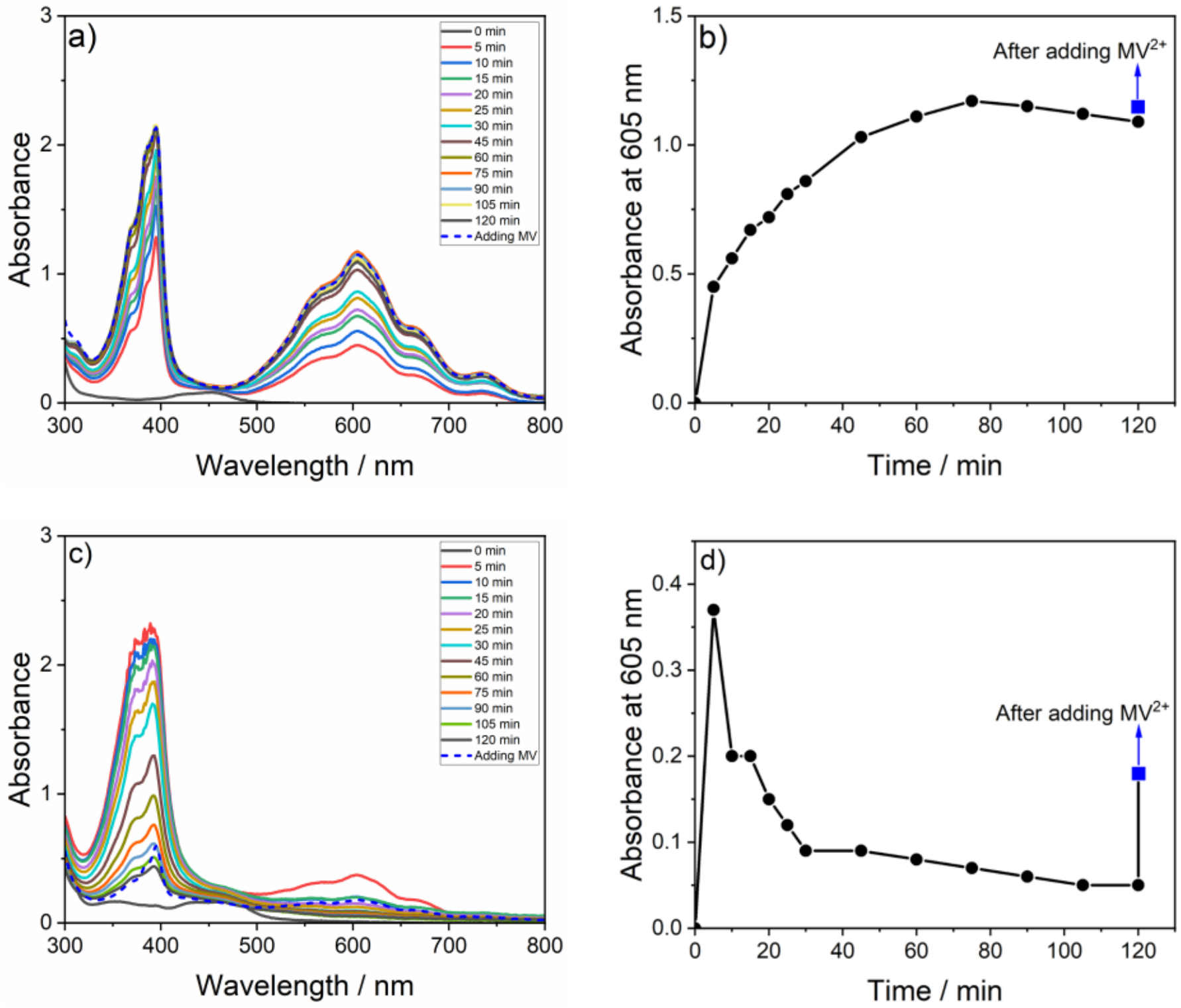

Figure S10. Steady-state UV/vis-spectroscopic changes of **^{H}Ru** (panel a) and **^{CF3}Ru** (panel c) during MV^{2+} (100 µM) photoreduction in MeCN:H_2O = 1:1 (v:v) containing 0.12 M TEA and 0.10 M NaH_2PO_4. The samples were irradiated for 120 min and afterwards 31 µL of a 9.8 mM MV^{2+} stock solution in H_2O, equal to 100 µM fresh MV^{2+} in the final solution, was added. The spectra obtained after MV^{2+} addition is given as dashed lines (panels a and c). The absorbance at 605 nm for both samples is shown in panels b and d. The changes upon final MV^{2+} addition is indicated by an arrow and highlighted by a blue square. Similar comproportionation experiments to verify the build-up of MV^{0} have been published.[10]

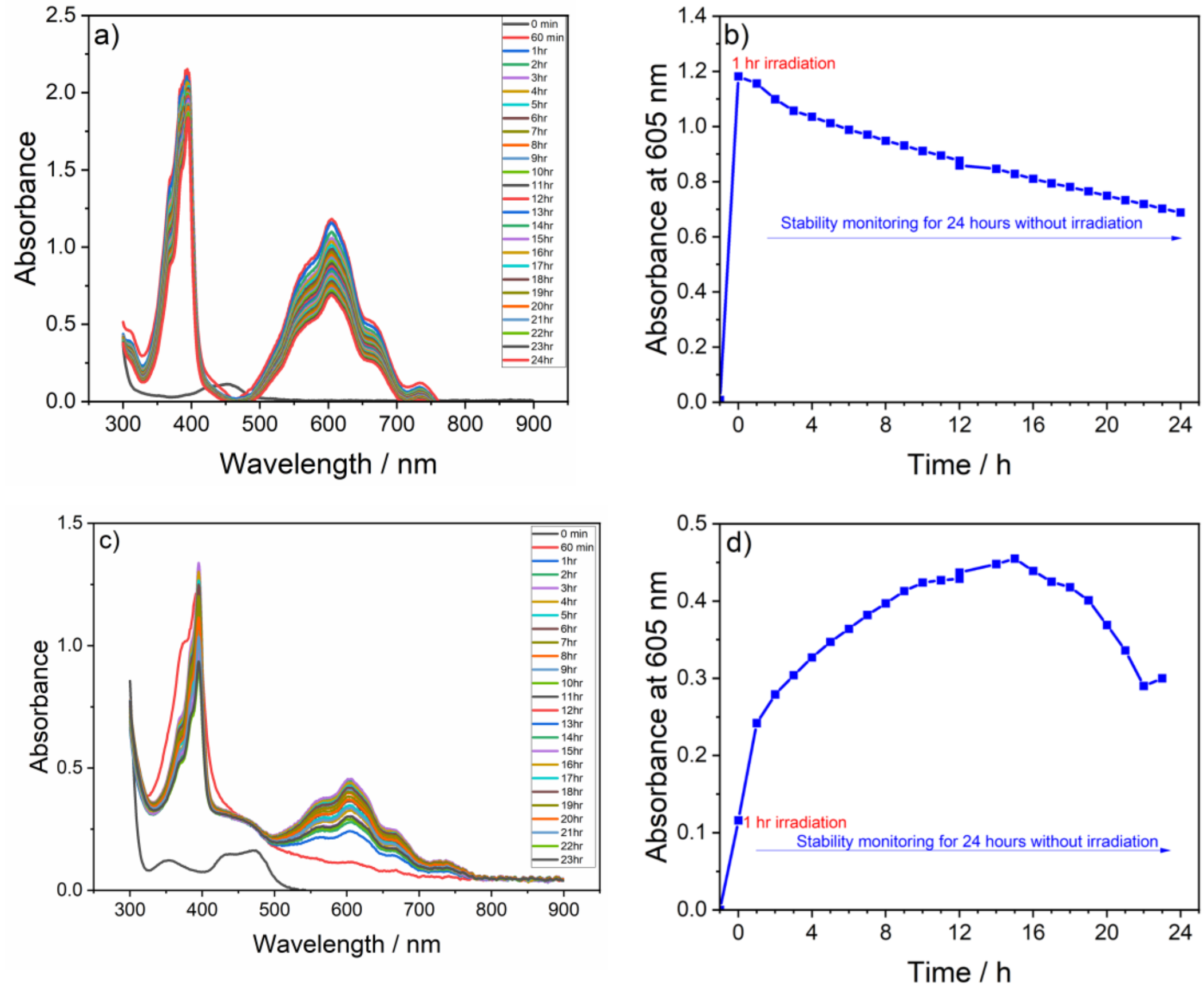

Figure S11. Steady-state UV/vis-spectroscopic changes of ^{H}**Ru** (panel a) and ^{CF3}**Ru** (panel c) during MV^{2+} (100 µM) following photoreduction in MeCN:H_2O = 1:1 (v:v) containing 0.12 M TEA and 0.10 M NaH_2PO_4 for 60 min. The corresponding absorbance changes at 605 nm for ^{H}**Ru** (panel b) and ^{CF3}**Ru** (panel d) while the samples are stored under the exclusion of light indicate reoxidation of photoreduced products even inside the Ar-filled GB. This might be attributed to reaction with degradation products of photooxidized TEA.

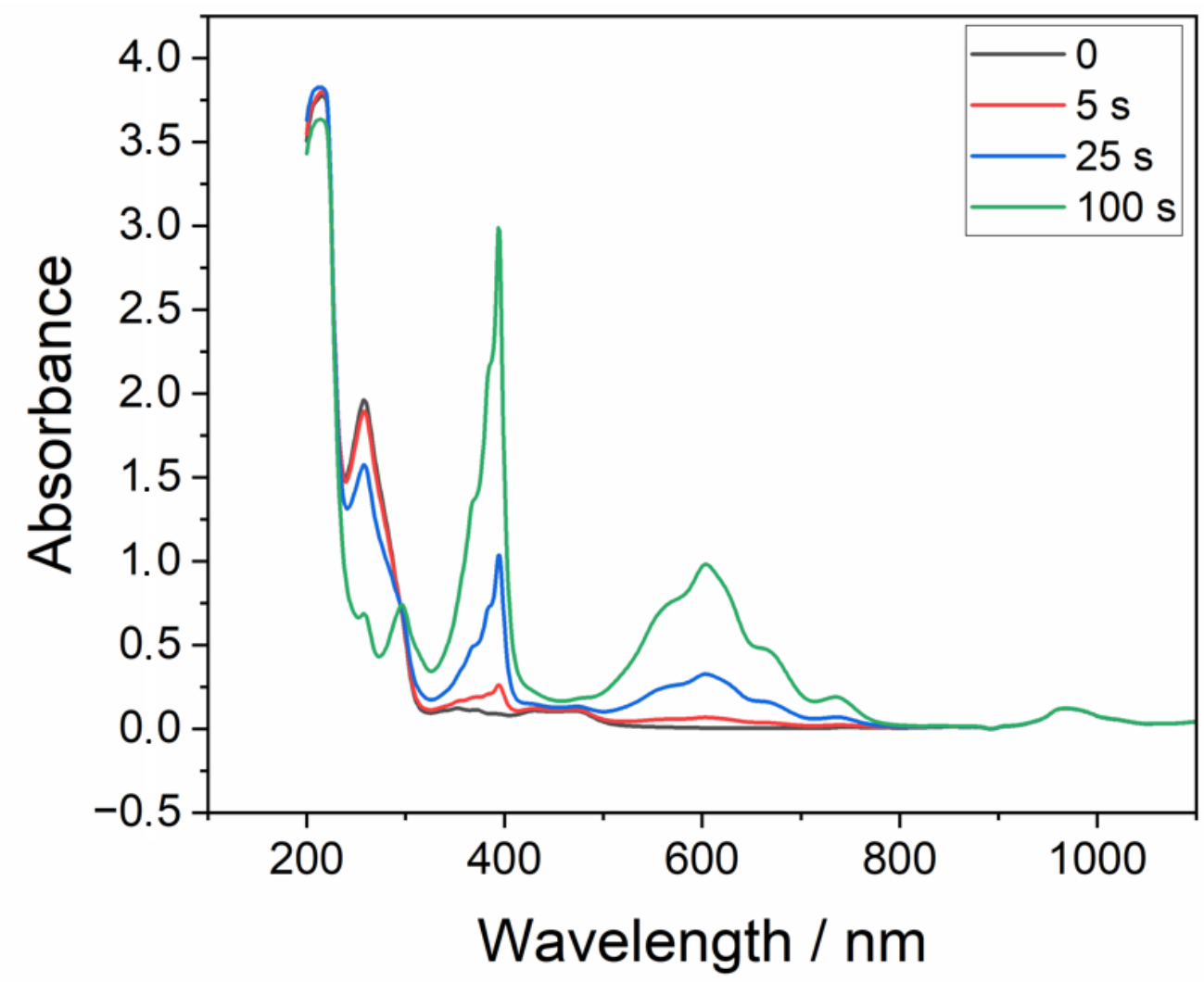

Figure S12. Steady-state UV-Vis absorption spectra during the photocatalytic reduction of 100 µM MV^{2+} using ^{CF3}**Ru** in MeCN:H_2O = 1:1 (v:v) containing 0.12 M TEA and 0.1 M NaH_2PO_4. Spectra were recorded from 200 nm - 1100 nm utilizing a JASCO V-670 spectrometer. Irradiation with was performed from the bottom of the cuvette using one LED stick ($\lambda_{emi,max}$ = 463 nm, P_{out} = 45 mW/cm^2).

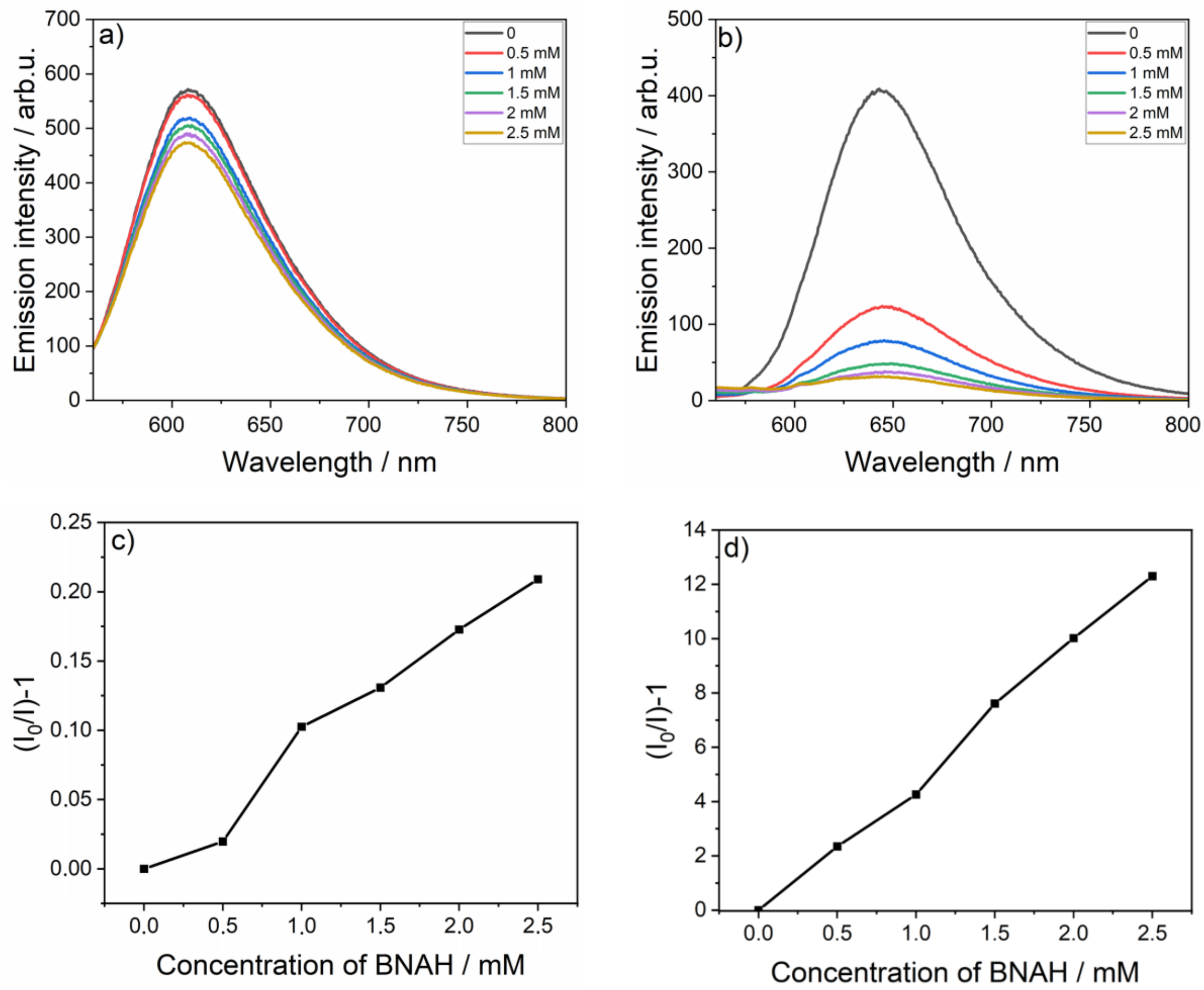

Figure S13. (a-b) Luminescence quenching data for ^{H}**Ru** (a) and ^{CF3}**Ru** (b) (5 µM each) in MeCN/H_2O = 1:1 (v:v) upon addition of increasing concentrations of BNAH under an argon atmosphere; λ_{exc} = 510 nm. (c–d) Corresponding Stern–Volmer plots illustrating the quenching behavior for ^{H}**Ru** (c; K_{SV} = 87 M^{-1}) and ^{CF3}**Ru** (d; K_{SV} = 5000 M^{-1}) respectively. The lines are added to guide the eye and have no physical meaning.

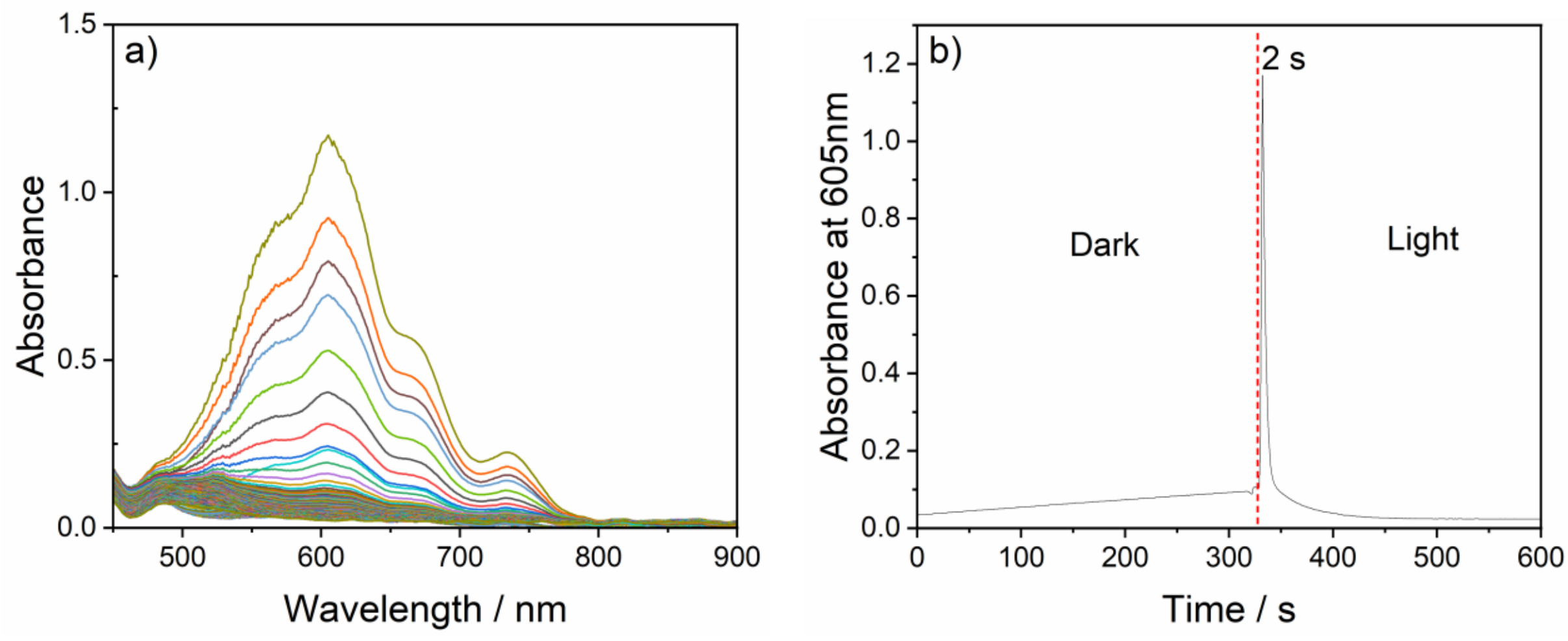

Figure S14. (a) UV-Vis absorption spectra and (b) absorbance intensity at 605 nm vs. time plot for **^{CF3}Ru** (5 µM) in a solution containing 100 µM MV^{2+} and 10 mM BNAH in MeCN/H_2O = 9:1 (v:v). For the first ca. 5 minutes the sample was kept in the dark, followed another ca. 5 minutes of irradiation. The red dashed line in panel b indicates the time at which the LED array was turned on. The 2 s needed to reach full monoreduction of MV^{2+} with 5 µM **^{CF3}Ru** corresponds to a QY of ca. 140%, indicating also light-independent reduction of MV^{2+} by the degradation products of BNAH.

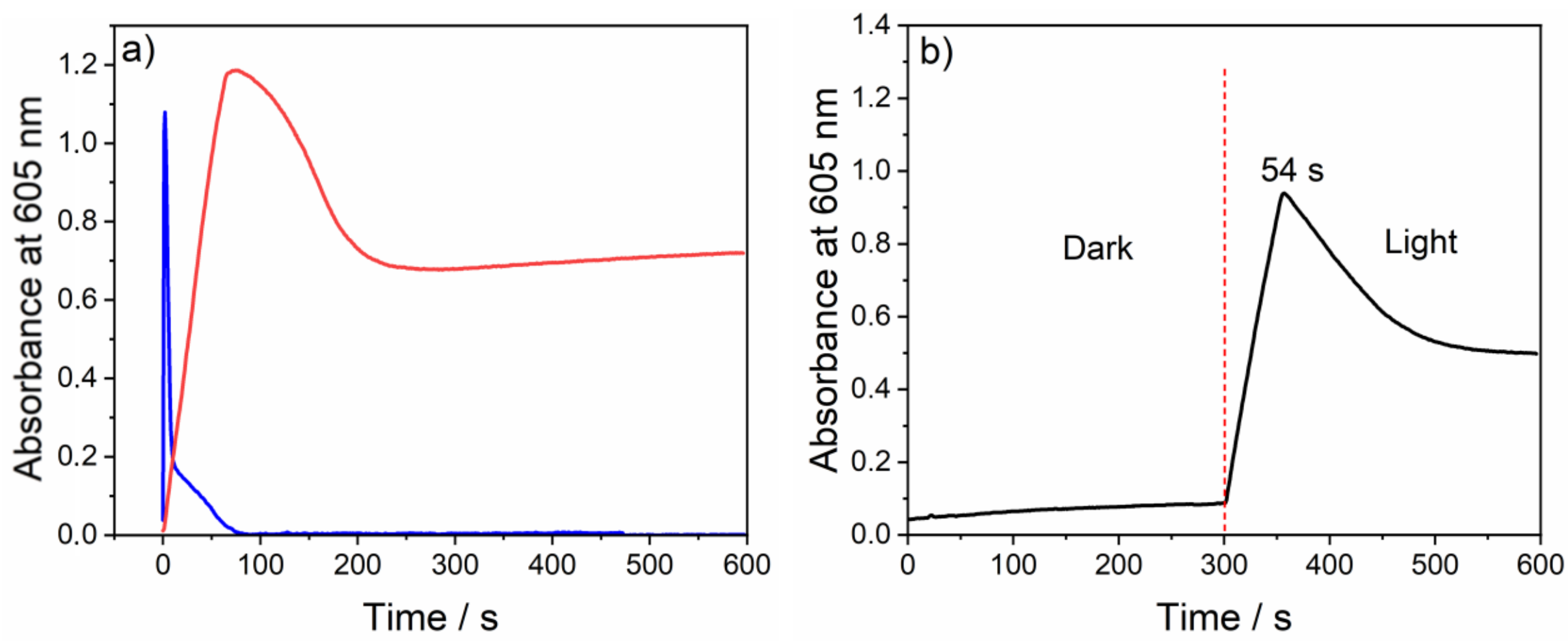

Figure S15. (a) Absorbance changes at 605 nm during MV^{2+} (100 µM) photoreduction for samples containing (blue curve) or not containing (red curve) 5 µM **^{H}Ru** in MeCN:H_2O = 9:1 (v:v); both samples contained 10 mM BNAH. (b) Absorbance changes at 605 nm for a solution containing 100 µM MV^{2+} and 10 mM BNAH in MeCN/H_2O = 9:1 (v:v). For the first 5 minutes the sample was kept in the dark, followed by 5 minutes of irradiation. The dotted line in panel b indicates the time at which the LED array was turned on.

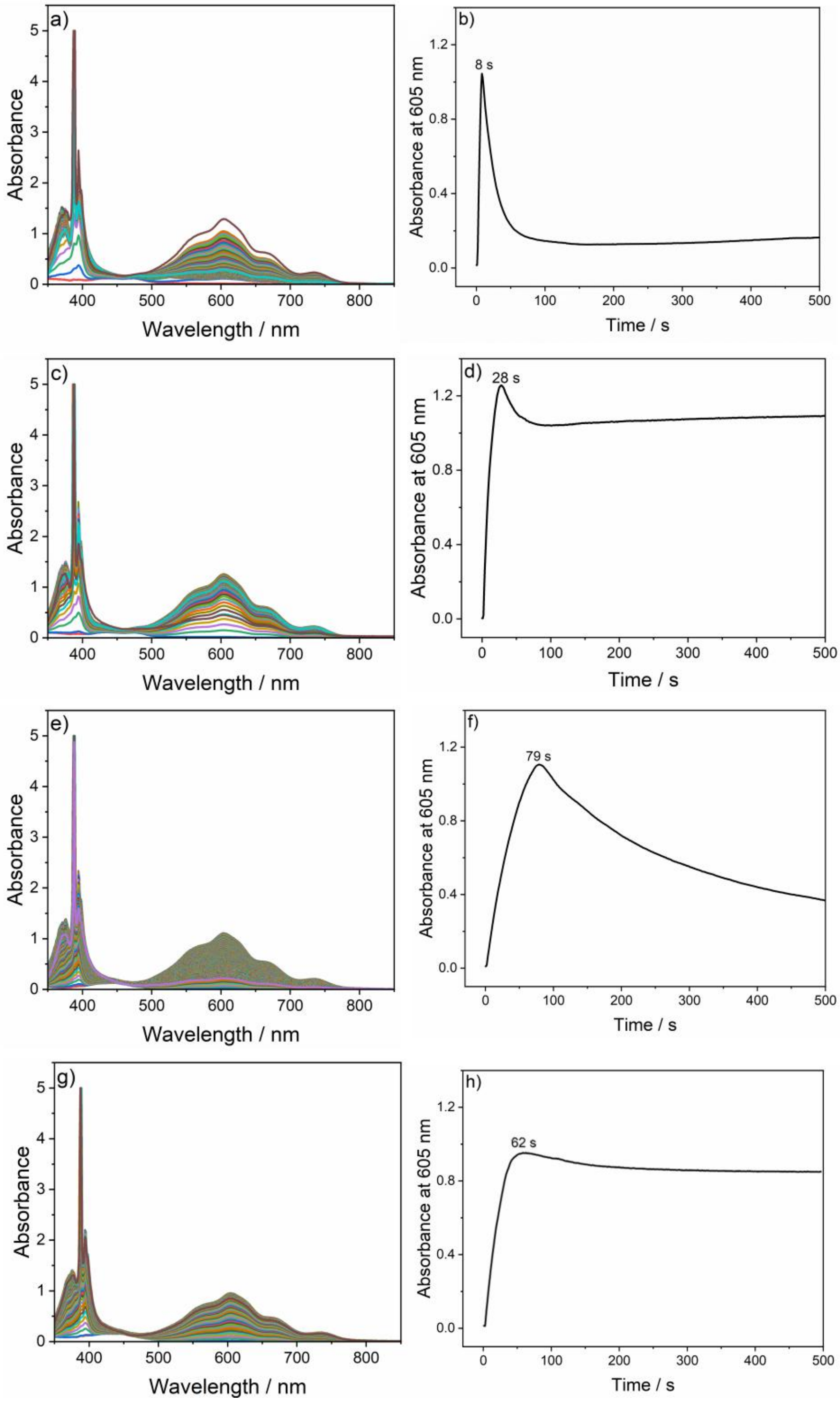

Figure S16. (a,c) Steady-state UV-Vis absorption spectra and (b,d) absorbance intensity at 605 nm over time for **CF3Ru** in a solution containing 100 µM MV^{2+} and different concentrations of BNAH (0.1 mM (panels a,b) and 0.05 mM (panels c, d)) in MeCN/H_2O = 9:1 (v:v). Only the 1:1 combination enabled MV^0 formation (panels a and b) whereas the 0.5:1 ratio stopped at the $MV^{\bullet+}$ state (panels c and d). The overshooting signal in panel d prior to reaching a plateau is ascribed to slow diffusion processes inside the one-sided irradiated and non-stirred cuvette. The 8 s and 28 s to reach full MV^{2+} monoreduction corresponds to QYs of ca. 35 % and 10 %, respectively. (e,g) Steady-state UV-Vis absorption spectra and (f,h) absorbance intensity at 605 nm over time for **HRu** in a solution containing 100 µM MV^{2+} and different concentrations of BNAH (0.1 mM (panels e,f) and 0.05 mM (panels g,h)) in MeCN/H_2O = 9:1 (v:v). The 79 s and 62 s to reach full MV^{2+} monoreduction corresponds to QYs of ca. 3.5 % and 4.5 %, respectively.

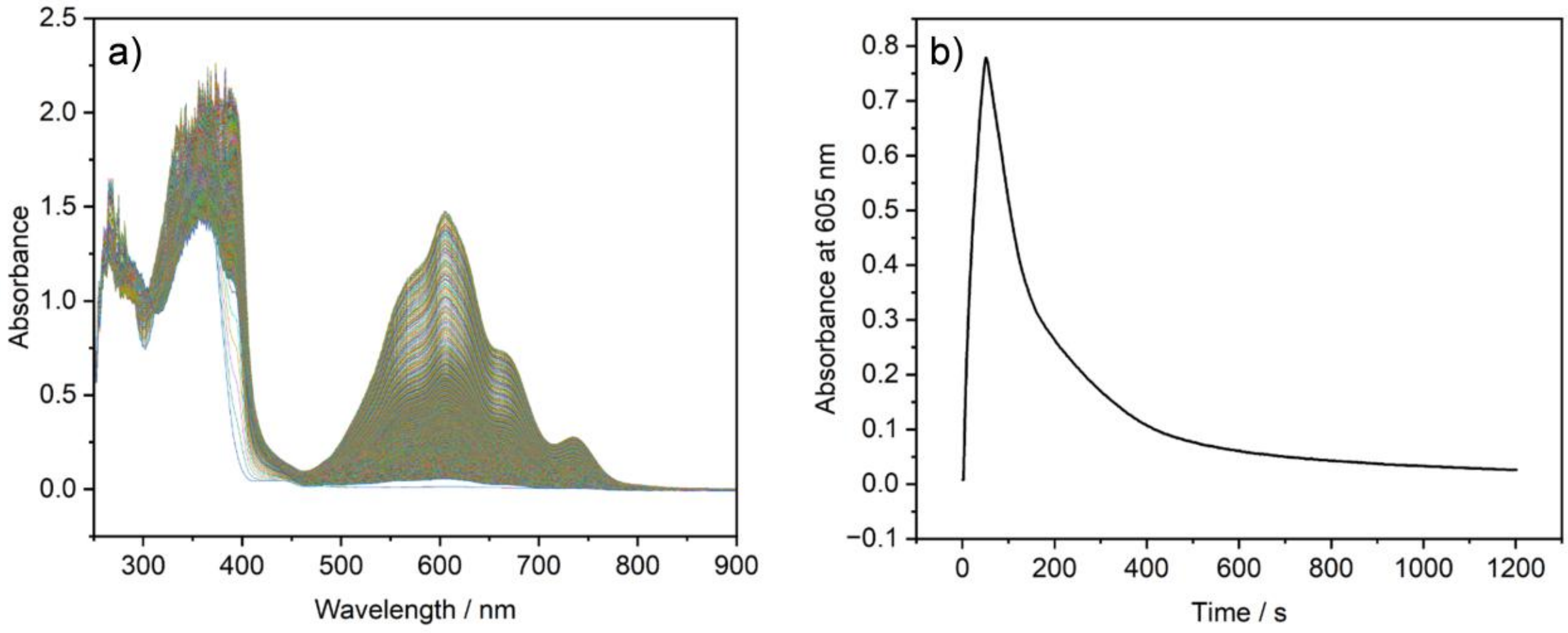

Figure S17. Steady-state UV/vis-spectroscopic changes of **^{H}Ru** (panel a) during MV^{2+} (100 µM) with 0.5 mM BNAH in pure MeCN. The corresponding absorbance changes at 605 nm are depicted in panel b.

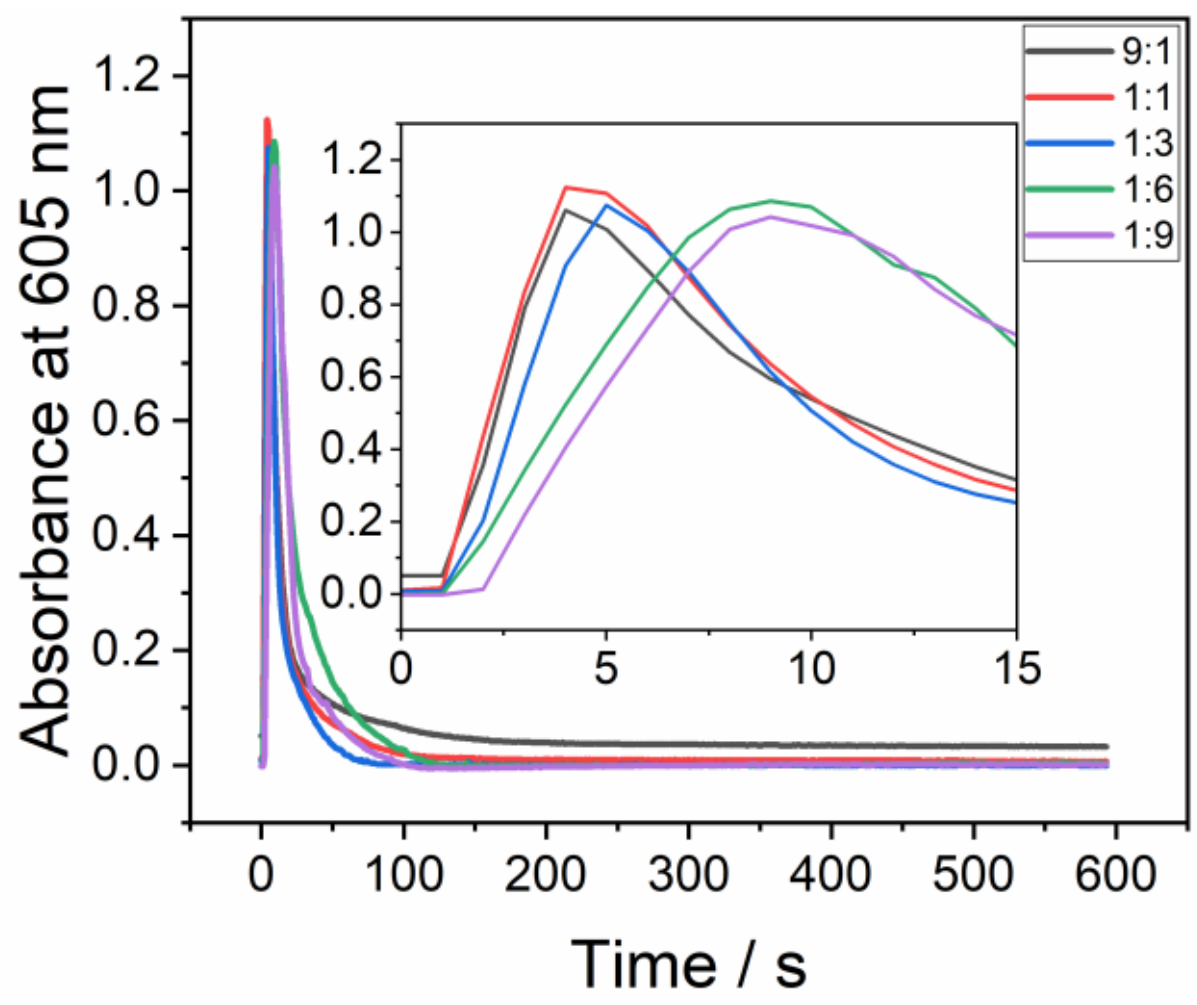

Figure S18. Absorbance changes at 605 nm with increasing irradiation time during MV^{2+} (100 µM) photoreduction using 5 µM **^{CF3}Ru** in different MeCN:H_2O-mixtures (9:1, 1:1, 1:3, 1:6, 1:9, always v:v) containing 0.5 mM BNAH in all cases. The inset highlights the data within the first 15 s of irradiation.

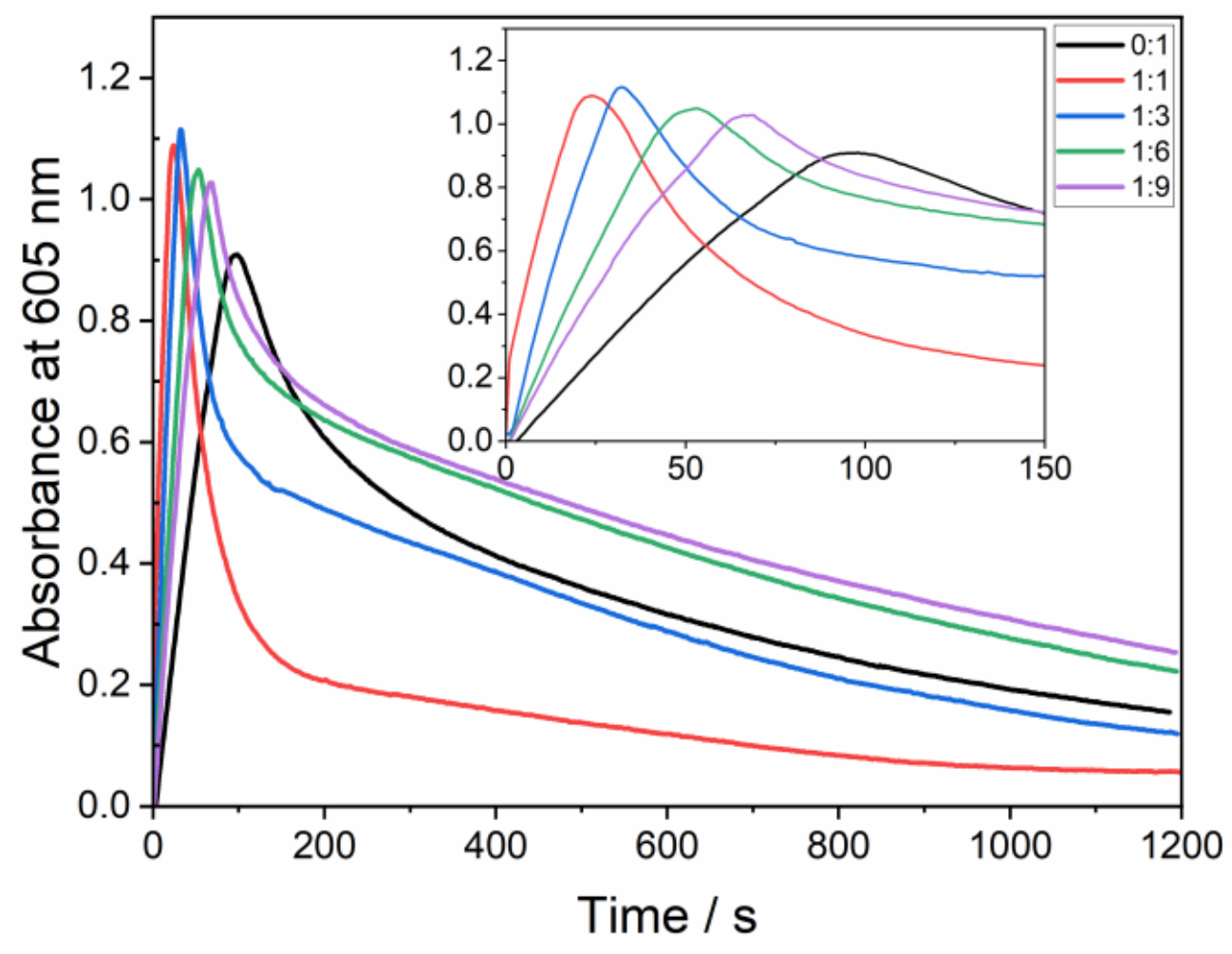

Figure S19. Absorbance changes at 605 nm with increasing irradiation time during MV^{2+} (100 µM) photoreduction using 5 µM **^{CF3}Ru** in different MeCN:H_2O-mixtures (0:1, 1:1, 1:3, 1:6, 1:9; always v:v) containing 0.12 M TEA and 0.10 M NaH_2PO_4 in all cases.

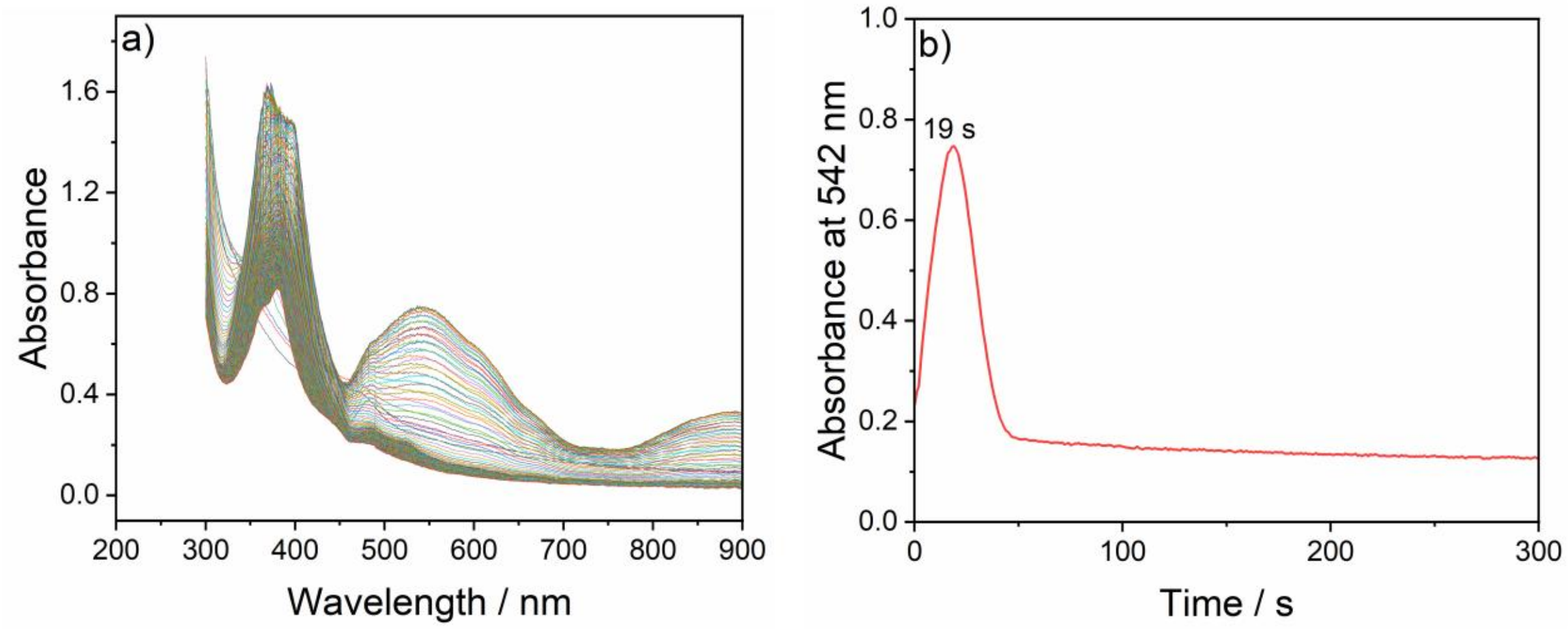

Figure S20. (a) Series of steady-state UV-Vis absorption spectra recorded in 1 s time intervals during the irradiation of a solution containing 5 µM **^{CF3}Ru** and 100 µM polymer-bound benzyl viologen units in MeCN:H_2O = 1:1 (v:v) containing 0.12 M TEA and 0.10 M NaH_2PO_4. (b) Corresponding absorbance changes at 542 nm. The 19 s needed for complete monoreduction of polymer-linked MV^{2+} subunits corresponds to a QY of ca. 15 %.

4. Kinetic model

When varying c(MV^{2+}) from 50 µM to 100 µM and 200 µM using 5 µM of **^{CF3}Ru** in combination with phosphate-buffered TEA, the time required to reach complete one-fold reduction of MV^{2+} was 8 s, 23 s and 63 s. Considering the fact that the product of this reaction, $MV^{•+}$, is co-absorbing at the irradiation wavelength of 463 nm it is not surprising that these numbers strongly deviate from linearity. Assuming stability of all involved compounds absorbing at 463 nm, i.e. **^{CF3}Ru** and $MV^{•+}$, during these short photoreduction experiments, the following kinetic model was set up to verify whether despite this deviation from linearity the photochemical reaction per se was still independent of the concentration of MV^{2+}, i.e. of zeroth order with respect to MV^{2+} (this would clarify the reductive quenching of excited **^{CF3}Ru** as the rate determining step):

By accumulating in solution, the time-dependent share of photons reaching **^{CF3}Ru** instead of getting absorbed by $MV^{•+}$ (ε_P(463 nm) ≈ 1300 $Lmol^{-1}cm^{-1}$,[11]) is given by equation 9

$$f_{cat}(t) = \frac{\epsilon_{cat}c_{cat}}{\varepsilon_{cat}c_{cat} + \varepsilon_P c_P(t)} \qquad (eq.9)$$

where cat in this case indicates **^{CF3}Ru** and P indicates $MV^{•+}$.

For a reaction of zeroth order the reaction rate is formulated as follows (equation 10) with substrate S representing MV^{2+}:

$$\frac{dS}{dt} = -kf_{cat}(t) = -k\frac{\epsilon_{cat}c_{cat}}{\varepsilon_{cat}c_{cat} + \varepsilon_P\big(c(S_0) - c(S)\big)} \qquad (eq.10)$$

Integration of equation 2 allows for the determination of the starting substrate concentration (S_0)-dependent time T(S_0) needed for 100% conversion of MV^{2+} into $MV^{•+}$, as given by equation 11:

$$T(S_0) = \frac{1}{k}\left[S_0 + \frac{\varepsilon_P}{2\varepsilon_{cat}c_{cat}}S_0^2\right] \qquad (eq.11)$$

When fitting equation 3 to the obtained data of 8 s, 23 s and 63 s by using the quadratic equation 12

$$T = \alpha S_0 + \beta S_0^2 \qquad (eq.12)$$

the following values are obtained: α = 0,1314 s/µM → k = α^{-1} = 7,61 µM/s (i.e. the rate of intrinsic zeroth order without co-absorption) as well as $2\beta\alpha^{-1} = \varepsilon_p(\varepsilon_{cat}c_{cat})^{-1}$ = 0,0140 μM^{-1}.

As the share of photons reaching at the end of completed one-fold reduction of MV^{2+} can be expressed in the form of equation 13,

$$f_{cat,end} = \frac{1}{1 + \left(\frac{\varepsilon_P}{\varepsilon_{cat}c_{cat}}\right)S_0} \qquad (eq.13)$$

the fitted data allow to give values for $f_{cat,end}$ of 0.59 (50 µM MV^{2+} starting concentration), 0.42 (100 µM MV^{2+} starting concentration) and 0.26 (200 µM MV^{2+} starting concentration) as well as pure times for complete substrate conversion T´ if no co-absorption by $MV^{•+}$ would occur, calculated by equation 14:

$$T' = \frac{S_0}{k} \qquad (eq.14)$$

For 50 µM, 100 µM, and 200 µM MV^{2+}, 6.6 s, 13.1 s and 26. s for T´ are obtained.

When using $c_{cat} = c(^{CF3}\mathbf{Ru}) = 5\ \mu M$ as well as $\varepsilon_P(463\ nm) \approx 1300\ Lmol^{-1}cm^{-1}$,[11] then $2\beta\alpha^{-1} = \varepsilon_p(\varepsilon_{cat}c_{cat})^{-1} = 0{,}0140\ \mu M^{-1}$ can be transformed into equation 15:

$$\varepsilon_{cat}(463\text{nm}) \approx \frac{\varepsilon_P}{0{,}01402 c_{cat}} \approx 1{,}85 \cdot 10^4\ \text{Lmol}^{-1}\text{cm}^{-1} \qquad (eq. 15)$$

As the extinction coefficient of ^{CF3}**Ru** at 463 nm predicted by this model of ca. 18˙500 $Lmol^{-1}cm^{-1}$ is closely matching the reported values[4] and the recorded data presented in Figure S22, this verifies the model which is based on the assumption of a zeroth order rate constant. Thus, the rate determining step of the overall photocatalytic MV^{2+} reduction is – at least for the first reduction step that generates $MV^{\bullet+}$ – the reductive quenching step.

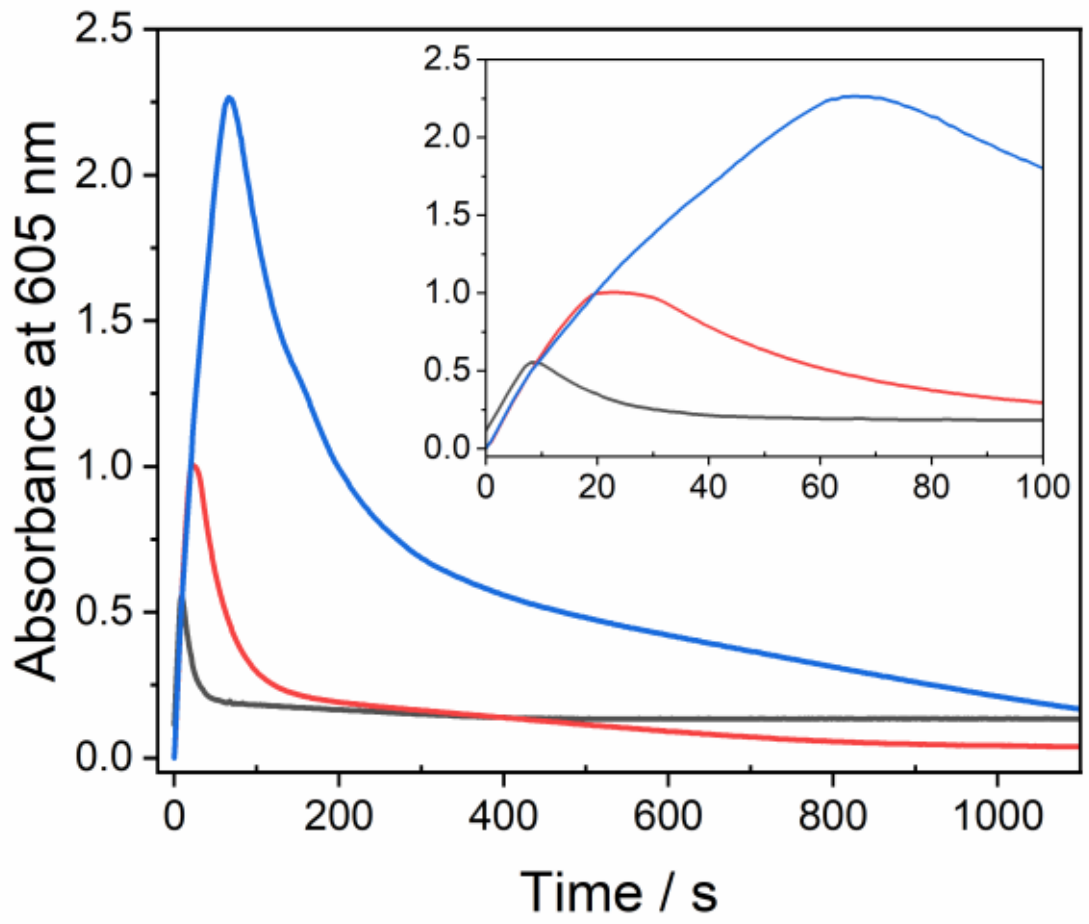

Figure 21. Absorbance changes at 605 nm with increasing irradiation time during MV^{2+} photoreduction using 5 µM ^{CF3}**Ru** in MeCN:H_2O = 1:1 (v:v) with varying MV^{2+} starting concentrations of 50 µM (black curve), 100 µM (red curve) and 200 µM (blue curve). Maxima were reached after 8 s, 23 s and 63 s, respectively. This correlates to QYs for complete monoreduction of ca. 17 %, 12 %, and 9.0 %, respectively highlighting the effect of co-absorption.

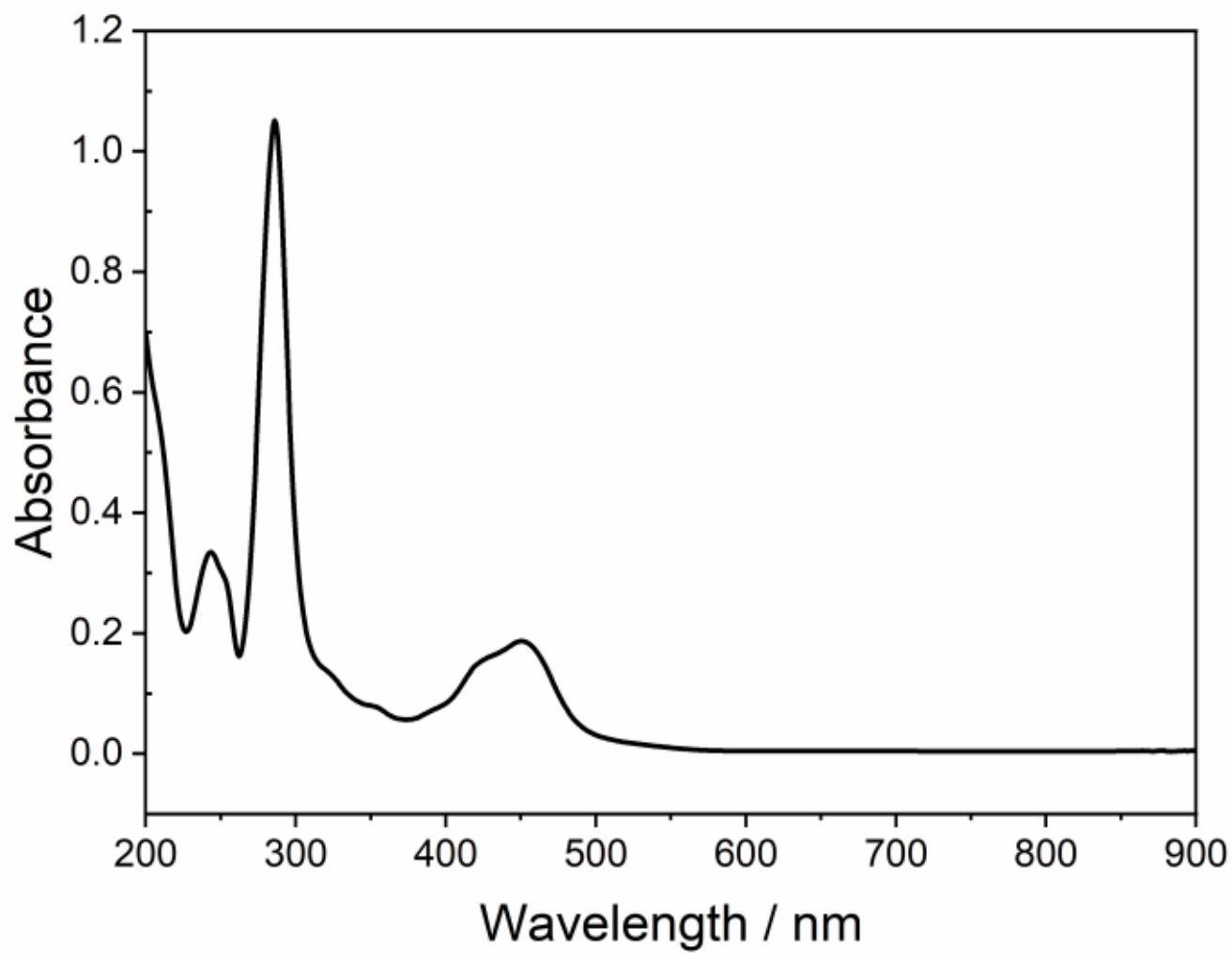

Figure S22. Steady-state UV/vis absorption spectrum of ^{CF3}**Ru** (10 µM) in MeCN.

5. Theory part

5.1. Computational details

A dimer structure, comprised of a ruthenium complex (^{H}**Ru** or ^{CF3}**Ru**) and MV, with a net charge of 2+, was optimized at the DFT level of theory, using the Gaussian 16 program (Revision C.02).[12] The dimers were optimized in both the singlet and triplet multiplicity, reflecting the product ($^{CF3/H}$**Ru^{2+}** + MV^0) and reactant ($^{CF3/H}$**Ru^{+}** + $MV^{\bullet+}$) redox states of the species, respectively (see Table S4). Unless stated otherwise, all DFT calculations utilized the B3LYP functional with the split valence def2svp basis set.[13] To account for long-range interactions, Grimme's D3 dispersion correction with Becke-Johnson damping was employed[14] and implicit solvation (water or MeCN) was incorporated with the SMD model of the integral equation formalism.[15] All minima reported were verified via frequency calculations, revealing no negative frequencies.

5.2. Initial optimization

The singlet ground state, S_0 of a dimer (with a net 2+ charge) comprised of the Ru complex (^{CF3}**Ru** or ^{H}**Ru**) and MV, was optimized at the DFT level of theory in an implicit water solvent field. In this optimized S_0 geometry, the respective triplet energy was obtained via a single point energy calculation, thus allowing the singlet-triplet energy gap ($\Delta E = S_0 - T_1$) to be determined at the given geometry. The relaxed triplet geometry (T_1) was obtained using the same procedure and the $S_0 - T_1$ energy gap obtained within the T_1 geometry. This methodology was also repeated in an implicit MeCN solvent field.

5.3. Explicit solvation

The program PackMol[16] was utilized to distribute 200 molecules of H_2O in a sphere with radius of 12 Å around the dimer. The resulting structure was then optimized using the XTB program (xtb version 6.7.1) with the GFN2-xTB method.[17,18] Restrictions were placed on the dimer system, using the 'fix atoms' command such that only the 200 H_2O molecules were able to relax around the dimer preoptimized using DFT. This process was repeated for the ^{CF3}**Ru**/MV dimer in explicit MeCN.

5.4. Molecular Dynamics simulations

In order to explore a range of dimer configurations, molecular dynamics (MD) simulations were employed, utilizing the *ab initio* MD module of Orca6.1.[19] The MD simulations were performed using the semiempirical GFN2-xTB method,[17,18] with a timestep of 1.0 fs, a NHC thermostat with a temperature of 298 K with a relaxation time (Timecon) modifier of 20 fs. All simulations were performed with singlet multiplicity and a net charge of +2, i.e. within the $^{CF3/H}$**Ru^{2+}** + MV^0 state.

MD simulations were run with both implicit and explicit solvent fields. In the implicit simulations, the starting structure was taken from the DFT optimized dimer geometry and the implicit solvent field (water or MeCN) was employed using the *alpb* keyword. A cell sphere with radius of 50 Å with a spring constant of 10 kJ mol^{-1} $Å^{-2}$ was utilized to keep the Ru complex and MV from drifting too far apart. The implicit solvent simulations were run for a minimum of 10,000 fs.

When explicit solvation was incorporated into the MD simulations, the starting geometry (t = 0 fs) was used from the previously described XTB optimization (section 3.3). As 200 solvent molecules were involved in the simulation, no additional implicit solvent field was utilized. In addition to the same cell sphere restraints applied in the implicit simulations, a center restraint was applied to all atoms in the Ru complex, to fix the center of mass and thereby limit movement outside of the explicit solvent sphere. The explicit solvent simulations were run for a minimum of 6000 fs.

Following the MD runs, the cartesian coordinates of the system (including explicit solvent) were sampled at 500 fs intervals and single point singlet and triplet DFT calculations employing the same parameters described in section 3.1, were performed. The singlet and triplet energy difference at each 500 fs time interval was calculated, to approximate the driving force for the second reduction process.

5.5. Method utilized to model the second reduction process

As described in section 3.2, the structure of the **^{H}Ru**/MV dimer was initially optimized with a net charge of +2 and a multiplicity of 1. In this singlet configuration, the HOMO and LUMO are localized on the MV and the **^{H}Ru** respectively. Using the Multiwfn program,[20,21] atomic dipole moment corrected Hirshfeld population (ADCH) method[22] was used to show that the majority of the net charge (+1.78) was localized on **^{H}Ru**. Contrastingly, when an unrestricted single point energy calculation of triplet multiplicity is performed on the same geometry, the charge on the **^{H}Ru** decreases to +1.04 and the charge on the MV increases from +0.22 to +0.96. Furthermore, analysis of the spin density of the single point triplet calculation (within the S_0 optimized geometry) reveals spin density localized on both the MV and **^{H}Ru**. The same procedure was applied to the **^{CF3}Ru**/MV dimer, which exhibited very similar behavior.

In summary, performing (restricted) singlet calculation of the dimer effectively models the 'product' state of the reduction process, namely the doubly reduced MV^0 and Ru^{2+} (**^{CF3}Ru** or **^{H}Ru**). In the triplet multiplicity, the 'reactant' state is given, comprised of the singly reduced $MV^{\bullet+}$ and Ru^{+}. Through comparison of the relative energies of the singlet and triplet (product and reactant) at a given geometry, the thermodynamic favorability of the second reduction process can be assessed.

Table S3. Illustration of the HOMO and LUMO of the ^{CF3}**Ru**/MV and ^{H}**Ru**/MV dimers in their optimized S_0 geometries within an implicit H_2O solvent field. The spin density is also given in the triplet multiplicity.

Ru/MV dimer	Opt. geom.	HOMO	LUMO	Spin density
^{CF3}**Ru**	S_0	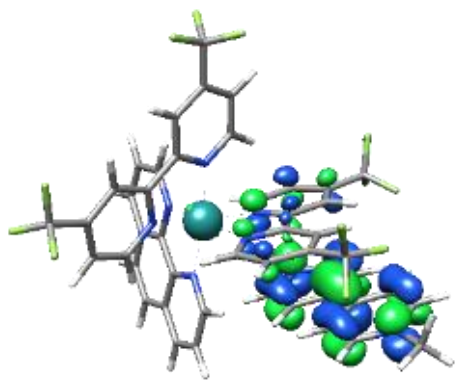	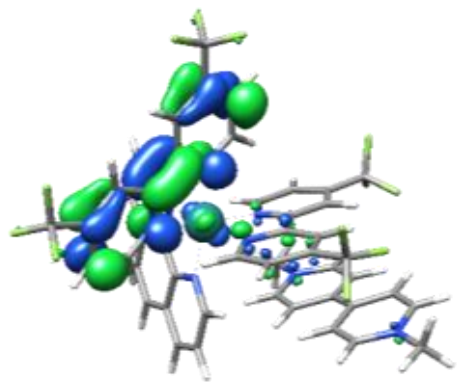	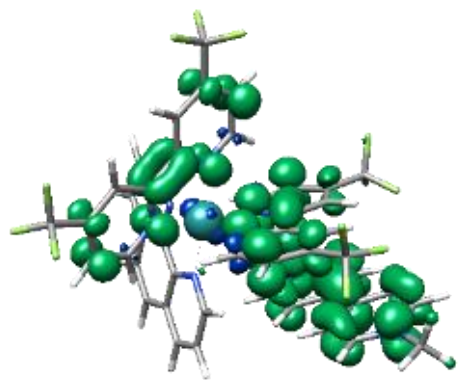
^{H}**Ru**	S_0	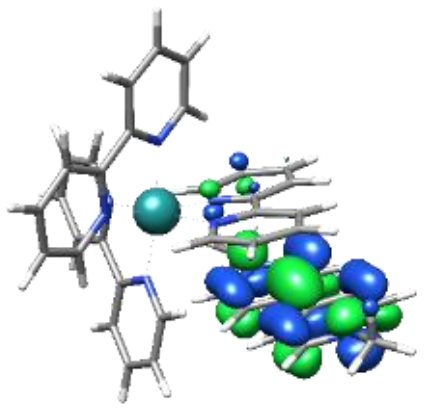	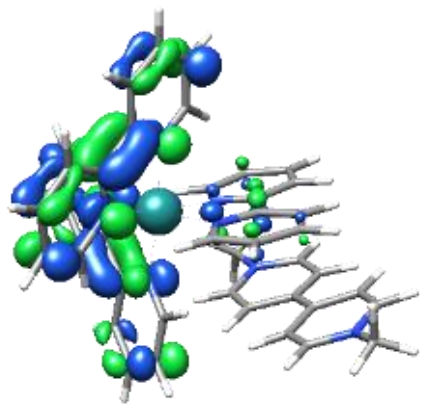	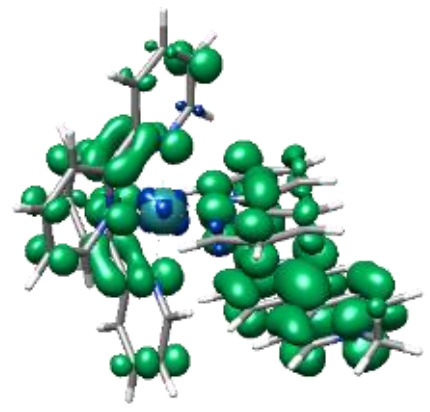

Table S4. DFT single point energies of the ^{CF3}**Ru**/MV and ^{H}**Ru**/MV dimers in implicit solvation in the DFT optimized S_0 and T_1 geometries, where $\Delta E_{ST} = E_S - E_T$. The ADCH charges are reported as a sum of the atoms in the Ru complex and the MV.

Ru/MV dimer	Opt. geom.	Multiplicity	Implicit solvent	Energy / eV	ΔE_{ST} / eV	ADCH Ru	ADCH MV
^{CF3}**Ru**	S_0	S	H_2O	0.00	-0.25	+1.59	+0.41
		T		0.25		+1.08	+0.92
	T_1	S	H_2O	0.25	+0.19	+1.49	+0.51
		T		0.06		+1.08	+0.92
^{H}**Ru**	S_0	S	H_2O	0.00	-0.61	+1.78	+0.22
		T		0.61		+1.04	+0.96
	T_1	S	H_2O	0.30	-0.01	+1.85	+0.15
		T		0.31		+1.08	+0.92
^{CF3}**Ru**	S_0	S	MeCN	0.00	-0.24	+1.61	+0.39
		T		0.24		+1.08	+0.92
	T_1	S	MeCN	0.22	+0.19	+1.47	+0.53
		T		0.03		+1.10	+0.90
^{H}**Ru**	S_0	S	MeCN	0.00	-0.61	+1.79	+0.21
		T		0.61		+1.02	+0.98
	T_1	S	MeCN	0.32	+0.01	+1.85	+0.15
		T		0.31		+1.08	+0.92

Table S5. DFT single point energies of the **^{CF3}Ru**/MV and **^{H}Ru**/MV dimers in explicit solvation (200 H_2O or MeCN molecules) obtained from the XTB optimized S_0 geometry, where $\Delta E_{ST} = E_S - E_T$. The ADCH charges are reported as a sum of the atoms in the Ru complex and the MV. The remaining charge (net +2) is associated with the solvent molecules.

Ru/MV dimer	Opt. geom.	Multiplicity	Explicit solvent	Energy / eV	ΔE_{ST} / eV	ADCH Ru	ADCH MV	ADCH solvent
**^{CF3}Ru**	S_0	S	H_2O	0.00	-0.24	+0.90	+0.18	+0.92
		T		0.24		+0.58	+0.49	+0.93
**^{H}Ru**	S_0	S	H_2O	0.00	-0.87	+0.89	+0.02	+1.09
		T		0.87		+0.34	+0.58	+0.58
**^{CF3}Ru**	S_0	S	MeCN	0.00	-0.11	+0.97	+0.29	+1.02
		T		0.11		+0.74	+0.52	+0.74
**^{H}Ru**	S_0	S	MeCN	0.00	-0.94	+1.12	+0.07	+0.81
		T		0.94		+0.45	+0.63	+0.92

Table S6. Illustration of the HOMO and LUMO of the $^{\mathrm{CF3}}$**Ru**/MV and ^{H}**Ru**/MV dimers in their optimized S_0 geometries within an explicit H_2O or MeCN solvent field. The spin density is also given in the triplet multiplicity.

Ru/MV dimer	HOMO	LUMO	Spin density
$^{\mathrm{CF3}}$**Ru** (H_2O)	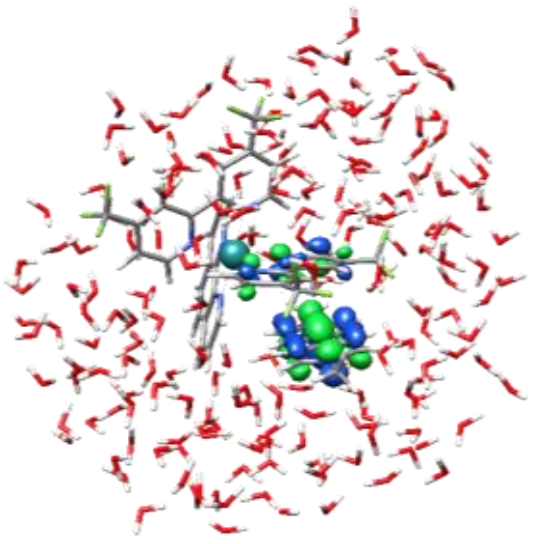	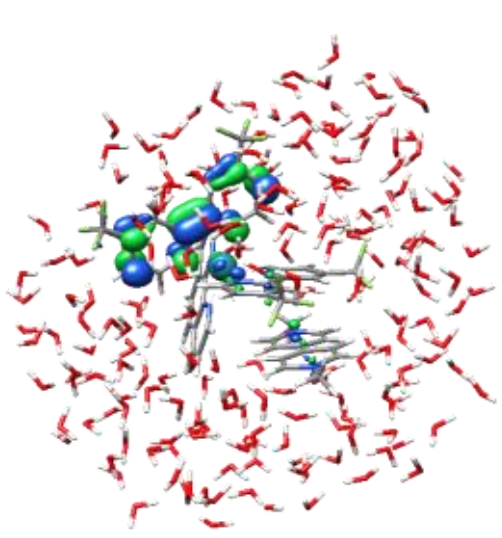	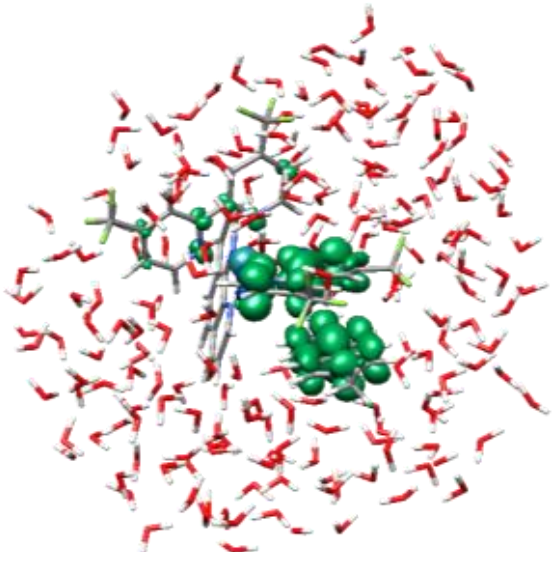
^{H}**Ru** (H_2O)	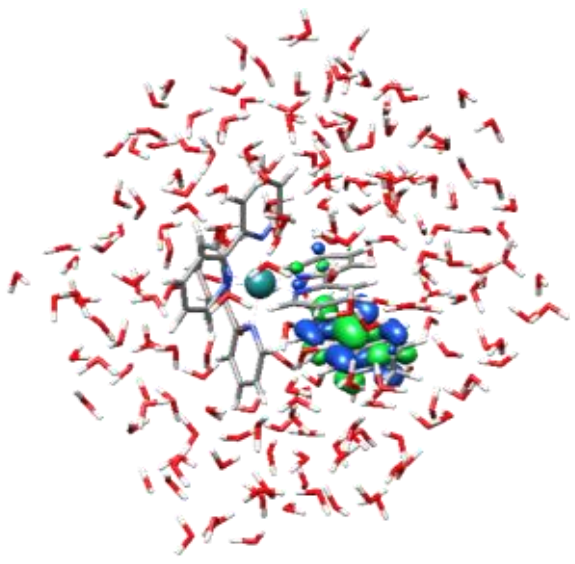	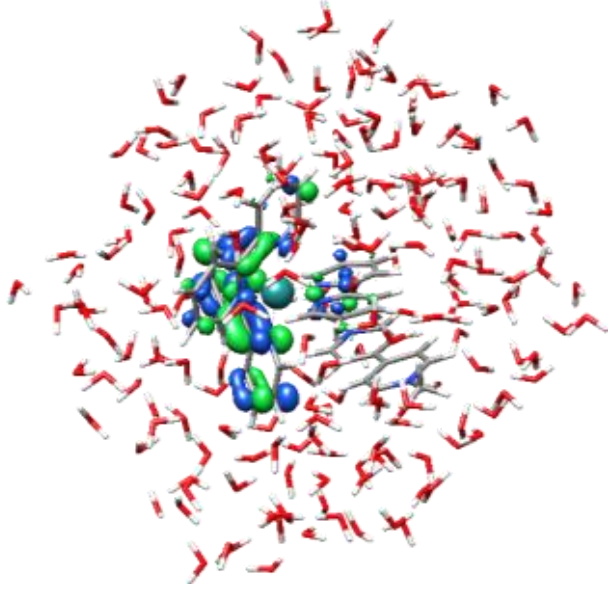	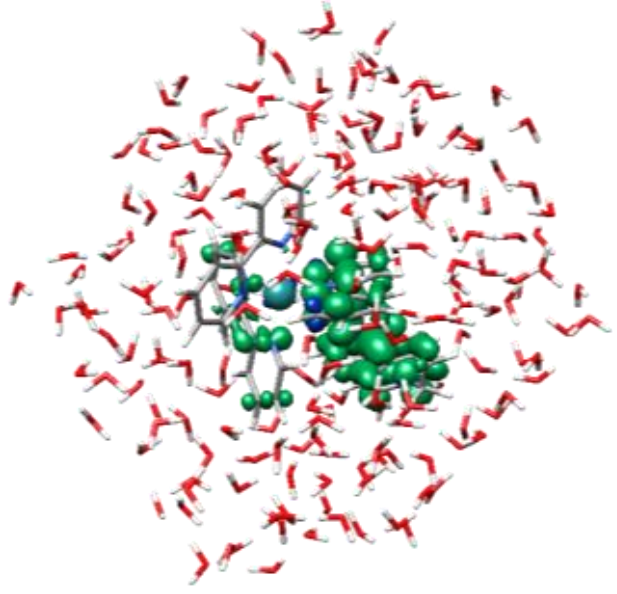
$^{\mathrm{CF3}}$**Ru** (MeCN)	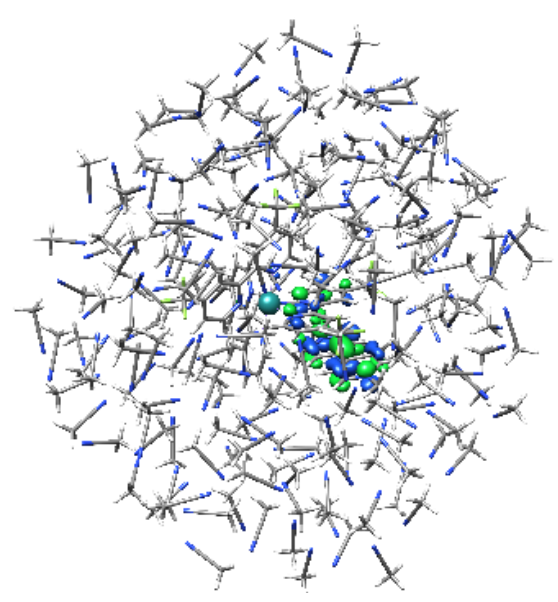	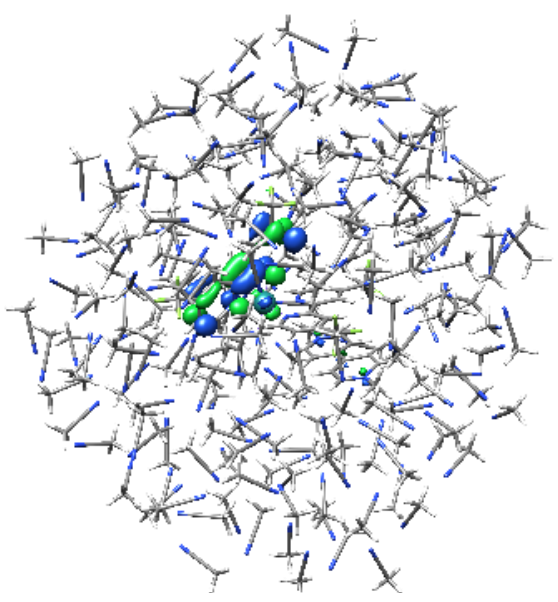	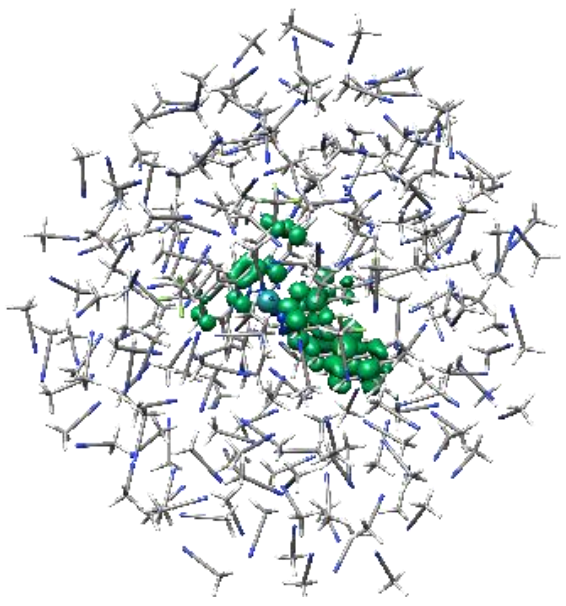
^{H}**Ru** (MeCN)	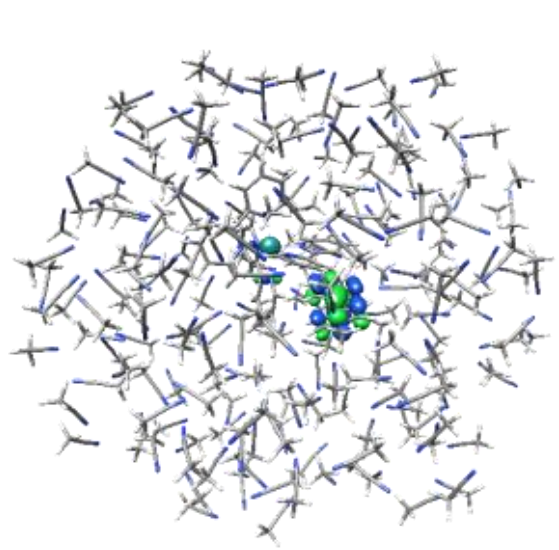	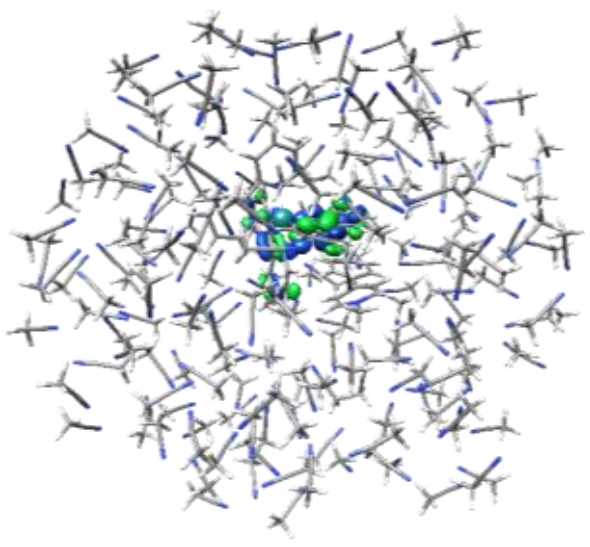	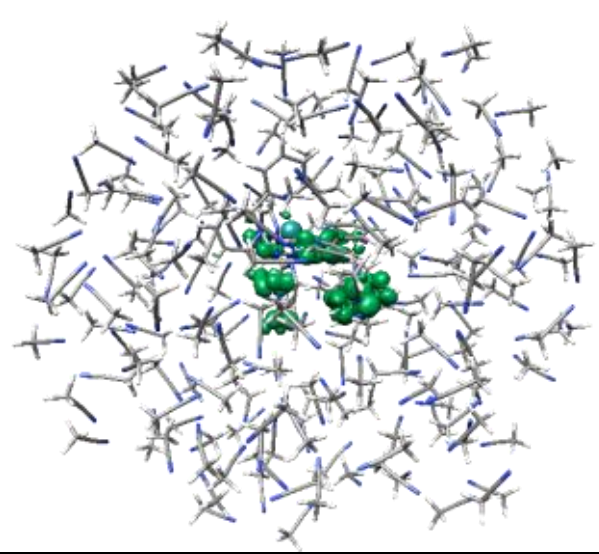

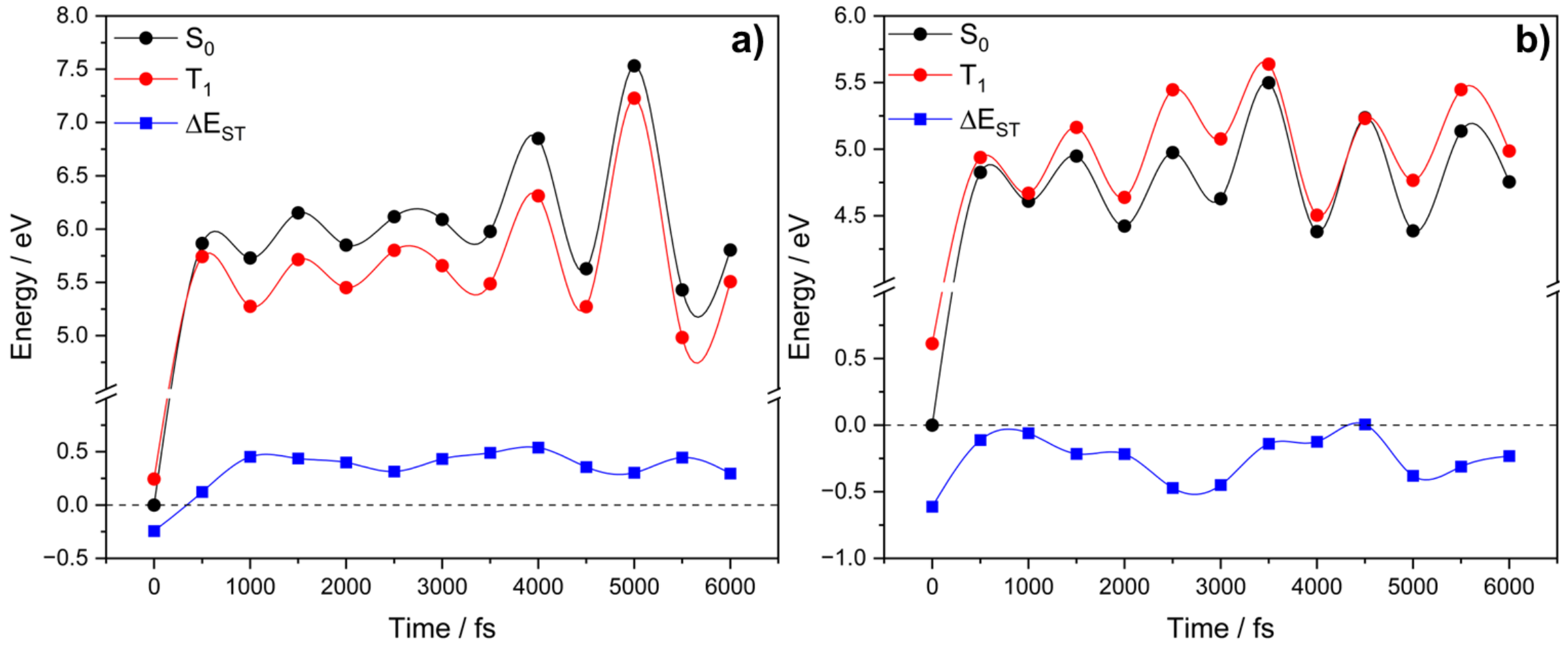

Figure S23. Singlet (black; $^{CF3/H}$**Ru**$^{2+}$ + MV^0) and triplet (red; $^{CF3/H}$**Ru**$^{+}$ + $MV^{\bullet+}$) single point DFT energies obtained for the a) ^{CF3}**Ru**/MV and b) ^{H}**Ru**/MV dimers in an implicit MeCN solvent field. The geometry snapshots obtained are obtained at 500 fs intervals from the MD simulations, starting from the DFT optimized geometry at t = 0.00 fs. The blue line corresponds to $\Delta E_{ST} = E_S - E_T$. When $\Delta E_{ST} < 0$ the forward reaction process for the reduction of $MV^{\bullet+}$ to MV^0 is thermodynamically favored.

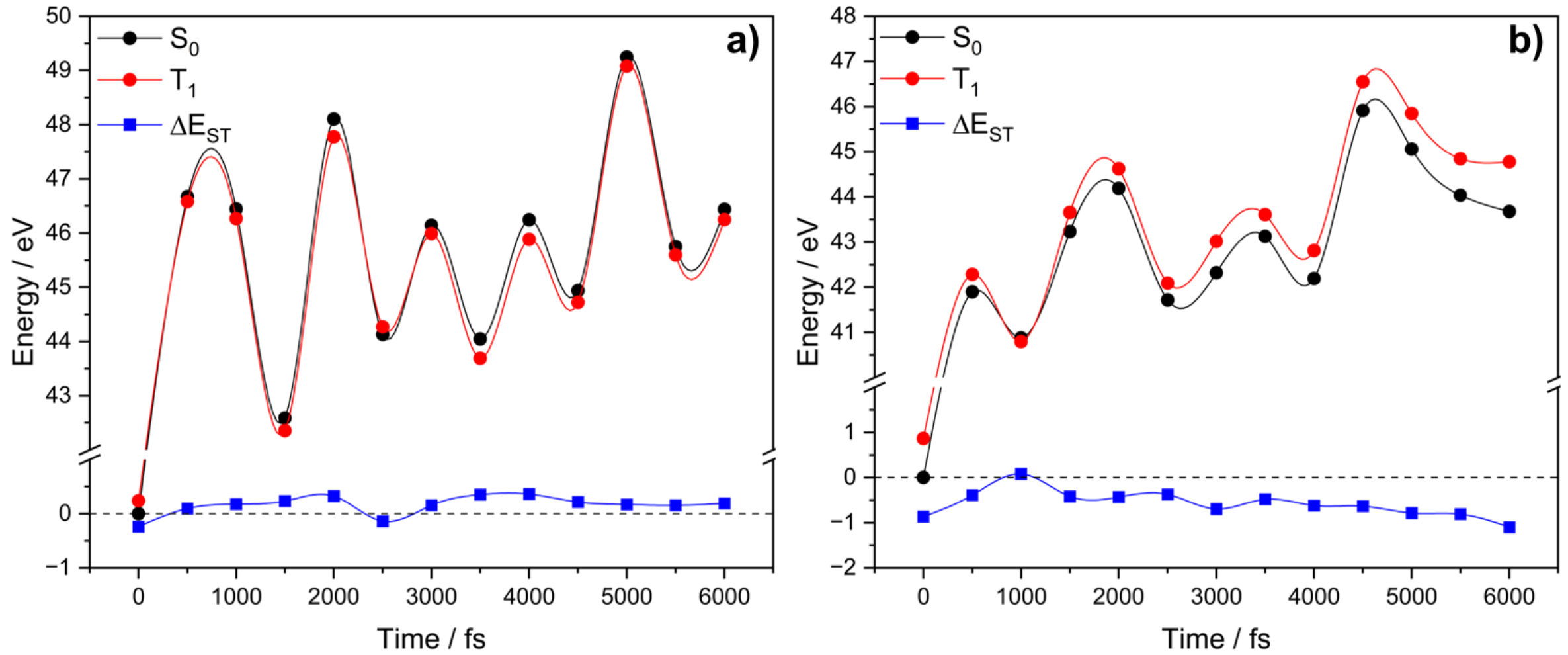

Figure S24. Singlet (black; $^{CF3/H}$**Ru**$^{2+}$ + MV^0) and triplet (red; $^{CF3/H}$**Ru**$^{+}$ + $MV^{\bullet+}$) single point DFT energies obtained for the a) ^{CF3}**Ru**/MV and b) ^{H}**Ru**/MV dimers in an explicit solvent field comprised of 200 H_2O molecules. The geometry snapshots obtained are obtained at 500 fs intervals from the MD simulations, starting from the DFT/XTB optimized geometry at t = 0.00 fs. The blue line corresponds to $\Delta E_{ST} = E_S - E_T$. When $\Delta E_{ST} < 0$ the forward reaction process for the reduction of $MV^{\bullet+}$ to MV^0 is thermodynamically favored.

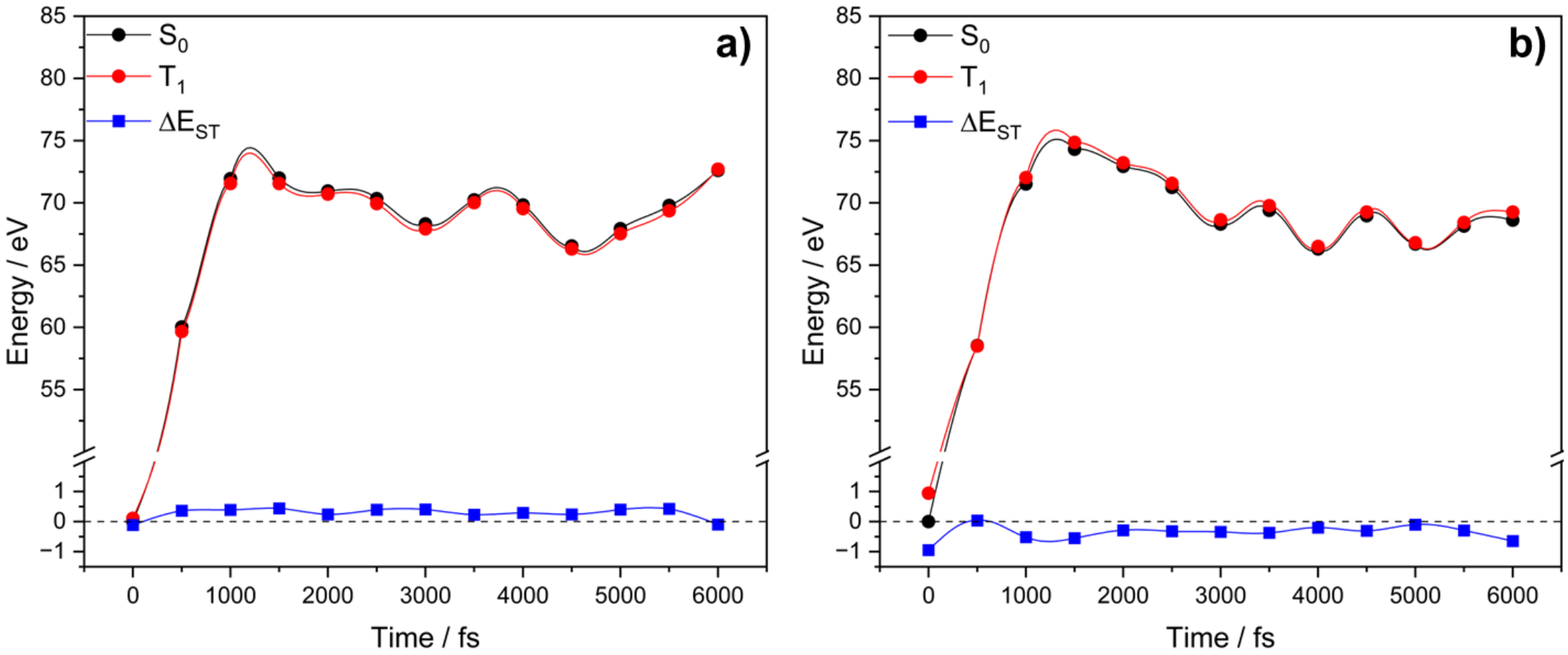

Figure S25. Singlet (black; $^{CF3/H}$**Ru**$^{2+}$ + MV^0) and triplet (red; $^{CF3/H}$**Ru**$^{+}$ + $MV^{\bullet+}$) single point DFT energies obtained for the a) ^{CF3}**Ru**/MV and b) ^{H}**Ru**/MV dimers in an explicit solvent field comprised of 200 MeCN molecules. The geometry snapshots obtained are obtained at 500 fs intervals from the MD simulations, starting from the DFT/XTB optimized geometry at t = 0.00 fs. The blue line corresponds to $\Delta E_{ST} = E_S - E_T$. When $\Delta E_{ST} < 0$ the forward reaction process for the reduction of $MV^{\bullet+}$ to MV^0 is thermodynamically favored.

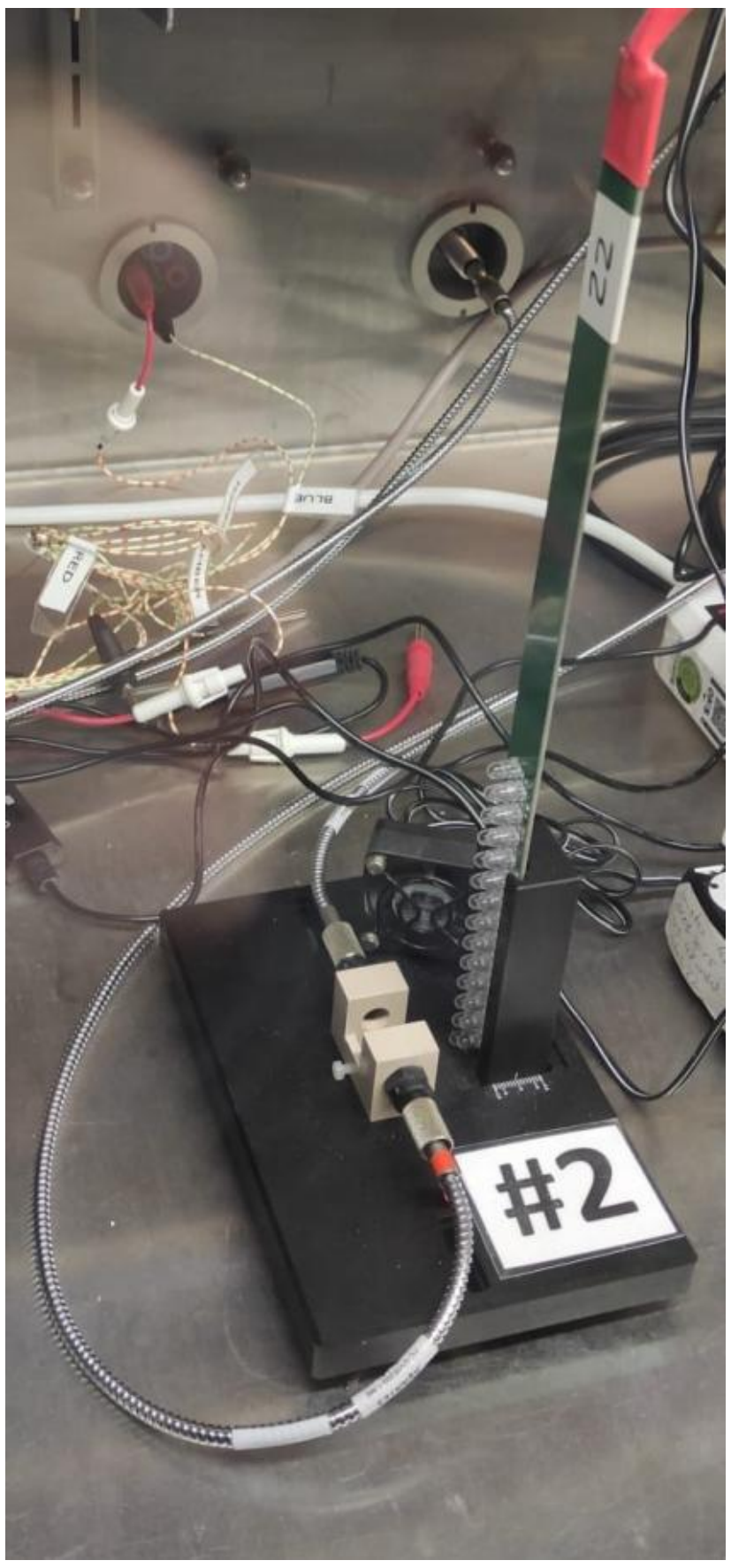

Figure S26. Photograph of the experimental setup inside an Ar-filled GB for rapid UV-vis absorption spectroscopy following photocatalytic processes with an Avantes AvaSpec-ULS2048CL detector unit coupled with an AVA AvaLight-DH-S-BAL light source. The front of the LED-stick has a distance of ca. 1.0 cm to the cuvette surface.

6. References

[1] C. E. Paul, S. Gargiulo, D. J. Opperman, I. Lavandera, V. Gotor-Fernández, V. Gotor, A. Taglieber, I. W. C. E. Arends, F. Hollmann “Mimicking Nature: Synthetic Nicotinamide Cofactors for C═C Bioreduction Using Enoate Reductases” *Org. Lett.* **2013**, *15*, 180–183.

[2] C. Müller, I. Friedländer, B. Bagemihl, S. Rau, B. Dietzek-Ivanšić “The electron that breaks the catalyst’s back – excited state dynamics in intermediates of molecular photocatalysts” *Phys. Chem. Chem. Phys.* **2021**, *23*, 27397–27403.

[3] T. Janoschka, N. Martin, U. Martin, C. Friebe, S. Morgenstern, H. Hiller, M. D. Hager, U. S. Schubert “An aqueous, polymer-based redox-flow battery using non-corrosive, safe, and low-cost materials” *Nature* **2015**, *527*, 78–81.

[4] H. D. Cole, A. Vali, J. A. I. Roque, G. Shi, A. Talgatov, G. Kaur, A. Francés-Monerris, M. E. Alberto, C. G. Cameron, S. A. McFarland “Ru(II) Oligothienyl Complexes with Fluorinated Ligands: Photophysical, Electrochemical, and Photobiological Properties” *Inorg. Chem.* **2024**, *63*, 9735–9752.

[5] V. V. Pavlishchuk, A. W. Addison “Conversion constants for redox potentials measured versus different reference electrodes in acetonitrile solutions at 25°C” *Inorganica Chim. Acta* **2000**, *298*, 97–102.

[6] R. Lomoth, T. Häupl, O. Johansson, L. Hammarström “Redox-Switchable Direction of Photoinduced Electron Transfer in an $Ru(bpy)_3^{2+}$-Viologen Dyad” *Chem. - Eur. J.* **2002**, *8*, 102–110.

[7] D. M. Arias-Rotondo, J. K. McCusker “The photophysics of photoredox catalysis: a roadmap for catalyst design” *Chem. Soc. Rev.* **2016**, *45*, 5803–5820.

[8] Y. Pellegrin, F. Odobel “Sacrificial electron donor reagents for solar fuel production” *C R Chim* **2017**, *20*, 283–295.

[9] H. Zhu, N. Song, H. Lv, C. L. Hill, T. Lian “Near Unity Quantum Yield of Light-Driven Redox Mediator Reduction and Efficient H_2 Generation Using Colloidal Nanorod Heterostructures” *J. Am. Chem. Soc.* **2012**, *134*, 11701–11708.

[10] M. T. Nenadović, O. I. Mićić, T. Rajh, D. Savić “Temperature Effect on the Photoinduced Reduction of Methyl Viologen with Several Sensitizers and the Evolution of Hydrogen from Water” *J. Photochem.* **1983**, *21*, 35–44.

[11] M. Rougee, T. Ebbessen, F. Ghetti, R. V. Bensasson “Kinetics and Mechanism of Porphyrin-Photosensitized Reduction of Methylviologen” *J. Phys. Chem.* **1982**, *86*, 4404–4412.

[12] M. J. Frisch, G. W. Trucks, H. B. Schlegel, G. E. Scuseria, M. A. Robb, J. R. Cheeseman, G. Scalmani, V. Barone, G. A. Petersson, H. Nakatsuji, X. Li, M. Caricato, A. V. Marenich, J. Bloino, B. G. Janesko, R. Gomperts, B. Mennucci, H. P. Hratchian, J. V. Ortiz, A. F. Izmaylov, J. L. Sonnenberg, D. Williams-Young, F. Ding, F. Lipparini, F. Egidi, J. Goings, B. Peng, A. Petrone, T. Henderson, D. Ranasinghe, V. G. Zakrzewski, J. Gao, N. Rega, G. Zheng, W. Liang, M. Hada, M. Ehara, K. Toyota, R. Fukuda, J. Hasegawa, M. Ishida, T. Nakajima, Y. Honda, O. Kitao, H. Nakai, T. Vreven, K. Throssell, Montgomery Jr. J. A., J. E. Peralta, F. Ogliaro, M. J. Bearpark, J. J. Heyd, E. N. Brothers, K. N. Kudin, V. N. Staroverov, T. A. Keith, R. Kobayashi, J. Normand, K. Raghavachari, A. P. Rendell, J. C. Burant, S. S. Iyengar, J. Tomasi, M. Cossi, J. M. Millam, M. Klene, C. Adamo, R. Cammi, J. W. Ochterski, R. L. Martin, K. Morokuma, O. Farkas, J. B. Foresman, D. J. Fox **2016**.

[13] F. Weigend, R. Ahlrichs “Balanced basis sets of split valence, triple zeta valence and quadruple zeta valence quality for H to Rn: Design and assessment of accuracy” *Phys. Chem. Chem. Phys.* **2005**, *7*, 3297–3305.

[14] S. Grimme, S. Ehrlich, L. Goerigk “Effect of the Damping Function in Dispersion Corrected Density Functional Theory” *J. Comput. Chem.* **2011**, *32*, 1456–1465.

[15] A. V. Marenich, C. J. Cramer, D. G. Truhlar "Universal Solvation Model Based on Solute Electron Density and on a Continuum Model of the Solvent Defined by the Bulk Dielectric Constant and Atomic Surface Tensions" *J. Phys. Chem. B* **2009**, *113*, 6378–6396.
[16] L. Martínez, R. Andrade, E. G. Birgin, J. M. Martínez "PACKMOL: A Package for Building Initial Configurations for Molecular Dynamics Simulations" *J. Comput. Chem.* **2009**, *30*, 2157–2164.
[17] C. Bannwarth, S. Ehlert, S. Grimme "GFN2-xTB—An Accurate and Broadly Parametrized Self-Consistent Tight-Binding Quantum Chemical Method with Multipole Electrostatics and Density-Dependent Dispersion Contributions" *J. Chem. Theory Comput.* **2019**, *15*, 1652–1671.
[18] C. Bannwarth, E. Caldeweyher, S. Ehlert, A. Hansen, P. Pracht, J. Seibert, S. Spicher, S. Grimme "Extended tight-binding quantum chemistry methods" *WIREs Comput. Mol. Sci.* **2021**, *11*, e1493.
[19] F. Neese, F. Wennmohs, U. Becker, C. Riplinger "The ORCA quantum chemistry program package" *J. Chem. Phys.* **2020**, *152*, 224108.
[20] T. Lu, F. Chen "Multiwfn: A Multifunctional Wavefunction Analyzer" *J. Comput. Chem.* **2012**, *33*, 580–592.
[21] T. Lu "A comprehensive electron wavefunction analysis toolbox for chemists, Multiwfn" *J. Chem. Phys.* **2024**, *161*, 082503.
[22] T. Lu, F. Chen "Atomic Dipole Moment Corrected Hirshfeld Population Method" *J. Theor. Comput. Chem.* **2012**, *11*, 163–183.
